# High-Grade Salivary-Gland Involvement, Assessed by Histology or Ultrasonography, Is Associated with a Poor Response to a Single Rituximab Course in Primary Sjögren’s Syndrome: Data from the TEARS Randomized Trial

**DOI:** 10.1371/journal.pone.0162787

**Published:** 2016-09-23

**Authors:** Divi Cornec, Sandrine Jousse-Joulin, Sebastian Costa, Thierry Marhadour, Pascale Marcorelles, Jean-Marie Berthelot, Eric Hachulla, Pierre-Yves Hatron, Vincent Goeb, Olivier Vittecoq, Emmanuel Nowak, Jacques-Olivier Pers, Valérie Devauchelle-Pensec, Alain Saraux

**Affiliations:** 1 Service de Rhumatologie, Hôpital de la Cavale Blanche, CHRU Brest, Boulevard Tanguy Prigent, 29609 Brest, France; 2 EA2216, INSERM ESPRI, ERI29, Laboratoire d’Immunothérapies et Pathologies lymphocytaires B, Université de Brest, and Labex "IGO", Brest, France; 3 Laboratoire d’Anatomie Pathologique et Cytologie, Hôpital Morvan, CHRU Brest, Avenue Foch, 29609 Brest, France; 4 Service de Rhumatologie, Hôtel-Dieu, CHU Nantes, 44093 Nantes Cedex 01, France; 5 Service de Médecine Interne, Claude Huriez Hospital, Université Lille Nord-de-France, 59037 Lille Cedex, France; 6 Service de Rhumatologie, CHRU de Rouen, 76230 Bois-Guillaume, France; 7 INSERM CIC 0502, CHU Brest, Brest, France; Center for Rheumatic Diseases, INDIA

## Abstract

**Purpose:**

To determine whether the severity of salivary-gland involvement, assessed using salivary gland ultrasonography [SGUS], histological focus score, or the unstimulated whole salivary flow [UWSF], was associated with the response to rituximab in patients with primary Sjögren’s syndrome [pSS].

**Materials and Methods:**

Among the 120 patients with pSS enrolled in the randomised TEARS trial of rituximab versus placebo, 35 underwent either centralised minor salivary-gland biopsy or SGUS at inclusion. The echostructure of each parotid and submandibular gland was graded on a scale of 0 to 4. Histologic minor salivary gland involvement was assessed by the focus score. Among rituximab-treated patients with available data (n = 14), half met the Sjögren’s Syndrome Responder Index [SSRI]-30 definition of a response at week 24.

**Results:**

The SGUS score correlated positively to the focus score [r = 0.61] and negatively to the UWSF [r = -0.68]. The focus score was not correlated to the UWSF. The median total SGUS grade at inclusion was 9 [6-11] in responders versus 16 [11-16] in non-responders [*p* = 0.04]. The proportion of SSRI-30 responders was 0% among patients with SGUS grade 4 and 88% among those with SGUS grade ≤3. Low baseline SGUS scores were associated with sicca-related outcomes improvement, but not with fatigue or biological improvement. Median baseline focus score was 0.3 [0.0–1.3] in the responders versus 4.0 [2.7–5.3] in the non-responders [*p* = 0.02]. Baseline UWSF was not associated with the response rate.

**Conclusion:**

In patients with pSS, the highest SGUS grade or a high histological focus score is associated with absence of a response to a single rituximab course after 6 months. Further studies, including more patients and different treatment strategies, are required to confirm the clinical utility of these potential biomarkers in pSS.

## Introduction

Primary Sjögren’s syndrome [pSS] is a systemic autoimmune disease clinically characterised by intense fatigue and by oral and ocular dryness, reflecting lymphocytic infiltration and subsequent dysfunction of the exocrine glands. Its prevalence is estimated at about 40/100 000 inhabitants in Europe [1].

No treatments have been proven to modify the natural course of the disease. The few large randomised controlled studies in patients with pSS showed no significant efficacy of TNF-alpha antagonists [2,3] or hydroxychloroquine [4]. Recent evidence suggested benefits from rituximab therapy [5]. However, the larger TEARS trial [6] failed to detect a therapeutic effect of rituximab, indicating either lack of efficacy of this agent or low sensitivity to change of the primary endpoint. We recently developed the Sjögren’s Syndrome Response Index (SSRI)-30, a data-driven composite index of treatment response including symptoms (oral and ocular dryness and fatigue) and objective measurements (salivary flow and erythrocyte sedimentation rate) [7]. In the TEARS trial, about half the rituximab-treated patients achieved the SSRI-30 versus 10–20% in the placebo arm [7].

A major goal of research in the field is now the identification of specific baseline characteristics/biomarkers to predict the response to therapy. The level of involvement of the target organs could be determinant in the efficacy of treatments. In a previous study [8], only patients with total stimulated salivary flow >0.1 ml/min displayed a functional salivary gland improvement after rituximab.

Salivary gland ultrasonography (SGUS) is being widely used for pSS diagnosis [9–17]. The main pathological feature is the presence of hypoechoic area. An international expert group has been recently created in order to standardize the procedure, in the ultimate goal of including SGUS as an additional item in future classification criteria [18]. Besides this diagnostic value, we have previously shown that parotid ultrasonographic score (based on the number and size of hypoechoic area) improved in half of the rituximab-treated patients in the TEARS trial, compared to 7% of the patients allocated to the placebo [19]. SGUS score is correlated with disease activity [11,12]. Consequently, SGUS could also be a biomarker predictive of the response to therapy.

Finally, several articles reported that the intensity of the histological inflammation, represented by the focus score, is associated to disease activity and has a prognostic value for disease severity [20,21]. Thus, the intensity of salivary gland inflammation, assessed by the focus score, could be associated with the response to an active treatment [22].

Our objective here was to determine whether the severity of salivary-gland involvement as assessed morphologically [SGUS], histologically, or functionally [salivary flow], predicted the response to rituximab in patients with pSS.

## Patients and Methods

### Patients

The TEARS study [6] was a multicentre double-blind randomised controlled trial performed in France between March 6, 2008 and January 5, 2011 to compare rituximab [two 1-g infusions two weeks apart] and a placebo in 122 patients meeting AECG criteria for pSS [23]. Randomization was stratified by site. A computer-generated random allocation sequence was prepared by our statistics department in Brest. All patients had either recent-onset [<10 years] and biologically active disease [presence of auto-antibodies or B-cell activation markers] or systemic disease. Patients were followed until week [W] 24. The detailed description of the TEARS trial protocol and results has been already published [6], the detailed protocol is available on PlosOne website as supporting information (S1 Checklist, S1 and S2 Protocols). The TEARS trial was approved by the appropriate ethics committee [Comité de Protection des Personnes (CPP) Ouest VI, #2007/493] in 2007, prior to first patient recruitment. All patients gave their signed written informed consent to participate in the TEARS study, as approved by the ethics committee. The TEARS study was registered on ClinicalTrials (ClinicalTrials.gov: NCT00740948) in August 2008 soon after the enrolment of the first patients, since this registration as not required by French authorities before the beginning of a trial at this time. The authors confirm that all ongoing and related trials for this intervention are registered.

**Fig 1:**
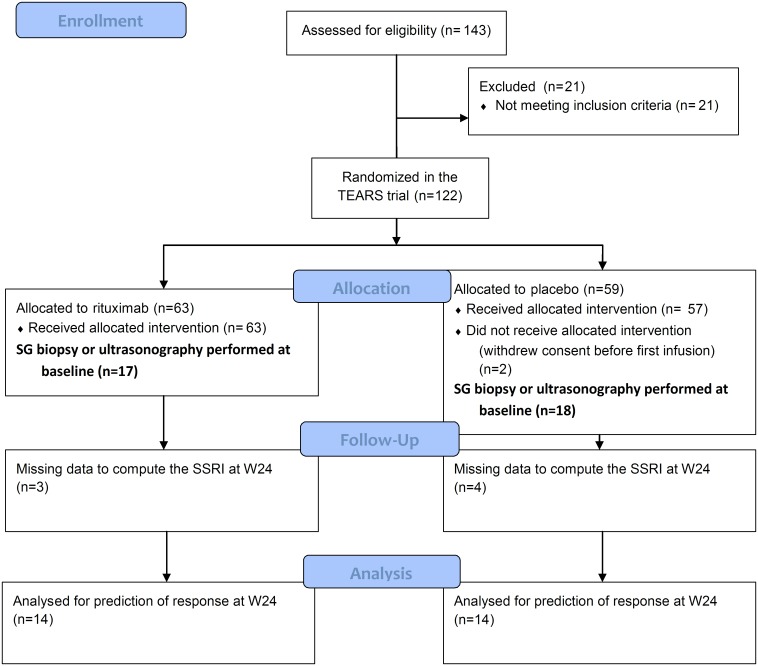
Flowchart of the patients from the TEARS study included in the present analyses. SG, salivary gland. SSRI, Sjögren’s Syndrome Responder Index. SG biopsy and ultrasonography were performed at baseline at the discretion of the evaluating physician, as anticipated in the TEARS trial protocol.

The present study is an ancillary analysis of the TEARS study, focusing on patients who underwent in-depth salivary-gland evaluation (by histology and/or ultrasonography) at baseline (dataset available online as S1 Dataset file). Of these 122 patients, 35 [Table 1 and Fig 1] underwent either a minor salivary gland biopsy [MSGB] evaluated centrally at the Brest Teaching Hospital or salivary-gland ultrasonography [SGUS], and had unstimulated whole salivary flow [UWSF] measurement at baseline. Patients were instructed not to eat, drink or smoke for 90 min before UWSF measurement. All UWSF assessements were performed at the same time of the day for each patient during the clinical visit of the trial. Saliva was collected during 15 min, weighted, and UWSF was expressed as mL/min. MSGB and SGUS were performed at the discretion of the evaluating physician, as anticipated in the TEARS trial protocol, and were not required for patient’s participation in the study. SGUS was performed only in the Brest center, which included 28 patients. Therefore, those procedures were not available for all patients participating in the TEARS trial. These 35 patients form the basis for the present study; 17 were allocated at random to rituximab and 18 to the placebo.

**Table 1:** Groups of patients.

	Total population N = 35*	Rituximab responders N = 7	Rituximab non-responders N = 7	*P* value
Female, n [%]	34 [97]	6 [86]	7 [100]	1
Age, median [IQR]	53 [45–61]	46 [3–55]	52 [40–55]	0.65
Years since diagnosis, median [IQR]	2 [1–5]	1 [0.3–4]	3 [1–7]	0.14
Abnormal Schirmer’s test, n [%]	18 [51]	4 [57]	4 [57]	1
Salivary flow <0.1 mL/min, n [%]	26 [74]	5 [71]	6 [86]	1
Salivary flow [mL/min], median [IQR]	0.09 [0.04–0.14]	0.1 [0.06–0.14]	0.04 [0–0.09]	0.14
Anti-SSA/SSB positivity, n [%]	31 [89]	6 [86]	7 [100]	1
Centralised MSGB, n	31	7	7	
Focus score, median [IQR]	1.3 [0.0–5.0]	0.3 [0.0–1.3]	4.0 [2.7–5.3]	0.02
SGUS performed, n	28	7	5	
Highest single-gland SGUS grade, median [IQR]**	3 [3–4]	3 [2–3]	4 [3–4]	0.01
Total SGUS grade, median [IQR]***	9 [7–12]	9 [6–11]	16 [11–16]	0.04

*17 patients were allocated at random to rituximab and 18 to the placebo. 3 patients had missing data precluding the computation of the Sjögren’s Syndrome Responder Index (SSRI)-30 at W24.

**The single-gland grade can range from 0 to 4.

***The total grade can range from 0 to 16.

IQR, interquartile range; MSGB, minor salivary gland biopsy; SGUS, salivary-gland ultrasonography. Categorical data were compared between responders and non-responders using the chi-square or Fisher’s test as appropriate, and continuous data were compared using the Mann-Whitney test.

### Salivary-gland ultrasonography [SGUS]

All 28 patients included at the Brest Teaching Hospital underwent baseline standardised SGUS by a single investigator [SJJ], who was, as all other investigators involved in the study, blinded to treatment allocation. All procedures were performed using the same ultrasound equipment: iU22 scanner (Philips Medical Systems, Andover, MA, USA), linear probe from 5 to 15 mHz. Both parotid and submandibular glands were evaluated. The echostructure of each gland was graded on a scale of 0 to 4 based on the presence or absence of hypoechoic areas, as previously described [9,10]. Grade 0 indicated a normal homogeneous gland; grade 1, small hypoechoic areas without echogenic bands; grade 2, multiple hypoechoic areas measuring <2 mm, with hyperechoic bands; grade 3, multiple hypoechoic areas measuring 2–6 mm, with hyperechoic bands; and grade 4, multiple hypoechoic areas measuring >6 mm or multiple calcifications with hyperechoic bands. In each patient, both the highest SGUS score of the four glands [range, 0–4] and the four scores [range, 0–16] were considered for the analysis.

### Minor salivary gland biopsy [MSGB]

MSGB was performed routinely at 4 of the 14 participating centres [Brest, Lille, Nantes, and Rouen] at baseline. The biopsy specimens in paraffin blocks and the corresponding slides were sent to the coordinating centre in Brest and read by a single specifically trained pathologist [SC], as previously described [24]. The focus score was computed as the number of aggregates containing >50 lymphocytes per 4 mm² of normal-appearing salivary-gland tissue [25].

### Sjögren’s Syndrome Response Index-30 [SSRI-30]

The SSRI-30 [7] is defined as at least 30% improvements between baseline and 6 months in at least two of five criteria: fatigue VAS, oral dryness VAS, ocular dryness VAS, UWSF, and erythrocyte sedimentation rate [ESR]. Among the 17 rituximab-treated patients included in the present study, 7 achieved the SSRI-30 at W24, 7 did not, and 3 had missing data precluding evaluation of the SSRI-30. Responders and non-responders to rituximab were comparable for demographic data and general characteristics of pSS [Table 1]. Among the 18 patients from the placebo group, only 2 were considered responders at W24, 12 were non-responders, and 4 had missing data precluding evaluation of the SSRI-30.

### Statistical analysis

Data were described as median [interquartile range] for continuous variables and number [percentage] for categorical variables. We computed Spearman’s coefficients to assess potential correlations linking the three methods used to assess the baseline severity of salivary-gland involvement [SGUS, focus score, and UWSF]. We then evaluated whether baseline severity of salivary-gland involvement predicted the response to rituximab after 6 months. We compared patient groups using the Mann-Whitney, chi-square, or Fisher’s test, as appropriate. All analyses were performed using GraphPad Prism software or SPSS v20. Values of *p*<0.05 were considered significant.

## Results

Of the 35 patients, 31 underwent centralised focus-score determination and 28 underwent SGUS; both were performed in 24 patients. UWSF was determined in all patients.

**Fig 2:**
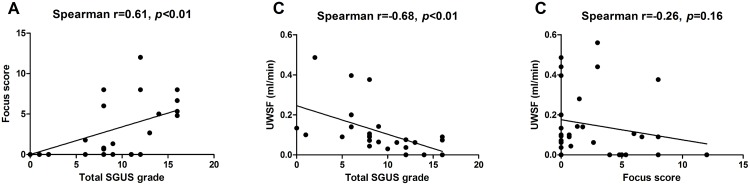
Salivary-gland ultrasonography grade correlates with salivary flow and histology. SGUS, salivary gland ultrasonography; UWSF, unstimulated whole salivary flow. The total SGUS grade obtained by summing the grades of the two parotid and two submandibular glands can range from 0 to 16.

The global SGUS grade correlated positively to the focus score [r = 0.61, *p*<0.01, Fig 2A] and negatively to the UWSF [r = -0.68, *p*<0.001, Fig 2B]. We found no significant correlation between the focus score and UWSF [Fig 2C].

**Fig 3:**
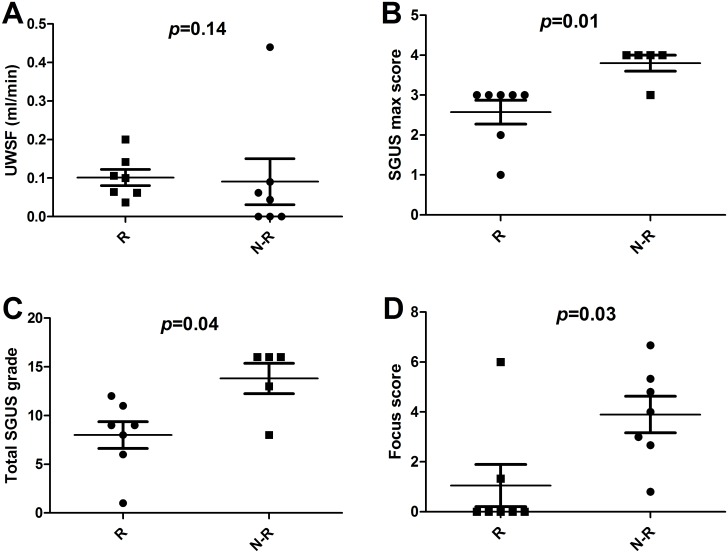
High salivary-gland ultrasonography grade and high focus score predict the response to rituximab. SGUS, salivary gland ultrasonography; UWSF, unstimulated whole salivary flow. The response was assessed using the SSRI-30 (Sjögren’s Syndrome Response Index 30), defined as an at least 30% improvement from baseline to week 24 in at least two of the following five criteria: fatigue visual analogue scale [VAS], oral dryness VAS, ocular dryness VAS, unstimulated whole salivary flow, and erythrocyte sedimentation rate.

In the rituximab group, we observed a trend towards a lower baseline UWSF in non-responders compared to responders [p = NS, Table 1]. However, we were unable to identify a cut-off that reliably separated responders and non-responders [Fig 3A].

The median total SGUS grade at W24 was 9 [6–11] in responders to rituximab versus 16 [11–16] in non-responders [*p* = 0.04, Fig 3B]. None of the 4 patients with the highest SGUS grade in at least one of the four glands achieved the SSRI-30 by W24. Of the 8 patients whose highest single-gland grade was ≤3, 7 achieved the SSRI-30 [Fig 3C].

To better explore how baseline SGUS was associated with improvement after rituximab, we compared median baseline total SGUS grades between patients who experienced or not at least 30% improvement of each individual components of the SSRI (fatigue, oral and ocular dryness VAS, ESR and UWSF) at W24. Median baseline total SGUS grade was lower in patients who experienced a ≥30% improvement from baseline for oral dryness VAS (6 [1.5–8.5] vs 12 [8.5–16], p = 0.04), ocular dryness VAS (2 [1–6] vs 11 [8–16], p = 0.02), and UWSF albeit not significantly (8 [3–10] vs 12 [5–16], p = 0.18), but not for fatigue VAS (9 [4–11] vs 9.5 [5–16], p = 0.83) nor for ESR (11.5 [9–14] vs 8.5 [5–16], p = 0.47).

In the rituximab-treated group, the proportion of patients who achieved the SSRI-30 by W24 was 1/7 [14%] in the group with a focus score >2 versus 6/7 [86%] in the group with a focus score <2. The median baseline focus score was 0.3 [0.0–1.3] in the responders versus 4.0 [2.7–5.3] in the non-responders [*p* = 0.02, Fig 3D].

## Discussion

In our patients with pSS, salivary-gland structure as assessed using SGUS correlated closely with both salivary gland function and histology. Furthermore, the severity of salivary-gland involvement as assessed by SGUS or histology was associated with the response to rituximab after 6 months, whereas UWSF was not.

UWSF has some limitations such as imprecision and variability [26]. However, it is the most widely used test to assess salivary gland functional involvement in pSS and is included in most classification criteria [23]. Several studies have shown that its value progressively decreases with time in patients with recent disease [27], and rituximab was able to slow this decrease over 6 months in the TEARS study [6,7]. Nonetheless, we did not find here that baseline salivary gland functional impairment was clearly associated with response to rituximab.

That rituximab was not effective in patients with the highest SGUS grade or a high focus score at baseline might be ascribable to irreversible salivary-gland damage due to long-standing disease. However, neither age nor disease duration differed significantly between responders and non-responders. Moreover, the patients with high focus scores did not exhibit histological features of irreversible salivary-gland destruction such as fibrosis or adiposis [24]. We have shown here that the focus score and the SGUS score are highly correlated. Thus, the hypoechoic areas detected using SGUS may indicate tissue inflammation rather than tissue damage. Further studies using objective tools to assess tissue fibrosis within salivary glands, such as ultrasonographic elastometry [28–30], would be useful to better understand the significance of these findings, as well as direct comparison of parotid gland biopsy and ultrasonography [31]. Until such studies, comparing directly SGUS findings and histologic analysis of the same glands, are performed, it remains speculative to affirm whether the SGUS hypoechoic areas correspond more to inflammation or to fibrosis/scaring of salivary gland tissue.

Recent publications described the effects of rituximab on salivary-gland infiltrating lymphocyte subsets in pSS patients and their relationship with clinical efficacy. In salivary-gland biopsy samples from the TEARS study and from a previous open-labelled pilot study [32], we assessed the evolution of the proportion of B cells among the lymphocytic infiltrate using an original digital procedure to analyse objectively immunohistochemistry slides [33]. Rituximab infusions induced a drastic decrease of salivary gland B-cell proportion 3 months later, but B cells reappeared within salivary-gland tissue as soon as they were detectable in the peripheral blood [34]. We also observed that patients with the highest B-cell proportion in minor salivary glands at baseline were less likely to be improved by rituximab as defined by the SSRI at 6 months. These observations further suggest that salivary gland hypoechoic lesions, as measured by the SGUS score in the present study, are associated with a higher degree of minor salivary gland inflammation that could impact rituximab efficacy. Interestingly, another study reported that patients with the highest absolute numbers of B cells within the parotid glands were conversely more likely to respond to rituximab as defined by ESSDAI improvement [35], highlighting again the differences between minor and major salivary gland pathology and the need to define consensual response criteria in pSS [36,37].

The 6-month follow-up and the single rituximab course in the TEARS trial may have been too short to assess treatment responses, given the slow pace of progression of pSS. Further rituximab therapy might be effective, however, even in patients with the most severe SGUS morphologic lesions at baseline. Indeed, we previously observed that rituximab induced an improvement of SGUS score in half of the patients of the TEARS study [19]. Specifically, if none of the four patients with baseline severe parotid echostructure abnormality (maximal grade = 4) were considered responders at W24 according to the SSRI, three of them experienced a decrease of maximal SGUS score at 6 months (maximal grade = 3) [19]. We could thus hypothesize that a second rituximab course given at this time would then be able to improve disease activity. This possibility is being assessed in the TRACTISS study in the United-Kingdom (ISRCTN65360827), a large placebo-controlled trial of two rituximab courses in pSS with an assessment of the clinical response after 12 months [38]. Long-term follow-up will be needed in future studies, both in clinical trials and in every-day life cohorts, to define the efficacy of different therapeutic strategies in pSS and to better determine the evolution of SGUS lesions spontaneously and after therapy. This study, despite a small number of patients included, is the first to show that a single rituximab course does not induce a significant clinical response in half of pSS patients, who display the most severe salivary gland involvement. Therefore, SGUS and salivary gland histology could be considered as simple, non-invasive and accessible biomarkers to predict the response to therapy in this difficult-to-treat disease, and help designing inclusion criteria in future trials. New studies, including higher number of patients, different treatment strategies and longer follow-up, are needed to confirm these preliminary observations and validate the clinical utility of SGUS and salivary gland histology as biomarkers.

## Supporting Information

S1 Checklist CONSORT Checklist. (DOC)

S1 Dataset Minimal Dataset. (XLSX)

S1 Protocol English Protocol. (DOCX)

S2 Protocol French Protocol. (PDF)
