# Supplementary material for: High-Grade Salivary-Gland Involvement, Assessed by Histology or Ultrasonography, Is Associated with a Poor Response to a Single Rituximab Course in Primary Sjögren’s Syndrome: Data from the TEARS Randomized Trial
Source: PLoS One. 2016 Sep 23;11(9):e0162787. doi: 10.1371/journal.pone.0162787 (PMC5035078; doi:10.1371/journal.pone.0162787)
Supplement: S1 Protocol — (DOCX) [file pone.0162787.s003.docx]

TEARS Protocol English

HomeFind StudiesSearch ResultsStudy Record Detail
Trial record 2 of 5 for:    saraux
Previous Study | Return to List | Next Study
Tolerance and Efficacy of Rituximab in Sjogren's Disease (TEARS)
This study has been completed.
Sponsor:
University Hospital, Brest
Collaborator:
Ministry of Health, France
Information provided by (Responsible Party):
University Hospital, BrestClinicalTrials.gov Identifier:
NCT00740948
First received: August 22, 2008
Last updated: March 4, 2015
Last verified: March 2015
History of Changes Full Text View Tabular ViewNo Study Results PostedDisclaimerHow to Read a Study Record  Purpose
CLINICAL PHASE II INDICATION Sjogren's syndrome RATIONALE Sjögren's syndrome (SS) is an autoimmune disorder affecting 0.2% to 3% of the general population. Pharmacological treatment can improve the sicca symptoms, often transiently, but they are unable to modify the course of the disease.Open label studies suggested that low-dose rituximab produced acute and complete CD20 depletion in blood and tissue; was well tolerated without corticosteroid use; and significantly improved glandular and extra-glandular manifestations of pSS. Larger controlled studies are now warranted. Our hypothesis is that two infusions of 1000 mg of Rituximab may be better than placebo to treat patients suffering from pSS. To test this hypothesis, we propose to compare patients with recent and/or severe pSS treated with either Rituximab or placebo.

OBJECTIVES Primary objective : Evaluation of the efficacy defined as a 30% improvement between Day 1 and Week 24 in the values on 2 of the 4 VAS measuring global scores of the disease (activity of the disease including extra glandular manifestations), joint pain, fatigue, and the most disturbing dryness.Secondary objectives : Variations from baseline to week 24 of:

The 0-100-mm VAS scores for dry mouth, dry eyes, dry trachea, dry vagina, and dry skin; fatigue; pain; Tender and swollen joint counts; Tender points; Other systemic manifestation; Unstimulated salivary flow rate; Schirmer and van Bijsterveld scores (2-3); C-reactive protein (CRP) and erythrocyte sedimentation rate (ESR); rheumatoid factor (RF); ANA; serum IgG, IgA, and IgM; complement; cryoglobulinemia; and counts of B and T cells; Evaluation of the safety of Rituximab during the study Evaluation of the improvement evaluated on VAS by the physician Evaluation of the disease activity scores as suggested by Bowman and Vitali Evaluation of Chisholm score, B cells characteristics and DNA microarray on labial accessory salivary gland (SG) biopsy samples, and salivary gland echography at inclusion and at week 24.

TRIAL DESIGN Multicenter, randomized, double-blind, placebo-controlled trial NUMBER OF SUBJECTS : 120


Condition  Intervention  Phase 
Sjogren's Disease
 Drug: Rituximab (mabthera) Injection
Drug: Placebo: NaCl 0.9% or Glucose 5%
 Phase 2
Phase 3
 


 
Study Type: Interventional 
Study Design: Allocation: Randomized
Endpoint Classification: Safety/Efficacy Study
Intervention Model: Parallel Assignment
Masking: Double Blind (Subject, Caregiver, Investigator, Outcomes Assessor)
Primary Purpose: Treatment
Official Title: Tolerance and Efficacy of Rituximab in Sjogren's Disease


Resource links provided by NLM:


Genetics Home Reference related topics: Sjögren syndrome
MedlinePlus related topics: Tears
Drug Information available for: Rituximab
U.S. FDA Resources


Further study details as provided by University Hospital, Brest:


Primary Outcome Measures:
•30% improvement between in the values on 2 of the 4 VAS measuring global scores of the disease (activity of the disease), joint pain, fatigue, and dryness. [ Time Frame: 24 weeks ] [ Designated as safety issue: No ]


Secondary Outcome Measures:
•Variations from baseline to week 24 of clinical, biological and histological data [ Time Frame: 24, 36 and 48 weeks ] [ Designated as safety issue: Yes ]


 
Enrollment: 122
Study Start Date: March 2008
Study Completion Date: January 2013
Primary Completion Date: January 2012 (Final data collection date for primary outcome measure)
Arms  Assigned Interventions 
Experimental: 1
Rituximab Drug: Rituximab (mabthera) Injection
2 * 1g of Rituximab at the 1st day and at the 14th day.
Placebo Comparator: 2
Placebo Drug: Placebo: NaCl 0.9% or Glucose 5%
2* 250ml of NaCl 0.9% or Glucose 5% at the 1st day and at the 14th day.


Detailed Description:
TARGET POPULATION Inclusion criteria : Patients will be eligible if :

they fulfill the new American-European Consensus Group criteria for pSS and have :

•a recent (less than 10 years) and active disease as assessed by :
•values > 50 mm on 2 of 4 visual analogue scales (VAS) (0-100mm) that evaluated global scores of the disease (activity of the disease including extra glandular manifestations), pain, sicca syndrome and fatigue over the last week.
•Rheumatoid factor or SSA>1.5N or cryoglobulinemia or hypergammaglobulinemia or high level of beta2 microglobulinemia or hypocomplémentemia.
•and/or at least one of the following severe signs: parotidomegaly, arthritis, purpura, pulmonary involvement, tubulopathy, neurological involvement, thrombocytopenia.
Additional inclusion criteria will be as follows:

•informed consent
•age 18-80 years,
•stable non-steroidal anti-inflammatory drugs
•and no prescription of immunosuppressive agents for at least 4 weeks prior to inclusion
•Use of a reliable mean of contraception (for patients of reproductive potential)
Exclusion criteria :

Patients should be excluded if they have a secondary SS, if they received cytotoxic drugs during the previous 4 months, if they have severe renal or haematological failure, a history of cancer, hepatitis B or C, HIV, tuberculosis, severe diabetes or any other chronic disease or evidence of infection, if they have had severe allergic or anaphylactic reactions to humanized or murine monoclonal antibodies or if they are unable to understand the protocol. Other : neutrophil count < 1.5 x 103/L, live/attenuated vaccine within 28 days prior to baseline, pregnancy, breast feeding,

  Eligibility


 
Ages Eligible for Study:    18 Years to 80 Years
Genders Eligible for Study:    Both
Accepts Healthy Volunteers:    No

Criteria
Inclusion Criteria:

•they fulfill the new American-European Consensus Group criteria for pSS and have :
•a recent (less than 10 years) and active disease as assessed by :
•values > 50 mm on 2 of 4 visual analogue scales (VAS) (0-100mm) that evaluated global scores of the disease (activity of the disease including extra glandular manifestations), pain, sicca syndrome and fatigue over the last week.
•Rheumatoid factor or anti SSA>1.5N or cryoglobulinemia or
•hypergammaglobulinemia or high level of beta2 microglobulinemia or
•hypocomplémentemia.
•and/or at least one of the following severe signs:

◦parotidomegaly,
◦arthritis,
◦purpura,
◦pulmonary involvement,
◦tubulopathy,
◦neurological involvement,
informed consent age 18-80 years, stable non-steroidal anti-inflammatory drugs and no prescription of immunosuppressive agents for at least 4 weeks prior to inclusion Use of a reliable mean of contraception (for patients of reproductive potential)

Exclusion Criteria:

•Patients should be excluded if they have a secondary SS,
•if they received cytotoxic drugs during the previous 4 months,
•if they have severe renal or haematological failure, a history of cancer, hepatitis B or C, HIV, tuberculosis, severe diabetes or any other chronic disease or evidence of infection,
•if they have had severe allergic or anaphylactic reactions to humanized or murine monoclonal antibodies
•or if they are unable to understand the protocol.
•Other : neutrophil count < 1.5 x 103/L, live/attenuated vaccine within 28 days prior to baseline, pregnancy, breast feeding,
  Contacts and Locations

Choosing to participate in a study is an important personal decision. Talk with your doctor and family members or friends about deciding to join a study. To learn more about this study, you or your doctor may contact the study research staff using the Contacts provided below. For general information, see Learn About Clinical Studies.

Please refer to this study by its ClinicalTrials.gov identifier: NCT00740948

Locations
 
France
CHU de Brest 
Brest, France, 29200 
CHU Clermont-Ferrand 
Clermont-ferrand, France, 63003 
GH Le Havre 
Le Havre, France, 76 083 
AP-HP Bicêtre 
Le KREMLIN-BICETRE, France, 94275 
Ch Le Mans 
Le Mans, France, 72 037 
CHRU de LILLE 
Lille, France, 59 037 
CHU de Marseille 
Marseille, France 
Hopital LAPEYRONIE 
Montpellier, France, 34 295 
CHU de Nantes 
Nantes, France, 44 093 
CHU Bichat 
Paris, France, 75018 
Hôpital Cochin APHP 
Paris, France, 75 679 
Hôpital SUD CHU Rennes 
Rennes, France, 35 203 
CHU Rouen 
Rouen, France, 76 031 
CHU de Strasbourg 
Strasbourg, France, 67 200 
Sponsors and Collaborators
University Hospital, Brest
Ministry of Health, France
Investigators
   
Principal Investigator: Alain SARAUX, Pr University Hospital, Brest 

  More Information

Publications:
Devauchelle-Pensec V, Pennec Y, Morvan J, Pers JO, Daridon C, Jousse-Joulin S, Roudaut A, Jamin C, Renaudineau Y, Roué IQ, Cochener B, Youinou P, Saraux A. Improvement of Sjögren's syndrome after two infusions of rituximab (anti-CD20). Arthritis Rheum. 2007 Mar 15;57(2):310-7.

Additional publications automatically indexed to this study by ClinicalTrials.gov Identifier (NCT Number):
Jousse-Joulin S, Devauchelle-Pensec V, Cornec D, Marhadour T, Bressollette L, Gestin S, Pers JO, Nowak E, Saraux A. Brief Report: Ultrasonographic Assessment of Salivary Gland Response to Rituximab in Primary Sjögren's Syndrome. Arthritis Rheumatol. 2015 Jun;67(6):1623-8. doi: 10.1002/art.39088.
Devauchelle-Pensec V, Mariette X, Jousse-Joulin S, Berthelot JM, Perdriger A, Puéchal X, Le Guern V, Sibilia J, Gottenberg JE, Chiche L, Hachulla E, Hatron PY, Goeb V, Hayem G, Morel J, Zarnitsky C, Dubost JJ, Pers JO, Nowak E, Saraux A. Treatment of primary Sjögren syndrome with rituximab: a randomized trial. Ann Intern Med. 2014 Feb 18;160(4):233-42.

 
Responsible Party: University Hospital, Brest
ClinicalTrials.gov Identifier: NCT00740948     History of Changes 
Other Study ID Numbers: TEARS
Study First Received: August 22, 2008
Last Updated: March 4, 2015
Health Authority: France: Agence Nationale de Sécurité du Médicament et des produits de santé
 

Keywords provided by University Hospital, Brest:
 
Sjogren's disease
Rituximab
Treatment
anti CD20
 


Additional relevant MeSH terms:
 
Sjogren's Syndrome
Arthritis
Arthritis, Rheumatoid
Autoimmune Diseases
Connective Tissue Diseases
Dry Eye Syndromes
Eye Diseases
Immune System Diseases
Joint Diseases
Lacrimal Apparatus Diseases
Mouth Diseases
Musculoskeletal Diseases
 Rheumatic Diseases
Salivary Gland Diseases
Stomatognathic Diseases
Xerostomia
Rituximab
Antineoplastic Agents
Antirheumatic Agents
Immunologic Factors
Pharmacologic Actions
Physiological Effects of Drugs
Therapeutic Uses
 


ClinicalTrials.gov processed this record on October 28, 2015
