# Supplementary material for: High-Grade Salivary-Gland Involvement, Assessed by Histology or Ultrasonography, Is Associated with a Poor Response to a Single Rituximab Course in Primary Sjögren’s Syndrome: Data from the TEARS Randomized Trial
Source: PLoS One. 2016 Sep 23;11(9):e0162787. doi: 10.1371/journal.pone.0162787 (PMC5035078; doi:10.1371/journal.pone.0162787)
Supplement: S2 Protocol — (PDF) [file pone.0162787.s004.pdf]

TOLERANCE ET EFFICACITE DU RITUXIMAB DANS LE SYNDROME DE SJOGREN (TEARS STUDY)

Coordinateur

Pr Alain Saraux
Service de Rhumatologie,
CHU de La Cavale Blanche
29609 Brest Cedex, France
Tél. :02 98 34 72 67
Fax :02 98 49 36 27

Méthodologie

Dr Emmanuel Nowak (Statisticien)
Pr Alain Saraux (Brest)

Comité scientifique

Dr Valérie Devauchelle (Brest)
Dr Sandrine Jousse (Brest)
Pr Pierre Youinou (Brest)
Pr Xavier Mariette (Paris KB)
Pr Jacques Eric Gottenberg (Paris KB)
Pr Jean Sibilia (Strasbourg)
Pr Alain Saraux (Brest)

Comité de surveillance des données et effets indésirables

Dr Anne Christine Rat (Clinicienne- Méthodologiste)
Pr Dominique Mottier (Clinicien)
Dr Emmanuel Oger (Méthodologiste)

Investigateurs

Dr Valérie Devauchelle, Dr Sandrine Jousse et Pr Alain Saraux (Brest)
Dr Jean Jacques Dubost (Clermont Ferrand)
Dr Charles Zarnitsky et Dr Didier Alcaix (Le Havre)
Dr Xavier Puechal (Le Mans)
Prs Eric Hachulla et Pierre Yves Hatron (Lille)
Dr Jacques Morel (Montpellier)
Dr Jean-Marie Berthelot et Pr Mohamed Hamidou (Nantes)
Pr Loïc Guillevin, Drs Véronique Le Guern et Christian Pagnoux (Paris Cochin)
Pr Xavier Mariette et Dr Jacques E. Gottenberg (Paris KB)
Pr Aleth Perdriger (Rennes)
Pr Olivier Vittecoq (Rouen)
Pr J Sibilia (Strasbourg)

GUERIR 2007

TOLERANCE ET EFFICACITE DU RITUXIMAB DANS LE SYNDROME DE SJOGREN

Essai n° RB : 07.054

Amendement 1

Version 3.1 du 24 octobre 2007

SHAM n°: 111650

**L'investigateur coordonnateur :**<br><br>Professeur SARAUX<br>Rhumatologie<br>CHU de Brest	Date : ....<br><br>Signature :
**Le Promoteur** :<br>Monsieur Louis Rolland<br>Directeur Général<br>CHU de Brest<br>Avenue Maréchal Foch<br>29609 Brest cedex	Date : ....<br><br>Signature :
**L'investigateur** :	Date : ....<br><br>Signature :

NB : Cette version correspond au texte du protocole et annexes acceptés par le CPP avant envoi aux autorités de santé et autres interlocuteurs de recherche (Investigateurs, directeurs d'hôpitaux,....)
Si par la suite cette version est modifiée, le protocole sera signé une nouvelle fois de toutes les parties.

TABLE DES MATIERES

SYNOPSIS…………………………………………………………………………… 4
RESUME……………………………………………………………………………… 5

1. TERRAIN ET HYPOTHESES ……………………………………………..……….. 6
1.1 TERRAIN………………………………………………………………………..
1.2 HYPOTHESE ……………………………………………………………………
2. OBJECTIFS ……………………………………………………………………….. 7
2.1 OBJECTIF PRINCIPAL…………………………………………………………
2.2 OBJECTIFS SECONDAIRES……………………………………………………
3. POPULATION ETUDIEE …………………………………………………….…. 8
3.1 CRITERES D'INCLUSION………………………………………………………
3.2 CRITERES D'EXCLUSION ………………………………………………………
3.3 NOMBRE DE PATIENTS…………………………………………………………
4. PROTOCOLE DE L'ETUDE………………………………………………………… 10
4.1 DESIGN……………………………………………………………………………
4.2 CRITERES D'EVALUATION……………………………………………………
5. DETAIL DES VISITES ……………………………………………………………… 11
5.1 VISITE D'INCLUSION …………………………………………………………
5.2 SEMAINE 0 ET 1……………………………………………………….........
5.3 SEMAINE 6 ET 16……………………………………………………….......
5.4 SEMAINE 24………………………………………………………………………
5.5 SEMAINE 36 ET 48………………………………………………………………
5.6 RESUME EN TABLEAU……………..……………………………………………
6. TRAITEMENT ………………………………………………………………………… 14
6.1 MEDICAMENT ETUDIE …………………………………………………………
6.2 TRAITEMENT CONCOMITANT …………………………………………………
6.3 TRAITEMENT CONCOMITANT INTERDIT……………………………………..
6.4 PLACEBO……………………………………………………………………………
7. DECLARATION D'EFFETS SECONDAIRES ………………………………………… 18
8. ARRET PREMATURE ……………………………………………………..……..… 22
8.1 SORTIE DE PROTOCOLE……….………………………………………………
8.2 ARRET PREMATURE ……………………………………………………………
9. ANALYSE STATISTIQUE ………………………………………………………… 22
10. CIRCUIT DES PRELEVEMENTS BIOLOGIQUES……………………..……………. 23
11. ASPECTS ETHIQUES………..…………………………..………………….……… 23
12.BUDGET …………………………………………………………………………. 24
13. BIBLIOGRAPHIE…………………………………………………………..………. 24

ANNEXES

ANNEXE A : EVALUATION DU SYNDROME DE SJOGREN ; CRITERES EUROPEENS AMERICAINS ; BIOPSIE ET FLUX SALIVAIRE ; SF-36
ANNEXE B : INFORMATION DES PATIENTS
ANNEXE C : CONSENTEMENT ECLAIRE
ANNEXE D : REPORT DES EFFETS SECONDAIRES
ANNEXE E : CARACTERISTIQUES DU RITUXIMAB
ANNEXE F : CONDUITE A TENIR EN CAS D'ALLERGIE AU PRODUIT
ANNEXE G : GESTION DE LA BASE DE DONNEES
ANNEXE H : CIRCUIT DES PRELEVEMENTS BIOLOGIQUES ET PROCEDURES DE CONSERVATION

SYNOPSIS

**PHASE D'ETUDE :**	III
**METHODE :**	Essai parallèle randomisé en double aveugle, Rituximab versus placebo.
**CRITERES D'INCLUSION** :	Patients ayant un syndrome de Sjögren primitif.
**OBJECTIF PRINCIPAL** :<br>**OBJECTIFS SECONDAIRES** :	Evaluer l'efficacité clinique du Rituximab à 24 semaines.<br>Evaluer la tolérance et le maintien de l'efficacité du traitement pendant 48 semaines.
**TRAITEMENT ETUDIE** :	Rituximab : 2 perfusions d'1 g à 15 j d'intervalle
**NOMBRE DE PATIENTS** :	Il est prévu d'inclure 120 patients.
**DUREE DES INCLUSIONS** :	56 semaines, les patients commençant à être recrutés au cours du mois de novembre 2007
**DUREE DE PARTICIPATION DU PATIENT :** 48 semaines	
**DUREE TOTALE DE L'ETUDE** :	24 mois

RESUME

Rationnel

Le syndrome de Gougerot-Sjögren primitif (SS) est une pathologie chronique caractérisée par une infiltration lymphocytaire et une destruction des glandes salivaires et lacrymales conduisant à une perte des fonctions sécrétoires (sécheresse occulo-buccale). Cette pathologie peut avoir une présentation variée, avec des atteintes systémiques objectives (atteinte neurologique, atteinte vasculaire, atteinte articulaire) ou subjectives (asthénie, douleur).

Actuellement, aucun traitement pharmacologique n'a montré une efficacité sur les symptômes ou sur le cours évolutif de la maladie. Une étude française réalisée avec un anti-TNF (Infliximab) n'a pas permis de montrer sa supériorité par rapport au placebo (1).

Développés à l'origine dans le cadre du traitement des maladies hématologiques tumorales de type B, les anticorps humanisés anti-CD20 permettent de diminuer sélectivement la population de lymphocytes B. De nombreuses études ont suggéré leur efficacité dans les maladies auto-immunes, avec une bonne tolérance (2).

Trois études ouvertes ont été faites dans le SS, qui est l'une des maladies auto-immunes les plus dépendantes des lymphocytes B (3-5). Elles suggèrent une bonne efficacité du produit, surtout dans les formes récentes et/ou avec atteintes viscérales. L'une d'entre elle a été menée à Brest grâce à l'obtention d'un PHRC régional (Brest 2003). Les travaux (6,7) réalisés dans le cadre de cette étude confirment l'efficacité du Rituximab au niveau des glandes salivaires (disparition des lymphocytes B). La description des effets cliniques observés (5) nous a permis de définir le type de patients susceptibles de répondre à un tel traitement.

Objectifs

L'objectif principal de cette étude est d'évaluer l'efficacité du Rituximab en terme d'amélioration en valeur absolue, de plus de 30%, d'au moins deux des 4 échelles visuelles analogiques suivantes : sécheresse, douleur, fatigue et évaluation globale.

Méthode

Nous proposons donc d'initier un essai parallèle randomisé en double aveugle comparant le rituximab à un placebo chez des patients souffrant d'un SS symptomatique. Tous les patients devront avoir un SS soit récent (moins de 10 ans à compter de la première manifestation signalée à un médecin ou constatée par un médecin) et actif, soit avec atteinte viscérale active. Le traitement sera administré aux patients à l'occasion des visites J1 et J15.

Trois unités de traitements seront fournies à la pharmacie de chaque centre et un approvisionnement sera ensuite réalisé par le laboratoire Roche en fonction du rythme d'inclusion du centre. Le rituximab sera adressé au pharmacien qui prépare la poche de perfusion du produit reconstitué. Ce dernier, lors de la randomisation sera informé du traitement administré et délivrera, à l'insu du patient et du médecin, soit du serum physiologique soit du placebo : le placebo sera du sérum physiologique seul, le rituximab étant de même aspect car incolore une fois dilué dans une poche de sérum physiologique. Tous les patients recevront la même prémédication.

Les patients seront évalués à S6, S16, S24 et suivi en ouvert jusqu'à S48. Un suivi en ouvert sera ensuite proposé aux patients ayant eu une réponse au traitement, avec une possibilité de retraitement.

Le projet sera soumis au CPP et un consentement écrit sera demandé aux patients.

Nombre de sujets nécessaires et analyse

Une différence d'au moins 30% du taux de réponse entre le bras actif et le bras placebo de l'étude représenterait une différence cliniquement significative. Un taux de réponse d'environ 30% est estimé sous placebo. Une taille d'échantillon de 49 patients dans

chaque bras permettrait de détecter une telle différence (60% versus 30%) avec une puissance de 80% (erreur de type I=5%, test bilatéral). Afin de tenir compte des éventuels perdus de vue, la taille d'échantillon a donc été fixée à un total de 120 patients

L'analyse sera aussi réalisée en terme de signes objectifs d'atteintes des glandes salivaires et lacrymales, et de signes objectifs d'atteinte viscérale.

La méthode que nous proposons d'utiliser est proche de celle qui a été choisie pour l'évaluation des anti TNF dans cette pathologie (1).

Le taux de réponse attendu est le même dans les deux groupes de patients traités (maladie récente ou maladie avec atteinte extra glandulaire) dans la mesure ou ce sont surtout les trois premières échelles qui s'amélioreront dans le premier cas et les trois dernières dans le second.

La faisabilité parait tout à fait envisageable compte tenu de la motivation des associations de patients d'une part, de la motivation des médecins qui soignent les patients sans succès d'autre part, et de la réussite du travail similaire mené avec l'Infliximab auprès des mêmes centres.

Aucun laboratoire pharmaceutique n'est actuellement intéressé par la réalisation d'étude utilisant un anticorps anti lymphocyte B dans une maladie relativement rare comme le syndrome de Sjogren primitif, mais le laboratoire Roche accepte de fournir le Rituximab nécessaire à la réalisation d'une telle étude.

1. TERRAIN ET HYPOTHESES

1.1 Terrain

Le syndrome de Gougerot-Sjögren

Le syndrome de Gougerot-Sjögren (SS) est une pathologie chronique caractérisée par une infiltration lymphocytaire et une destruction des glandes salivaires et lacrymales conduisant à une perte des fonctions sécrétoires. Il en résulte une xérostomie et une xérophtalmie.

La prévalence du SS est de l'ordre de 0,2 à 1 % (8). Des critères ont récemment été mis au point pour définir l'affection (cf annexe A).

Le SS peut être primaire, lorsqu'il est isolé, ou secondaire lorsqu'on le retrouve associé à une autre maladie auto-immune. Dans ce dernier cas, il s'agit plus souvent d'une polyarthrite rhumatoïde ou d'un lupus érythémateux disséminé. Le SS est habituellement limité aux glandes exocrines. Cependant, chez près d'un tiers des patients, la pathologie est plus systémique pouvant atteindre les vaisseaux, les reins, les poumons, le foie ou le système neurologique (9-11). Cinq pour cent des patients développeront dans le cours de leur maladie un lymphome de type B.

Actuellement, aucun traitement pharmacologique, y compris les corticoïdes et les traitements de fond classiques de maladie auto-immune, n'a montré une efficacité sur les symptômes ou sur le cours évolutif de la maladie.

La physiopathogénie est mal connue mais les cellules B et T sont impliquées (7,12-15).

Rituximab (Anti-CD20) et maladies auto-immunes

Les auto-anticorps sont la marque sérologique des maladies auto-immunes et servent de marqueurs aux perturbations du système immun. On sait que les auto-anticorps ne sont pas eux-mêmes pathogéniques et qu'ils ne sont donc que des simples marqueurs.

Actuellement, on explique la destruction épithéliale par une apoptose prématurée des cellules épithéliales et une cytotoxicité due aux lymphocytes CD4 cytotoxiques.

CD 20 est une phosphoprotéine transmembranaire exprimée à la surface des pré B et des B matures (10). Développés à l'origine dans le cadre du traitement des maladies hématologiques tumorales de type B, les anticorps humanisés anti-CD20 (Rituximab, Roche, UK) permettent de diminuer sélectivement la population de lymphocytes B (16). Ce traitement a été testé comme une nouvelle approche de maladies auto-immunes démontrant par là même l'importance du rôle des lymphocytes B dans l'auto-immunité. De nombreuses études ont démontré l'efficacité du traitement anti-CD20 dans les lymphomes et la polyarthrite rhumatoïde, l'association avec le Méthotrexate ou le cyclophosphamide améliorant les résultats dans la polyarthrite rhumatoïde par comparaison à ceux obtenus avec le Rituximab seul (17-32).

Trois études ouvertes suggèrent l'efficacité du Rituximab dans le SS (3-5). Les études en double aveugle sont maintenant nécessaires. Elles peuvent prendre appui sur les données de ces études ouvertes pour le calcul de la taille d'échantillon. Dans notre étude ouverte, nous avons observé un effet significatif du Rituximab sur la sécheresse, la douleur, la fatigue, les points douloureux, la qualité de vie, et sur l'aspect échographique des glandes salivaires. Les patients qui ont une amélioration très significative (3 des 4 échelles visuelles analogiques) ont une durée d'évolution plus courte que ceux qui n'ont pas atteint ce niveau de réponse (3.8+/-5.4 versus 30.1+/-29.5 ans; $p<0.02$).

La qualité de vie est un des éléments les plus altérés dans le syndrome sec, quelque soit le stade et la présentation (du fait de la fatigue, de la sécheresse, ou de la douleur ou de la présence de signes viscéraux) (33). Elle a été significativement améliorée dans notre étude ouverte (5).

Enfin, la tolérance a été satisfaisante dans les trois études ouvertes (3-5).

2. OBJECTIFS DE L'ETUDE

1-1 Objectif principal

L'objectif principal est de comparer l'efficacité du rituximab à celle d'un placebo, à 24 semaines. L'efficacité est définie par l'amélioration, en valeur absolue, de plus de 30%, d'au moins deux des 4 échelles visuelles analogiques mesurant le score global (activité de la maladie dont les manifestations extra glandulaires), la douleur articulaire, la fatigue, et la sécheresse**.**

1-2 Objectif secondaire

Les objectifs secondaires sont les suivants :

- Etudier la cinétique de la variation des scores de sécheresse, douleur et fatigue au cours de 36 semaines.
- Etudier la cinétique de la variation de signes cliniques : nombre d'articulations gonflées et douloureuses (maximum 64), et appréciation globale du clinicien (échelle visuelle analogique de 100 mm).
- Etudier les modifications échographiques des glandes salivaires avant et après traitement (sous groupe de 3 centres).
- Etudier la cinétique de variation des paramètres biologiques mesurés tout au long de l'étude et incluant vitesse de sédimentation, C réactive protéine, numération formule sanguine, fonction rénale et hépatique, créatine phosphokinase, taux sérique

d'immunoglobulines, anticorps anti-nucléaires et leurs spécificités, facteurs rhumatoïdes, populations lymphocytaires, beta 2 micro globuline et tests centralisés évaluant les conséquences du rituximab sur la fonction lymphocytaire B.
- Etudier la cinétique de variation de l'évaluation objective de la sécheresse buccale (collection de la salive sans stimulation à 5 minutes selon une méthode établie (34) par mesure du poids de la salive sur une balance analytique permettant de déterminer le volume obtenu pour une équivalence d'1 mg = 1 ml), l'évaluation de la sécheresse oculaire (break up time, test de Schirmer, évaluation cornéenne selon le score de Bijsterveld côté de 0 à 9) (35).
- Etudier la variation des paramètres histologiques et l'expression des gènes dans les glandes salivaires grâce à l'utilisation de puces ADN.
- Evaluer la tolérance du Rituximab.
- Evaluer les scores d'activités actuellement à l'étude proposés par des équipes anglaises, italiennes (36) et françaises (non publiés, résultats confidentiels soumis pour publication).

3. POPULATIONS ETUDIEES

3-1 Critères d'inclusion

- Hommes et femmes âgés de 18 à 80 ans.
- Patients remplissant les critères du groupe de consensus européeno américain (AECG) de SS primaire (37).
- Au moment de l'inclusion, tous les patients devront avoir un SS récent et/ou avec atteinte viscérale.
    - Groupe SS récent (premier signe ou symptôme relevé par un médecin datant de moins de 10 ans) : S'il s'agit d'un SS récent, l'activité sera définie comme la présence à l'inclusion d'au moins deux échelles visuelles analogiques sur 4 (activité globale de la maladie, douleur, sécheresse, fatigue au cours de la semaine passée) supérieures à 50/100 et soit un taux élevé d'anticorps anti SSA et/ou facteurs rhumatoïdes (supérieur à 1,5 fois la normale supérieure), soit une cryoglobulinémie (quel que soit son taux), soit une hypergammaglobulinémie (gammaglobuline supérieure>16 g/l à électrophorèse ou IgG > 15g/L en dosage pondéral), soit un taux élevé de bêta 2 micro globuline (au-delà du taux supérieur du laboratoire, en l'absence d'insuffisance rénale), soit une hypocomplémentémie (C4<0,12 g/l). Dans ce cas, les patients seront prévenus de l'avis non unanime des médecins sur le rapport bénéfice risque en raison des risques liés à l'utilisation du Rituximab.
    - Groupe SS systémique actif : S'il s'agit d'un SS de plus de dix ans, comme la sécheresse peut être une séquelle et non un témoin de l'activité de la maladie, les patients ne pourront être inclus que s'ils ont une des manifestations suivantes : parotidomégalie, purpura ou vascularite cutanée, atteinte pulmonaire (pleurésie ou pneumopathie interstitielle confirmée par scanner), atteinte neurologique clinique (telle qu'une neuropathie périphérique confirmée par électromyogramme ou une atteinte centrale confirmée par IRM), atteinte rénale (tubulopathie), cytopénie autoimmune (plaquettes<100 000/mm3, hémoglobine< 10g/100ml, ou neutropénie<1000/mm3), myosite (CPK>2N et/ou EMG et/ou biopsie pathologique), arthrites (≥3), ou lymphadénopathie (au moins 2 adénopathies>1 cm et après exclusion d'une cause tumorale ou infectieuse) ou une autre manifestation systémique sévère après accord écrit du comité scientifique. Les SS ayant l'une de ces atteintes systémiques pourront aussi être inclus dans ce sous groupe, à condition que l'atteinte soit considérée comme étant active par le clinicien, quelque soit la durée d'évolution de la maladie.
- Les hommes et les femmes en âge de procréer devront avoir une contraception efficace (abstinence, contraception orale, stérilet, spermicide ou stérilisation chirurgicale) durant toute la durée de l'étude et devront continuer cette contraception jusqu'à 1 an après la dernière perfusion.

- Traitements concomitants :

. les anti-inflammatoires, l'hydroxychloroquine, le méthotrexate et les corticoïdes pourront être poursuivis à condition qu'ils soient à une dose stable au cours des 4 semaines précédant l'inclusion puis durant le traitement, du moins si la tolérance est satisfaisante. En cas de nécessité, le clinicien pourra modifier le traitement en le notifiant dans le cahier d'observation.

. les traitements immunosuppresseurs (excepté le méthotrexate) devront avoir été arrêtés au moins 4 semaines avant l'inclusion.

. Le chlorydrate de pilocarpine (préparation magistrale, collyre buvable, Salagen®) et la cévimeline (Exovac®) seront autorisés à condition qu'ils soient à une dose stable au cours des 4 semaines précédant l'inclusion puis durant le traitement la encore si la tolérance est satisfaisante. En cas de nécessité, le clinicien pourra modifier le traitement en le notifiant dans le cahier d'observation.

- Les patients devront être capables d'adhérer au protocole d'étude et de l'avoir compris.
- Les patients devront être capables de donner un consentement éclairé signé, ce dernier étant obtenu avant l'inclusion.
- Les patients devront être affiliés à la Sécurité Sociale.

3-2 Critères d'exclusion

- Patient non coopératif et ayant refusé de signer le consentement éclairé.
- Patient incapable de comprendre le protocole.
- Participation à un autre essai thérapeutique.
- Signes cliniques ou biologiques d'autres maladies auto-immunes que le SS.
- Patients ayant déjà été traités par Rituximab.
- Signes et symptômes de pathologie sévère progressive ou non contrôlée au niveau rénal, hépatique, hématologique, endocrinien, pulmonaire, cardiaque, neurologique ou cérébral.
- Allergie ou réaction anaphylactique à un anticorps murin.
- Hommes et femmes en âge de procréer sans contraception au cours de l'étude ou refusant d'en prendre une durant 1 an après la dernière perfusion.
- Femmes enceintes ou allaitantes.
- Patients alcooliques ou toxicomanes.

Patients ayant une dose non stable d'anti-inflammatoire, d'hydroxychloroquine, de méthotrexate, de chlorydrate de pilocarpine (préparation magistrale, collyre buvable, Salagen®) et la cévimeline (Exovac®) au cours des 4 semaines précédant l'inclusion.

- Patients ayant un reçu un agent immunosuppresseur au cours des 4 semaines précédant l'inclusion.
- L'utilisation d'un vaccin vivant atténué dans les 4 semaines précédant l'inclusion ou durant l'étude.
- Hépatite B présente ou passée (Ag HBs et/ou Ac anti HBc positif, Ac anti HBs sans notion de vaccination), hépatite C (Ac anti VHC) et infection VIH.
- Patients devant être vaccinés contre les infections à haemophilus ou pneumocoque et ne l'ayant pas été dans les 28 jours précédant la première perfusion de Rituximab.
- Patients ayant un taux d'immunoglobline IgG, IgA et IgM inférieur à la valeur normale
- Infections sérieuses (telle qu'une hépatite, une pneumonie ou une pyélonéphrite) au cours des 3 mois précédant l'inclusion.
- Affections opportunistes au cours des 3 mois précédant l'inclusion.
- Toute autre infection opportuniste antérieure devra avoir été traitée avec succès et considérée par l'investigateur comme définitivement guérie.
- Les patients à risque infectieux devront être considérés comme éventuellement excluables à la discrétion de l'investigateur.
- Infections documentées.
- Neutropénie inférieure à 1500 par mm3 et d'autre cause qu'autoimmune.
- Intervention chirurgicale prévue lors de la durée de l'étude.

- Cancer au cours des 5 années passées (excepté le carcinome baso-cellulaire ou spino-cellulaire de la peau dont le traitement a été complet et pour lesquels il n'y a pas de risque évident de récidive).
- Antécédent de pathologie hématologique tumorale ou signes et symptômes pouvant suggérer une possible pathologie hématologique tumorale en cours.
- Patients non affiliés à la Sécurité Sociale

3-3 Nombre de patients

La taille d'échantillon a été estimée à partir des données obtenues dans notre étude ouverte (5) et dans l'étude TRIPPS (1).

On considère qu'une différence d'au moins 30% du taux de réponse entre le bras actif et le bras placebo de l'étude représenterait une différence cliniquement significative. Les données de l'étude TRIPPS (1) permettent d'estimer un taux de réponse d'environ 30% sous placebo. Une taille d'échantillon de 49 patients dans chaque bras permettrait de détecter une telle différence (60% versus 30%) avec une puissance de 80% (erreur de type I=5%, test bilatéral). Afin de tenir compte des éventuels perdus de vue, la taille d'échantillon a donc été fixée à un total de 120 patients.

Cette importance de différence attendue entre le placebo et le rituximab est justifiée par la nécessité de claire supériorité du traitement compte tenu de son coût et de la relative lourdeur d'utilisation. Compte tenu des résultats spectaculaires rapportés dans les études ouvertes pour les formes récentes et/ou avec atteinte viscérale, cette différence d'efficacité peut être escomptée.

Douze centres (Brest, Clermont Ferrand, Le Havre, Le Mans, Lille, Montpellier, Nantes, Paris Cochin, Paris KB, Rennes, Rouen, Strasbourg) participeront à l'inclusion des patients et incluront donc en moyenne 10 patients chacun en 36 semaines, soit 1 à 2 patients par mois. Tous les centres n'ayant pas les mêmes possibilités de recrutement, les inclusions seront concurrentielles.

4. PROTOCOLE D'ETUDE

4-1 Design de l'étude

Il s'agit d'un essai parallèle randomisé en double aveugle (Rituximab versus placebo) ayant pour but d'évaluer l'efficacité du Rituximab à 24 semaines.

4-2 Critères d'évaluation

4-2-1 Critère principal

Le critère principal est l'amélioration (définie comme une baisse d'au moins 30 points sur 100) d'au moins deux des 4 échelles mesurant le score global (activité de la maladie dont les manifestations extra articulaires), la douleur articulaire, la fatigue, et la sécheresse à 24 semaines.

Ce choix du critère principal est justifié par les travaux antérieurs qui l'ont utilisés (1, 2), permettant ainsi un calcul de taille d'échantillon. Aucun autre critère n'a actuellement été validé dans cette pathologie.

4-2-2 Critères secondaires

Nous étudierons :

- La cinétique de variation des 4 échelles cliniques, des paramètres cliniques, du nombre d'articulations douloureuses (maximum 64), du score global évalué par le médecin (échelle visuelle analogique 0 à 100 mm), du questionnaire SF 36, des différents critères objectifs d'activité de la maladie. Les résultats seront reportés aux visites suivantes : inclusion, 6 semaines, 16 semaines, 24 semaines, 36 semaines et 48 semaines.
- La modification échographique des glandes salivaires (volume et doppler puissance) sera évaluée entre la visite d'inclusion et celle de la semaine 24 dans les centres qui en ont la possibilité (Brest, Lille, Rennes et Rouen).
- La cinétique de variation des paramètres biologiques (vitesse de sédimentation, C réactive protéine, numération formule sanguine, fonction rénale et hépatique, créatine phosphokinase, taux sérique d'immunoglobulines, anticorps anti-nucléaires et leurs spécificités, facteurs rhumatoïdes, populations lymphocytaires, beta 2 micro globuline et tests centralisés évaluant les conséquences du rituximab sur la fonction lymphocytaire B) sera réalisée aux visites suivantes : inclusion, 6 semaines, 16 semaines, 24 semaines, 36 semaines et 48 semaines.
- La cinétique de variation des critères objectifs de bouche sèche (salive totale non stimulée), de sécheresse oculaire (test de Schirmer) et d'atteinte viscérale seront réalisés aux visites suivantes : inclusion, 6 semaines, 16 semaines, 24 semaines, 36 semaines et 48 semaines. En outre, la sécheresse oculaire sera évaluée par le break-up time, le test de Schirmer, et le test au vert de lissamine à la visite d'inclusion et à S24.
- La variation du score histopathologique et de l'expression des gènes dans les biopsies des glandes salivaires. Une biopsie de glande salivaire sera conseillée à l'inclusion (pour le diagnostic et l'évaluation de l'importance de l'atteinte) puis à 24 semaines (pour l'évaluation de la réponse thérapeutique), dans tous les centres.

5. DETAIL DES VISITES

5-1 J-7 : visite d'inclusion

A la visite d'inclusion, les patients signeront le formulaire de consentement après avoir été informés oralement et par écrit.

Les patients seront informés de la nécessité d'un test de grossesse s'ils sont en âge de procréer et de l'obligation de contraception durant le traitement et l'année qui suit.

Tous les paramètres cliniques, ophtalmologiques, biologiques, la biopsie de glandes salivaires, et le questionnaire SF-36 seront obtenus à cette consultation ; Les patients auront les examens suivants :

- Evaluation des scores cliniques par le patient (échelle visuelle analogique 0 à 100 mm) : score global, douleur, syndrome sec et fatigue.
- Le volume total salivaire sera collecté en 5 minutes (la mesure du poids sur une balance analytique permettra de déterminer le volume salivaire obtenu en estimant qu'1 g = 1 ml).
- Évaluation des scores de sécheresse oculaire (break-up time, le test de Schirmer, et le test au vert de lissamine, examen à la lampe à fente).
- Nombre d'articulations douloureuses (maximum 64).
- Evaluation des atteintes viscérales.
- Evaluation globale par le médecin (sur une échelle visuelle analogique de 100 mm).
- Examen clinique standard.
- Echographie des glandes salivaires dans les centres de Brest, Lille, Rennes et Rouen (facultative).
- Examens biologiques : vitesse de sédimentation, C réactive protéine, numération formule sanguine, fonction rénale, hépatique et pancréatique, créatine phosphokinase, taux sérique d'IgA, IgG, IgM, anticorps anti-nucléaires et leurs spécificités, facteurs rhumatoïdes, populations lymphocytaires, beta 2 micro globuline, complément sérique C4, cryoglobulinémie, et tests centralisés évaluant les conséquences du rituximab sur la fonction lymphocytaire B.

- Une sérologie d'hépatite B (Ac anti HBs et HBc, Antigène HBs), d'hépatite C, d'infection CMV, Haemophilus, Pneumocoque ainsi qu'une sérologie VIH sera réalisée à cette visite.
- Biopsie des glandes salivaires (facultative mais conseillée).
- Questionnaire SF-36.

Cette visite aura lieu entre -30 et -7 jours avant V1.

Entre les deux visites, et au plus tard 4 jours avant V1, le numéro de randomisation sera demandé par l'investigateur des centres, par fax, auprès du CIC du CHU de Brest. Ce dernier répondra à la demande dans les 24 heures aux heures ouvrables et transmettra le numéro de randomisation au pharmacien local, ainsi qu'une information au coordonnateur, au laboratoire Roche et au promoteur pour permettre un décompte du nombre de patient inclus et l'envoie d'unités de traitement dans les centres.

5-2 Jour 1 et Jour 15

Les visites devront être réalisées au jour prévu.
Le traitement sera administré à l'occasion de ces deux visites. Lors de ces 2 semaines, les patients auront un examen clinique et des examens biologiques standard.
Les patients auront les examens suivants :
- évaluation des scores cliniques par le patient (échelle visuelle analogique 0 à 100 mm) : gêne globale, douleur, syndrome sec et fatigue.
- Examen clinique standard.
- Examens biologiques : vitesse de sédimentation, C réactive protéine, numération formule sanguine, fonction rénale et hépatique.

5-3 Semaine 6 et 16

La visite devra être réalisée au jour prévu ± 2 jours.
Les patients auront les examens suivants :
- Evaluation des scores cliniques par le patient (échelle visuelle analogique 0 à 100 mm) : score global, douleur, syndrome sec et fatigue.
- Le volume total salivaire sera collecté en 5 minutes (la mesure du poids sur une balance analytique permettra de déterminer le volume salivaire obtenu en estimant qu'1 g = 1 ml).
- Test de Schirmer.
- Nombre d'articulations douloureuses (maximum 64).
- Evaluation des atteintes viscérales.
- Evaluation globale par le médecin (sur une échelle visuelle analogique de 100 mm).
- Examen clinique standard.
- Examens biologiques : vitesse de sédimentation, C réactive protéine, numération formule sanguine, fonction rénale et hépatique, créatine phosphokinase, taux sérique d'IgA, IgG, IgM, anticorps anti-nucléaires et leurs spécificités, facteurs rhumatoïdes, beta 2 micro globuline, complément sérique C4, cryoglobulinémie.
- Questionnaire SF 36.

5-3 Semaine 24

La visite devra être réalisée au jour prévu ± 2 jours.
Les patients auront les examens suivants :
- Evaluation des scores cliniques par le patient (échelle visuelle analogique 0 à 100 mm) : score global, douleur, syndrome sec et fatigue.
- Le volume total salivaire sera collecté en 5 minutes (la mesure du poids sur une balance analytique permettra de déterminer le volume salivaire obtenu en estimant qu'1 g = 1 ml).

- Évaluation des scores de sécheresse oculaire (break-up time, le test de Schirmer, et le test au vert de lissamine, examen à la lampe à fente).
- des scores de sécheresse oculaire.
- Nombre d'articulations douloureuses (maximum 64).
- Evaluation des atteintes viscérales.
- Evaluation globale par le médecin (sur une échelle visuelle analogique de 100 mm).
- Examen clinique standard.
- Examens biologiques : vitesse de sédimentation, C réactive protéine, numération formule sanguine, fonction rénale et hépatique, créatine phosphokinase, sérologie CMV, Haemophilus et Pneumocoque, taux sérique d'IgA, IgG, IgM, anticorps anti-nucléaires et leurs spécificités, facteurs rhumatoïdes, beta 2 micro globuline, complément sérique C4, cryoglobulinémie et tests centralisés évaluant les conséquences du rituximab sur la fonction lymphocytaire B.
- Une biopsie des glandes salivaires et une échographie seront réalisées à la semaines 24 (facultative mais conseillée).
- Questionnaire SF 36.

5-5 Semaine 36 et 48

Les visites seront réalisées en ouvert, avec les examens cliniques et biologiques suivants :
- Evaluation des scores cliniques par le patient (échelle visuelle analogique 0 à 100 mm) : score global, douleur, syndrome sec et fatigue.
- Examen clinique standard.
- Examens biologiques : vitesse de sédimentation, C réactive protéine, numération formule sanguine, fonction rénale et hépatique.
- Nombre d'articulations douloureuses (maximum 64).
- Evaluation des atteintes viscérales.
- Le volume total salivaire sera collecté en 5 minutes (la mesure du poids sur une balance analytique permettra de déterminer le volume salivaire obtenu en estimant qu'1 g = 1 ml).
- Test de Shirmer.
- Evaluation globale par le médecin (sur une échelle visuelle analogique de 100 mm).
- Examen clinique standard.
- Examens biologiques : vitesse de sédimentation, C réactive protéine, numération formule sanguine, fonction rénale et hépatique, créatine phosphokinase, taux sérique d'IgA, IgG, IgM, anticorps anti-nucléaires et leurs spécificités, facteurs rhumatoïdes, beta 2 micro globuline, complément sérique C4, cryoglobulinémie et pour la visite S48, sous populations lymphocytaires de tous les patients qui seront centralisées et étudiées à Brest.
- Questionnaire SF-36.

5-5 Post étude

Après S48, **un suivi post étude sera proposé** pour rechercher de rares effets secondaires tardifs et juger de l'efficacité à long terme, avec une consultation tous les 6 mois pendant 18 mois. Les examens réalisés seront les mêmes qu'aux visites S36 et S48.

5-6 Retraitement

A partir de S36, en cas de réponse au traitement (amélioration de 30%) puis de rechute, deux nouvelles perfusions seront éventuellement proposée lors des visites de suivi: Deux perfusions à 15 jours d'intervalle de Rituximab ou de placebo (le même traitement que lors de la première cure) seront proposées dès lors que la perfusion précédente aura été faite plus de 6 mois auparavant. Ce retraitement pourra être fait une seule fois chez tous les patients qui auront été améliorés et qui le souhaitent jusqu'à la levée

de l'aveugle, c'est-à-dire 24 semaines après la première perfusion du dernier patient inclus dans l'étude.

Néanmoins, le retraitement ne sera envisagé que si les patients ont un taux d'immunoglobline IgG, IgA et IgM au moins égal à la moitié de la valeur normale (cf chapitre 6.1 retraitement) et que la sérologie CMV n'a pas doublé entre l'inclusion et la semaine 24.
Si les patients ont été vaccinés contre haemophilus et/ou pneumocoque avant le traitement, il sera conseillé à l'investigateur de vérifier la sérologie à la semaine 24.

5-7 Résumé sous forme de tableau

Perfusions de Rituximab et suivi

	inclusion V0	V 1	V 2	V 3	V 4	Visite finale	Suivi ouvert	Suivi ouvert	Post étude
**Date**	**J-30 à J-7**	**J1**	**J15**	**S6**	**S16**	**S24**	**S36**	**S48**	**Tous les 6 mois**
Consentement	+								
Perfusion du médicament de l'étude		+	+	-	-	-	+/-**	+/-**	+/-**
Evaluation clinique subjective	+	+	+	+	+	+	+*	+	+/-
Evaluation objective	+	-	-	+*	+*	+	+*	+*	+/-
Biopsie de glande salivaire (conseillées) et échographie (Brest Lille Rennes Rouen)	+	-	-	-	-	+	-	-	-
Biologie	+	+	+	+	+	+	+	+	+/-
Immunologie et CPK	+	-	-	+	+	+	+	+	+/-
SF 36	+	-	-	+	+	+	+	+	+/-
Tolérance	+	+	+	+	+	+	+	+	+/-

* : l'évaluation ophtalmologique sera limitée au test de Shirmer
** A partir de S36 les patients pourront être retraités (une seule fois) en cas d'amélioration puis de récidive

6.TRAITEMENT

6.1 Traitements étudiés

Nous avons reporté les principales caractéristiques des produits en annexe E (Vidal 2006).

En bref, le Mabthera (Rituximab) est un anticorps monoclonal chimérique murin/humain obtenu par génie génétique ; il s'agit d'une immunoglobuline glycosylée associant d'une part les régions constantes d'une IgG humaine et d'autres parties régions variables des chaînes légères et lourdes d'origine murine.

Posologie et mode d'administration

La solution de Mabthera préparée doit être administrée en perfusion intraveineuse par une voie spécifique. Les perfusions de Mabthera doivent être administrées en milieu hospitalier avec tous les moyens de réanimation immédiatement disponibles et sous contrôle clinique.

En cas d'effets secondaire, la perfusion ne doit pas être reprise avant disparition complète de tous les symptômes et normalisation des résultats biologiques et clichés pulmonaires. La perfusion peut alors être reprise à une vitesse réduite par rapport à la vitesse initiale. Si des effets indésirables graves surviennent de nouveau, l'arrêt du traitement doit être sérieusement envisagé au cas par cas.

Les réactions légères ou modérées liées à la perfusion (cf Effets indésirables) répondent habituellement à une réduction de la vitesse de la perfusion. Celle-ci peut être augmentée en fonction de l'amélioration des symptômes.

La posologie de* Rituximab *proposée est ici de 1000 mg administrés en perfusion intraveineuse à J1 puis à J15.

Première perfusion: Il est recommandé de débuter la perfusion à une vitesse de 50 mg/h; après les 30 premières minutes, la vitesse de la perfusion pourra être augmentée par paliers de 50 mg/h toutes les 30 minutes, jusqu'à un maximum de 400 mg/h.
Perfusions ultérieures : Lors des perfusions ultérieures de Rituximab, la vitesse initiale pourra être de 100 mg/h, puis augmentée de 100 mg/h toutes les 30 minutes jusqu'à un maximum de 400 mg/h.
Ajustement posologique pendant le traitement : Aucune réduction de la dose de Rituximab n'est recommandée.

Retraitement: A partir de S36, en cas de réponse au traitement (amélioration de 30%) puis de rechute, deux nouvelles perfusions seront éventuellement proposée lors des visites de suivi: Deux perfusions à 15 jours d'intervalle de Rituximab ou de placebo (le même traitement que lors de la première cure) seront proposées dès lors que la perfusion précédente aura été faite plus de 6 mois auparavant. Ce retraitement pourra être fait une seule fois chez tous les patients qui auront été améliorés et qui le souhaitent jusqu'à la levée de l'aveugle, c'est-à-dire 24 semaines après la première perfusion du dernier patient inclus dans l'étude. Néanmoins, le retraitement ne sera envisagé que si les patients ont un taux d'immunoglobline IgG, IgA et IgM au moins égal à la moitié de la valeur normale.

En effet, habituellement, les taux d'IgG et A sont stables mais les IgM baissent de l'ordre de 30%. Le taux d'IgM baisse (cf tableau ci-dessous) sans corrélation avec un risque accru d'infection (38). Les hypogammaglobulinémies importantes sont rares (parfois observées chez l'enfant et en cas d'immunosuppression associée). Mais par précautions, nous ne retraiterons pas les patients ayant une diminution d'une des trois des immunoglobulines en dessous de 50% de la norme.

Evaluation à 24 semaines	***1er traitement (n = 958)***	***2ème traitement (n = 373)***	***3ème traitement (n = 69)***	***4ème traitement (n = 11)***
Ig totales (< N)	***1 (0.1%)***	***2 (0.7%)***	***0***	***0***
IgG (< N)	***13 (1.4%)***	***16 (4.3%)***	***4 (5.9%)***	***0***
IgA (< N)	***7 (0.8%)***	***2 (0.5%)***	***1 (1.5%)***	***0***
IgM (< N)	***93 (10.3%)***	***69 (18.5%)***	***16 (23.5%)***	***6 (54.5%)***

Par ailleurs, le retraitement ne sera envisagé que si la sérologie CMV n'a pas doublé entre l'inclusion et la semaine 24.

Prémédication : Tous les patients recevront 100 mg de methylprednisolone par voie intraveineuse lente, du paracétamol (1 g intra veineux ou per os) et un anti-histaminique (50 mg p.o. diphenhydramine HCL ou i.v équivalent) 30 à 60 minutes avant chaque perfusion de Rituximab.

Precautions d'emploi

-Outre les syndromes de relargages cytokiniques propres aux pathologies tumorales, l'administration intraveineuse de protéines peut provoquer des réactions anaphylactoïdes ou autres réactions d'hypersensibilité. En opposition au syndrome de relargage de cytokines, une réelle hypersensibilité apparaît habituellement dans les minutes qui suivent le début de la perfusion. Les médicaments nécessaires par exemple adrénaline, antihistaminiques et corticoïdes, doivent être disponibles pour un usage immédiat dans l'éventualité d'une réaction allergique pendant la perfusion de Mabthera.

Les manifestations cliniques de l'anaphylaxie peuvent sembler similaires aux manifestations cliniques du syndrome de relargage de cytokines (décrit ci-dessus). Les réactions attribuées à l'hypersensibilité ont été rapportées moins fréquemment que celles attribuées au relargage de cytokines.

-L'éventualité d'une hypotension pendant la perfusion de Mabthera doit faire envisager l'arrêt du traitement antihypertenseur 12 heures avant la perfusion.

- Des cas d'angine de poitrine ou d'arythmie tels que flutter et fibrillations auriculaires, d'insuffisance cardiaque ou d'infarctus du myocarde ont été observés chez des patients traités par Mabthera. En conséquence, les patients présentant des antécédents de pathologies cardiaques et/ou ayant reçu une chimiothérapie cardiotoxique devront être étroitement surveillés.

-Bien que Mabthera ne soit pas myélosuppressif, il est recommandé d'être prudent quant au traitement de patients ayant un nombre de neutrophiles < 1500/mm3 et/ou un nombre de plaquettes < 75 xi 09/i, car l'expérience clinique dans cette population est limitée. Mabthera a été utilisé sans induire de myélotoxicité chez 21 patients qui avaient eu une autogreffe de moelle et chez d'autres groupes à risque avec des réserves médullaires vraisemblablement réduites.

- Lors du traitement par Mabthera en mono thérapie, une surveillance régulière de la numération-formule sanguine, incluant une numération plaquettaire, doit être envisagée. - La solution diluée pour perfusion ne doit pas être injectée rapidement ni en bolus.

Interactions

On ne dispose encore d'aucune donnée sur les interactions éventuelles de Mabthera avec d'autres médicaments. Cependant, chez les patients ayant des taux décelables d'anticorps anti-murins ou d'anticorps humains anti-chimères (HAMA/HACA), l'utilisation d'autres anticorps monoclonaux à des fins diagnostiques ou thérapeutiques peut provoquer des réactions d'allergie ou d'hypersensibilité.

Leucoencéphalite multifocale progressive

Dans le cadre de lupus et de vascularite, quelques cas de leucoencéphalite multifocale progressive d'évolution fatale, ont été décrits. En cas de signes neurologiques, ce diagnostic doit être évoqué et le patient doit être orienté auprès d'un neurologue et avoir, notamment, une imagerie par résonnance magnétique (IRM) et une ponction lombaire.

6.2 Traitements associés

Traitements du syndrome de Sjögren primitif associés autorisés

- Traitements locaux substitutifs : Leur utilisation (dose et rythme) est laissée à l'appréciation de chaque patient, elle sera consignée dans le cahier d'observation.
- Les corticoïdes à dose stable.
- Les traitements de fond par hydroxychloroquine utilisés à dose stable dans les 3 mois précédant l'inclusion et pendant la durée de l'essai.
- Les anti-inflammatoires non stéroïdiens et les antalgiques à dose stable. En cas d'amélioration clinique importante constatée, l'investigateur pourra baisser leur dose, en le consignant dans le cahier d'observation.
- Les inhibiteurs de la recapture de la sérotonine, les benzodiazépines et apparentés utilisés à dose stable dans le mois précédent l'inclusion et pendant la durée de l'essai.

Autres traitements autorisés

Tous les traitements non précisés dans la liste des traitements interdits.

Traitements associés interdits

- Corticothérapie locale intra-articulaire ou canalaire. Une corticothérapie per os différente de celle autorisée (dose stable) ou par voie injectable ne devra pas être utilisée, sauf en cas de nécessité médicale (notamment en cas d'allergie). Le maintien du patient dans le protocole sera discuté alors au cas par cas par le comité scientifique.
- Thalidomide, ou autre modulateur du TNF alpha.
- Le chlorydrate de pilocarpine (préparation magistrale, collyre buvable, Salagen®) et la cévimeline (Exovac®) à dose non stable.
- La ciclosporine en collyre.
- L'endoxan et le chloraminophène.

6.3 Traitements pour la prise en charge des réactions immédiates survenant pendant ou deux heures après la perfusion (cf chapitre Mabthera)

Face à l'apparition d'une réaction immédiate durant la perfusion, la première attitude est de ralentir le débit. Si cette manœuvre ne suffit pas à juguler les signes, la perfusion doit être suspendue et si les effets persistent, ils peuvent nécessiter un traitement symptomatique et une réévaluation de la poursuite de la perfusion doit être faite. Un équipement et des médicaments d'urgence pour le traitement de ces effets sont gardés à proximité durant le traitement et pendant les deux heures suivantes.

6.4 Distribution des unités de traitement et de Placebo

L'étude est faite en double aveugle, le médecin et le patient ne sachant pas si le patient reçoit un placebo ou du Rituximab.

Trois unités de traitement seront fournies à chaque centre et un approvisionnement sera ensuite réalisé par le laboratoire Roche en fonction du rythme d'inclusion du centre. Le Rituximab sera adressé au pharmacien qui prépare la poche de perfusion du produit reconstitué. Ce dernier, lors de la randomisation sera informé du traitement administré et délivrera soit du rituximab dilué dans du serum physiologique soit du serum physiologique seul (placebo) : le placebo sera donc du sérum physiologique seul, le Rituximab étant de même aspect car incolore une fois dilué dans une poche de sérum physiologique. Le volume apparent sera le même, et la présentation identique.

Tous les patients recevront la même prémédication.

Le pharmacien saura localement s'il s'agit de traitement actif ou de placebo mais ni le patient, ni le médecin, ni le laboratoire Roche ne le sauront.

6.5 Randomisation

Entre la visite d'inclusion et au plus tard 4 jours avant V1, le numéro de randomisation sera demandé par les investigateurs des centres, par fax, auprès du CIC du CHU de Brest.
La randomisation sera créée de façon manuelle (non électronique) et transmise par fax. Il s'agit d'une randomisation par centres et par blocs de tailles aléatoires.

Le CIC de Brest répondra à la demande dans les 24 heures aux heures ouvrables et transmettra le numéro de randomisation au pharmacien local, ainsi qu'une information sur l'inclusion (mais pas sur le type de traitement) au coordonnateur, au laboratoire Roche et au promoteur pour permettre un décompte du nombre de patient inclus et l'envoi d'unités de traitement dans les centres.

7. REPORT DES EFFETS SECONDAIRES

7.1 Evénements Indésirables et Anomalies Biologiques

Un évènement indésirable est défini comme étant :

Toute manifestation nocive non recherchée, subie par une personne participant à une recherche biomédicale, quelle que soit la cause de cette manifestation (recommandation du 12/09/94 - Afssaps).

En conséquence, tout signe imprévu et défavorable (y compris, par exemple un résultat de laboratoire anormal), tout symptôme, ou toute pathologie temporairement associée à l'usage du produit médicamenteux, qu'il soit ou non considéré comme lié au produit médicamenteux, peut constituer un événement indésirable (Adverse Event – AE).

Des conditions de base (préexistantes à l'essai) qui s'aggraveraient pendant un essai clinique, doivent être rapportées en tant qu'événement indésirables.

L'intensité des événements indésirables peut être cotée sur l'échelle à 4 points de l'O.M.S. (voir W.H.O. Handbook for Reporting Results of Cancer Treatment).

- Intensité légère (grade 1) : inconfort notable mais pas d'entrave aux activités quotidiennes
- Intensité modérée (grade 2) : inconfort suffisant pour réduire ou affecter les activités quotidiennes
- Intensité sévère (grade 3) : impossibilité de travailler ou de réaliser les activités quotidiennes
- Intensité mettant en jeu la vie du patient (grade 4) : représentant une menace immédiate pour la vie du patient.

Le lien de causalité entre le traitement/la recherche et l'événement indésirable doit toujours être évalué par l'investigateur.

L'investigateur détermine la relation de causalité dans la survenue de l'événement, des produits à l'essai et/ou des procédures du protocole. Elle peut être définie comme :

- « reliée »,
- « non reliée »,

en fonction de causes extérieures (état clinique du patient, maladie concomittante) de la chronologie de l'événement (association raisonnable temporelle avec l'administration du medicament, disparition à l'arrêt du medicament, réapparition à sa reintroduction).

Evènement indésirable non grave

Tout évènement indésirable non grave doit devra être notifié dans le cahier d'observation dans la section prévue à cet effet. L'évènement peut correspondre à un symptôme, un diagnostic ou un résultat d'examen complémentaire jugé significatif. Tous les éléments cliniques ou paracliniques permettant de décrire au mieux l'évènement correspondant doit être reportés.

Tout patient présentant un évènement doit être suivi jusqu'à la résolution ou la stabilisation de celui-ci, et l'évolution sera notée dans le cahier d'observation.

Un Evénement Indésirable Grave (EIG) [Serious Adverse Event (SAE)] est un événement indésirable qui, quelle que soit la dose de médicament administrée :

· entraîne le décès (note: le décès est une évolution et non pas un événement)

· met en danger la vie du patient (note : l'expression est réservée à une menace vitale immédiate au moment de l'événement indésirable),

· nécessite une hospitalisation ou la prolongation d'une hospitalisation,

· provoque une incapacité ou un handicap important ou durable,

· se traduit par une anomalie ou une malformation congénitale,

· est médicalement significative ou nécessite une intervention afin de prévenir la survenue d'une ou plusieurs des évolutions listées ci-dessus.

Evènements inattendus

Est considéré comme évènement inattendu tout évènement non mentionné ou différent de part sa nature, son intensité ou sa fréquence par rapport à la "brochure investigateur" ou au résumé des caractéristiques du produit pour les médicaments ayant une autorisation de mise sur le marché.

Toutes les exigences de la Guideline ICH pour la Gestion des Données de Tolérance Clinique, intitulée « Clinical Safety Data Management, Definitions and Standards for Expedited Reporting, Topic E2 » seront respectées.

Dans le cadre de cette étude, les événements suivants ne seront pas considérés comme des événements indésirables graves :

· Les hospitalisations ou procédures chirurgicales résultant de l'état initial du patient et n'ayant pas été aggravés depuis la prise du traitement à l'essai. Cela inclus, par exemple, les cholécystectomies suites à des calculs biliaires, les prothèses articulaires, les tests diagnostics. Ces événements devront néanmoins être reportés en tant que procédures médicales dans le CRF.

· Les hospitalisations pour l'administration du traitement à l'essai sauf si ces hospitalisations durent plus de 24 heures.

Les Evénements Indésirables Graves reliés DOIVENT être collectés et rapportés quelque soit le délai écoulé depuis la dernière prise du traitement à l'étude, même si l'étude a été clôturée.

Les Evénements Indésirables Graves non reliés doivent être collectés et rapportés pendant l'étude et jusqu'à 24 semaines après la dernière prise du traitement de l'étude.

Les EI, en particulier ceux pour lesquels le lien de causalité avec le médicament à l'étude n'est pas "non relié", doivent être suivis jusqu'à ce qu'ils soient revenus à l'état basal ou soient stabilisés. Si une explication claire est établie, elle doit être enregistrée dans le Cahier d'Observation.

Les résultats des analyses biologiques seront enregistrés dans le Cahier d'Observation, ou apparaîtront sur les rapports biologiques électroniques soumis directement par le laboratoire central, si applicable.

Tout résultat biologique anormal remplissant les critères d'un évènement indésirable grave (EIG) sera rapporté comme tel, en plus d'être déclaré comme EI dans le Cahier d'Observation.

Tout résultat biologique anormal résultant du traitement étant cliniquement significatif, c'est à dire, rencontrant une ou plusieurs des conditions suivantes, doit être enregistré en tant que diagnostic unique sur la page EI du Cahier d'Observation :

- est accompagné de symptômes cliniques
- entraîne une modification du traitement à l'étude (par exemple, modification de dose, arrêt momentané ou définitif)
- nécessite un changement des traitements concomitants (par exemple addition, arrêt momentané ou définitif, ou toute autre modification d'un traitement ou d'une thérapie concomitant(e)).

Tous les EIG devront faire l'objet d'un rapport sur un formulaire type « Evènement Indésirable Grave » en accord avec les bonnes pratiques cliniques. L'investigateur devra informer par téléphone ou par fax le promoteur (Délégation à la Recherche Clinique et à l'Innovation du CHU de Brest – fax : 02-98-22-31-83) dans les 24 heures suivant la constatation. L'investigateur doit remplir la page prévue dans le cahier d'observation pour les évènements intercurrents, et le formulaire "rapport d'évènement grave survenant au cours d'essais cliniques" annexé au cahier d'observation. Les 2 premières pages de ce formulaire devront être retournées au promoteur de l'essai dans les 48h suivant l'appel initial. Les bilans cliniques, les examens diagnostiques et les examens de laboratoire appropriés seront réalisés afin de déterminer l'origine de la réaction et les résultats de ces explorations ainsi que l'évolution du trouble seront rapportés. Parallèlement au formulaire, l'investigateur préparera si nécessaire un rapport écrit sur cet évènement grave et sur les conséquences qui en découlent

7.2 Gestion des Paramètres de Tolérance

Tout événement indésirable survenu pendant l'étude clinique doit être rapporté sur la page événement indésirable du Cahier d'Observation.

En cas de valeurs biologiques anormales non expliquées et médicalement significatives, les tests doivent être répétés et suivis jusqu'à ce qu'ils soient retournés à la normale et/ou qu'une explication adéquate de l'anomalie soit trouvée. Si une explication claire est établie, elle doit être enregistrée dans le Cahier d'Observation.

Tout événement indésirable clinique ou valeur biologique anormale *grave* survenant pendant le déroulement de l'étude (comme défini dans la section ci-dessus), quelque soit le groupe de traitement, doit être notifié(e) dans le délai d'un jour ouvré à partir du moment où l'investigateur a été mis au courant de l'événement (notification immédiate).

Les exigences concernant la définition et la notification décrites dans la Guideline ICH pour la Gestion des Données de Tolérance Clinique, intitulée « Clinical Safety Data Management, Definitions and Standards for Expedited Reporting, Topic E2 » seront respectées.

Les patientes doivent éviter la survenue d'une grossesse pendant toute la durée de l'étude et aussi longtemps qu'il existe une déplétion en lymphocytes B CD20+ périphériques. Aussi, les patientes doivent utiliser une méthode de contraception adéquate pendant toute la durée de l'étude et jusqu'à 1 an après la dernière perfusion de rituximab. Des tests de grossesse seront réalisés régulièrement : test plasmatique à la sélection (screening) et tests urinaires jusqu'à la fin de l'étude.

De même, il est fortement recommandé aux patients de sexe masculin d'utiliser une méthode efficace de contraception pendant toute la durée du traitement par rituximab, jusqu'à 1 an après la dernière perfusion de rituximab.

En cas de grossesse survenant pendant l'étude (y compris pendant le suivi de tolérance), la patiente devra immédiatement informer l'investigateur. La patiente ne recevra plus aucune perfusion de rituximab.

L'investigateur devra conseiller la patiente, discuter des risques de la poursuite de la grossesse et des effets possibles sur le fœtus. La patiente devra être suivie jusqu'au terme de sa grossesse.

L'investigateur devra informer le promoteur dans les 24 heures après avoir été informé.

Les grossesses survenant jusqu'à 90 jours après la dernière prise du traitement de l'étude doivent aussi être rapportées à l'investigateur.

Toute grossesse survenant chez la partenaire d'un patient participant à l'étude doit être notifiée à l'investigateur. L'investigateur doit déclarer toutes les grossesses dans les 24 heures après en avoir été informé.

7.3 Mises en Garde et Précautions

· Réaction aiguë lors de la perfusion, le plus souvent faible à modérée, survenant entre 30 minutes et 2 heures après le début de la 1ère perfusion.

· Les patients ayant des antécédents d'asthme doivent être étroitement suivis pendant les perfusions. En effet, un cas de patient asthmatique bien contrôlé ayant présenté une crise aiguë d'asthme après sa 1ère perfusion de rituximab a été rapporté.

· Une hypotension transitoire peut apparaître pendant la perfusion de rituximab : l'investigateur doit envisager de suspendre le traitement anti-hypertenseur du patient, 12 heures avant la perfusion de rituximab.

· Déplétion prolongée en lymphocytes B CD20+ périphériques :

· Cette déplétion n'est pas associée à une augmentation des infections opportunistes ; néanmoins, il est possible que le risque infectieux soit un peu augmenté. Le rituximab ne doit donc pas être administré en cas d'infection active, et son utilisation doit être

envisagée avec précaution chez les patients ayant des antécédents d'infections chroniques ou récurrentes ou des facteurs de risque pouvant prédisposer aux infections.

· De très rares cas de réactivation d'une hépatite B ont été rapportés chez des patients traités par rituximab en association à une chimiothérapie (CHOP) pour un lymphome non hodgkinien.

· Rarement, des arythmies cardiaques, thrombopénies, neutropénies, anémies et décès ont été associés à l'administration de rituximab à des patients traités en oncologie.

· Les patients avec des antécédents cardiaques (angor, arythmie, insuffisance cardiaque congestive…) doivent être suivis étroitement.

· Les patients doivent également être informés des risques liés à la prise de corticoïdes. Aucune donnée disponible au moment de l'approbation de ce protocole n'indiquait que des mises en garde ou précautions spéciales étaient nécessaires, en dehors de celles notées dans la brochure investigateur.

8. ARRET PREMATURE

8-1 Sortie de protocole

Les patients peuvent sortir du protocole pour différentes raisons, à leur demande, sans raison précise mais aussi, sur avis du conseil scientifique :
- à la demande de l'investigateur.
- en cas d'effets secondaires.
- en cas d'inefficacité.
- en cas de violation de protocole.
- pour raison administrative.
- en cas d'administration d'un traitement interdit tel que défini dans les critères d'exclusion ; si cela est nécessaire, l'investigateur pourra contacter l'investigateur coordinateur.

Pour chaque patient sorti, un examen complet final devra être réalisé pour obtenir l'état de santé au moment de la sortie. La raison de la sortie de protocole devra être notée sur le dossier du patient et sur les fiches prévues à cet effet.
Les patients sortis d'essai ne seront pas remplacés si les injections ont été réalisées. Dans le cas contraire, des patients supplémentaires seront inclus pour les remplacer.

8-2 Arrêt prématuré de l'étude

Le coordonnateur en accord avec le promoteur a le droit d'arrêter l'étude à n'importe quel moment pour des raisons médicales ou administratives. Autant que possible, cet arrêt sera fait après une consultation mutuelle. Les raisons d'arrêt de l'étude seront documentées et les informations seront adressées à un comité de protection des personnes (CCP), et à l'AFFSAPS (unité d'essai clinique).

En cas d'effet secondaire grave, le coordonnateur réunira le comité de surveillance pour prendre les décisions adéquates en terme d'arrêt de l'essai pour des raisons de sécurité.

9. STATISTIQUE

L'analyse statistique sera réalisée en intention de traiter et portera sur tous les patients inclus et évaluables.

9-1 Critère principal d'évaluation

Un patient sera considéré comme répondeur s'il présente des améliorations (baisse d'au moins 30 points) sur au moins 2 des 4 échelles visuelles analogiques mesurant le score global (activité de la maladie dont les manifestations extra articulaires), la douleur articulaire, la fatigue, et la sécheresse à 24 semaines. Le taux de réponse sera alors comparé dans les deux bras de l'étude par un test du Chi2. Les valeurs de p (bilatérales) inférieures à 0.05 seront considérées comme significatives.

9-2 Critères secondaires d'évaluation

Les critères d'évaluation secondaires seront tout d'abord décrits par visite dans chaque bras de l'étude séparément. Pour les paramètres continus, les changements entre l'inclusion et la semaine 24 seront comparés (Rituximab versus placebo) dans une ANCOVA avec ajustement pour la valeur du paramètre à l'inclusion. Si l'hypothèse de normalité des données est clairement intenable, un test non-paramétrique de Mann-Whitney sera réalisé. Cette analyse portera sur :

- Les échelles cliniques
- Le nombre d'articulations gonflées et douloureuses (maximum 64), de points douloureux (18) et le score global évalué par le médecin (échelle visuelle analogique 0 à 100 mm) lors de chaque visite.
- La modification échographique des glandes salivaires (volume et doppler puissance).
- Les paramètres biologiques : vitesse de sédimentation, C réactive protéine, numération formule sanguine, fonction rénale et hépatique, créatine phosphokinase, taux sérique d'IgA, IgG, IgM, anticorps anti-nucléaires et leurs spécificités, facteurs rhumatoïdes, populations lymphocytaires, beta 2 micro globuline et tests centralisés évaluant les conséquences du rituximab sur la fonction lymphocytaire B.
- Les critères objectifs de bouche sèche (salive totale non stimulée) et de sécheresse oculaire (break-up time, test de Schirmer, test au vert de lissamine).
- L'évaluation de l'amélioration des atteintes viscérales par le médecin (échelle visuelle analogique et transmission des données au centre coordinateur).
- Le score histopathologique et l'expression des gènes dans les biopsies des glandes salivaires.

Une analyse exploratoire plus formelle de ces données longitudinales pourra être mise en place par modèle de régression linéaire mixte.

Enfin, la tolérance du produit sera analysée en terme de proportion d'apparition de chacun des effets secondaires retrouvés par les investigateurs.

9-3 Base de données

Les données seront collectées sur des cahiers de protocoles avec duplicata. La saisie sera centralisée, et faite sur masque Epi-Info par le CIC de Brest. La base de donnée sera gérée par le CIC conformément à la procédure reportée en annexe G.

10. CIRCUIT DES PRELEVEMENTS BIOLOGIQUES

Les examens standards seront réalisés dans l'hôpital qui prend en charge les patients. Les tubes de sérums et les glandes salivaires seront congelés sur place puis acheminés à

Brest et collectés au CIC conformément à l'annexe H qui décrit le circuit des prélèvements biologiques et la procédure de stokage. Une partie de ces prélèvements sera dirigée ensuite dans les services d'anatomopathologie et d'immunologie du CHU de Brest.
Pour les patients suivis à Brest, une partie des prélèvements qui doivent être adressés dans les services d'anatomopathologie et d'immunologie seront directement adressés dans ces services sans passer par le CIC pour permettre l'analyse sur les prélèvements frais. La congélation sera ensuite faite et la conservation se fera, comme pour les autres, au CIC.

11. ASPECTS ETHIQUES

Rôle du promoteur

Il est défini par la Loi 2004-806 du 9 août 2004. Le Centre Hospitalier Universitaire de Brest est promoteur de cette recherche.

Comité de Protection des Personnes et Autorités de Tutelles :

Il est de la responsabilité du Promoteur de s'assurer que le protocole de l'essai est soumis à un Comité de Protection des Personnes (CPP), et conformément à la loi n°2004-806 du 09 août 2004 relative à la politique de Santé Publique et à ses décrets d'application, la recherche ne pourra être mise en œuvre qu'après l'avis favorable du CPP et l'autorisation de l'Autorité Compétente.
De plus, aucune modification ne pourra être apportée au protocole sans avis préalable de l'investigateur coordonnateur et accord du Promoteur. Toute modification substantielle au protocole sera alors soumise à l'Autorité Compétente et/ou au CPP (modification d'un critère d'inclusion, prolongation de la durée de l'étude, participation de nouveaux investigateurs…).

Assurance :

Le Promoteur souscrira à une Assurance couvrant sa responsabilité civile pour les dommages potentiels pouvant survenir chez les sujets participant à l'étude.

Déclaration CNIL :

L'anonymat des personnes participant à l'étude sera respecté ; elles auront un droit d'accès et de rectification à tout moment, conformément à la loi Informatique et Liberté. Le fichier informatique utilisé pour la recherche fera l'objet d'une autorisation auprès de la Commission Nationale de l'Informatique et des Libertés (CNIL) de la loi “ informatique et libertés ”.

Réglementation :

Les investigateurs réaliseront l'étude en conformité avec la Déclaration d'Helsinki actualisée, la loi n°88-1138 du 20 décembre 1988 modifiée (Loi Huriet-Sérusclat), la loi n°2004-806 du 9 août 2004 relative à la politique de Santé Publique et ses décrets d'application, la loi « Informatique et Libertés », les Bonnes Pratiques Cliniques et les recommandations ICH.
Un consentement éclairé et une lettre d'information sont joints en annexe B et C.

12. BUDGET

Le budget total est de 240 600 euros, auquel il faut ajouter le prix des unités de traitement, soit **52 800 euros**. Le financement complet a été obtenu grace à un PHRC d'une part et à la fourniture des unités de traitement par le laboratoire Roche d'autre part.

13. BIBIOGRAPHIE

1- Mariette X, Ravaud P, Steinfeld S, Baron G, Goetz J, Hachulla E, et al.. Inefficacy of infliximab in primary Sjogren's syndrome: results of the randomized controlled Trial of Remicade in Primary Sjogren's Syndrome (TRIPSS). Arthritis Rheum 2004;50:1270-6
2- Saraux A, Devauchelle V, Jousse S, Youinou P. Rituximab in rheumatic diseases. Joint Bone Spine. 2007 Jan;74(1):4-6.
3- Pijpe J, van Imhoff GW, Spijkervet FK, Roodenburg JL, Wolbink GJ, Mansour K, Vissink A, Kallenberg CG, Bootsma H. Rituximab treatment in patients with primary Sjogren's syndrome: An open-label phase II study. Arthritis Rheum. 2005;52:2740-50.
4- Seror R, Sordet C, Guillevin L, Hachulla E, Masson C, Ittah M, Candon S, Leguern V, Aouba A, Jean S, Gottenberg JE, Mariette X. Tolerance and efficacy of rituximab and changes in serum B cell biomarkers in patients with systemic complications of primary Sjogren's syndrome. Ann Rheum Dis. 2006 Sep 1; [Epub ahead of print]
5- Devauchelle-Pensec V, Pennec Y, Morvan J, Pers JO, Daridon C, Jousse S, Roudaut A, Jamin C, Renaudineau Y, Quintin I, Cochener-Lamard B, Youinou P, Saraux A. Improvement of Sjögren's syndrome after two infusions of rituximab (anti CD20). Arthritis Rheum, 2007 Mar 15;57(2):310-7.
6- Daridon C, Pers JO, Devauchelle V, Martins-Carvalho C, Hutin P, Pennec YL, Saraux A, Youinou P. Identification of transitional type II B cells in the salivary glands of patients with Sjogren's syndrome. Arthritis Rheum. 2006 Jul;54(7):2280-8.
7- d'Arbonneau F, Pers JO, Devauchelle V, Pennec Y, Saraux A, Youinou P. BAFF-induced changes in B cell antigen receptor-containing lipid rafts in Sjogren's syndrome. Arthritis Rheum. 2006 Jan;54(1):115-26.
8- Binard A, Devauchelle-Pensec V, Fautrel B, Jousse S, Youinou P, Saraux A. Epidemiology of Sjogren's syndrome: where are we now? Clin Exp Rheumatol. 2007 Jan-Feb;25(1):1-4. Review.
9- Jonsson R, Gordon TP, Konttinen YT. Recent advances in understanding molecular mechanisms in the pathogenesis and antibody profile of Sjogren's syndrome. Curr Rheumatol Rep. 2003;5:311-6.
10- Gannot G, Lancaster HE, Fox PC. Clinical course of primary Sjogren's syndrome: salivary, oral, and serologic aspects. J Rheumatol. 2000;27:1905-9.
11- Vinuesa CG, Cook MC. The molecular basis of lymphoid architecture and B cell responses: implications for immunodeficiency and immunopathology. Curr Mol Med 2001;1:689-725.
12- Bohnhorst JO, Bjorgan MB, Thoen JE, Jonsson R, Natvig JB, Thompson KM. Abnormal B cell differentiation in primary Sjogren's syndrome results in a depressed percentage of circulating memory B cells and elevated levels of soluble CD27 that correlate with Serum IgG concentration. Clin Immunol 2002;103:79-88.
13- Hansen A, Odendahl M, Reiter K, Jacobi AM, Feist E, Scholze J, et al. Diminished peripheral blood memory B cells and accumulation of memory B cells in the salivary glands of patients with Sjogren's syndrome. Arthritis Rheum 2002; 46:2160-71.
14- Stott DI, Hiepe F, Hummel M, Steinhauser G, Berek C. Antigen-driven clonal proliferation of B cells within the target tissue of an autoimmune disease. The salivary glands of patients with Sjogren's syndrome J Clin Invest 1998;102:938-46.
15- Stashenko P, Nadler LM, Hardy R, Schlossman SF. Characterization of a human B lymphocyte-specific antigen. J Immunol 1980;125:1678-85.
16- Reff ME, Carner K, Chambers KS, Chinn PC, Leonard JE, Raab R, et al. Depletion of B cells in vivo by a chimeric mouse human monoclonal antibody to CD20. Blood 1994; 83:435-45.
17- Coiffier B, Haioun C, Ketterer N, Engert A, Tilly H, Ma D, et al. Rituximab (anti-CD20 monoclonal antibody) for the treatment of patients with relapsing or refractory aggressive lymphoma: a multicenter phase II study. Blood 1998; 92:1927-32.

18- Maloney DG, Grillo-Lopez AJ, White CA, Bodkin D, Schilder RJ, Neidhart JA, et al. IDEC-C2B8 (Rituximab) anti-CD20 monoclonal antibody therapy in patients with relapsed low-grade non-Hodgkin's lymphoma. Blood 1997; 90:2188-95.
19- Piro LD, White CA, Grillo-Lopez AJ, Janakiraman N, Saven A, Beck TM, et al. Extended Rituximab (anti-CD20 monoclonal antibody) therapy for relapsed or refractory low-grade or follicular non-Hodgkin's lymphoma. Ann Oncol 1999; 10:655-61.
20- McLaughlin P, Grillo-Lopez AJ, Link BK, Levy R, Czuczman MS, Williams ME, et al. Rituximab chimeric anti-CD20 monoclonal antibody therapy for relapsed indolent lymphoma: half of patients respond to a four-dose treatment program. J Clin Oncol 1998;16:2825-33.
21- Patel K, Berman J, Ferber A, Caro J. Refractory autoimmune thrombocytopenic purpura treatment with rituximab. Am J Hematol 2001;67:59-60.
22- Stasi R, Pagano A, Stipa E, Amadori S. Rituximab chimeric anti-CD20 monoclonal antibody treatment for adults with chronic idiopathic thrombocytopenic purpura. Blood 2001:98:952-7.
23- Lee EJ, Kueck B. Rituxan in the treatment of cold agglutinin disease. Blood 1998;92:3490-l.
24- Zaja F, Russo D, Fuga G, Perella G, Baccarani M. Rituximab for myasthenia gravis developing after bone marrow transplant. Neurology 2000;55:1062-3.
25- Ahrens N, Kingreen D, Seltsarn A, Salama A. Treatment of refractory autoimmune haemolytic anaemia with anti-CD20 (rituximab). Br J Haematol 2001;114:244-5.
26- Specks U, Fervenza FC, McDonald TJ, Hogan MCE. Response Wegener's granulomatosis to anti-CD20 chimeric monoclonal antibody therapy. Arthritis Rheum 2001; 44:2836-40.
27- Perrotta S, Locatelli F, La Manna A, Cennamo L, De Stefano P, Nobili B. Anti-CD20 monoclonal antibody (Rituximab) for life-threatening autoimmune haemolytic anaemia in a patient with systemic lupus erythematosus. Br J Haematol 2002; 116:465-67.
28- Edwards JC, Cambridge G. Sustained improvement in rheumatoid arthritis following a protocol designed to deplete B lymphocytes. Rheumatology (Oxford) 2001; 40:205-11.
29- Edwards JC, Szczepanski L, Szechinski J, Filipowicz-Sosnowska A, Emery P, Close DR, et al. Efficacy of B-cell-targeted therapy with rituximab in patients with rheumatoid arthritis. N Engl J Med 2004; 350:2572-2581.
30- Leandro MJ, Edwards JC, Cambridge G, Ehrenstein MR, Isenberg DA. An open study of B lymphocyte depletion in systemic lupus erythematosus. Arthritis Rheum 2002; 46:2673-77.
31- Anolik JH, Sanz I, Looney RJ. B cell depletion therapy in systemic lupus erythematosus. Curr Rheumatol Rep 2003; 5:350-56.
32- Anolik JH, Campbell D, Felgar RE, Young F, Sanz I, Rosenblatt J, Looney RJ. The relationship of FcgammaRIIIa genotype to degree of B cell depletion by rituximab in the treatment of systemic lupus erythematosus. Arthritis Rheum 2003; 48:455-59.
33- Champey J, Corruble E, Gottenberg JE, Buhl C, Meyer T, Caudmont C, Berge E, Pellet J, Hardy P, Mariette X. Quality of life and psychological status in patients with primary Sjogren's syndrome and sicca symptoms without autoimmune features. Arthritis Rheum. 2006 Jun 15;55(3):451-7.
34- Navazesh, M. Methods for collecting saliva. Ann N Y Acad Sci; 1993; 694:72-77.
35- Van Bijsterveld OP, Mackor AJ. Sjogren's syndrome and tear function parameters. Clin Exp Rheumatol, 1989, 7, 151
36- Vitali C, Palombi G, Baldini C, Benucci M, Bombardieri S, Covelli M, et al measurement (m) of disease activity (da) in sjogren's syndrome (sjs) by means of a new scale (sjsdam) developed by the analysis of a cohort of patients collected by the study group for sjs of the italian society of rheumatology. Soumis pour publication.
37- Vitali C, Bombardieri S, Jonsson R, Moutsopoulos HM, Alexander EL, Carsons SE, Daniels TE, Fox PC, Fox RI, Kassan SS, Pillemer SR, Talal N, Weisman MH;

European Study Group on Classification Criteria for Sjogren's Syndrome. Classification criteria for Sjogren's syndrome: a revised version of the European criteria proposed by the American-European Consensus Group. Ann Rheum Dis 2002 Jun;61(6):554-8

38- Emery P, Fleischmann R, Filipowicz-Sosnowska A, Schechtman J, Szczepanski L, Kavanaugh A, Racewicz AJ, van Vollenhoven RF, Li NF, Agarwal S, Hessey EW, Shaw TM; DANCER Study Group. The efficacy and safety of rituximab in patients with active rheumatoid arthritis despite methotrexate treatment: results of a phase IIB randomized, double-blind, placebo-controlled, dose-ranging trial. Arthritis Rheum. 2006 May;54(5):1390-400.

ANNEXE A

METHODES D'EVALUATION DES TRAITEMENTS

S'il existe maintenant un consensus international sur les critères diagnostiques, il n'existe dans la littérature aucun score d'activité permettant de juger de l'effet d'un traitement. Du fait des nombreux symptômes rencontrés, liés à l'atteinte générale pluri organique, il est difficile de privilégier l'un de ces symptômes. Les marqueurs objectifs sont tous sujets à caution, ce qui rend difficile le choix du critère principal.

L'évaluation de l'effet du traitement, pour être pragmatique, doit être aussi proche que possible de l'objectif pour lequel il a été prévu. Dans le cas du syndrome de Sjogrën, c'est l'amélioration de l'état clinique des patients qui est souhaités, et les plaintes principales sont la sécheresse, la fatigue, et la douleur. La plupart des patients se plaignent des trois composantes, mais généralement seulement une ou deux de ces plaintes dominent. Aussi, nous considérons que les patients peuvent être au mieux évalués par trois échelles analogiques portant sur ces paramètres auxquelles on ajoute une échelle globale incluant les atteintes extra glandulaires de la maladie.

.

BIOPSIE DES GLANDES SALIVAIRES ACCESSOIRES (BGSA)

Deux biopsies seront proposées (visite d'inclusion et V5-semaine 24). Elles ont pour but de vérifier le diagnostic et d'évaluer l'évolution de la maladie (importance de l'inflammation et de la fibrose des glandes salivaires). La première biopsie sera proposée à tous les patients sauf si l'on dispose d'un focus score et d'un stade de Chisholm antérieurs. Une partie des glandes salivaires prélevées sera envoyé au CIC de Brest (annexe H). La seconde biopsie sera proposée à 24 semaines (focus score et stade de Chisholm à faire en local) pour évaluer l'efficacité du traitement (notamment par puce à ADN) et une partie des prélèvements effectués seront envoyés au CIC de Brest (annexe H).

Après éversion de la lèvre inférieure, une anesthésie locale est réalisée à l'aide de lidocaïne avec ou sans adrénaline. L'injection se fait à l'aide d'une fine aiguille (type insuline). Un volume de 0,5 ml est en général suffisant. La lèvre est maintenue éversée par un aide opératoire. Une petite incision de 3 à 4 mm de long est réalisée avec une pointe de bistouri en regard de la canine mandibulaire permettant d'éviter les petits rameaux nerveux habituellement plus médians.

A l'aide de ciseaux pointus et courbes, 4 glandes salivaires accessoires sont prélevées, leur extériorisation étant favorisée par une pression manuelle sur le versant externe de la lèvre regard de la micro incision. Un fil de suture est exceptionnellement nécessaire. S'il n'y a pas de fil de suture, l'hémostase est assurée par compression manuelle l'aide d'une compresse. Il n'y a pas de désinfection particulière à effectuer, un simple bain bouche est suffisant juste avant la biopsie.

FLUX SALIVAIRE

Pour évaluer le flux salivaire global non stimulé le patient ne doit ni boire, ni manger et ni fumer une heure avant l'examen. Le test sera réalisé de préférence le matin entre 08h00 et 9h00. Le patient est installé confortablement dans un fauteuil au moins 5 minutes avant de réaliser le test. Il patient doit cracher toutes les minutes pendant 5 minutes dans un tube de centrifugation de 50 cm.

Les échantillons recueillis sont pesés à l'aide d'une balance analytique. L'élément d'analyse s'exprime en ml/min, le volume est déterminé selon la formule : l mg = l ml.

EXAMEN OPHTALMOLOGIQUE

Les examens ophtalmologiques seront comme habituellement le test à la fluorescéine, le test au vert de Lissamine, le test de Schirmer et l'examen à la lampe à fente.

EXAMEN ECHOGRAPHIQUE

Une échographie sera réalisée par le même examinateur dans les centres participants à la sous étude échographique des glandes salivaires. La taille des glandes parotidiennes (hauteur, largeur, épaisseur) et leur aspect en power doppler seront reportés à l'inclusion et à 24 semaines.

Les échographistes auront tous une formation préalable avec étude de leur reproductibilité à Brest (48h de formation et évaluation).

	Droite	gauche
Taille	Hauteur : …………………..mm<br>Largeur : …………………..mm<br>Epaisseur : ………………..mm	Hauteur : …………………..mm<br>Largeur : …………………..mm<br>Epaisseur : ………………..mm
Power doppler	Positif ☐ Négatif ☐<br><br>Stade 1 ☐ 2 ☐ 3 ☐	Positif ☐ Négatif ☐<br><br>Stade 1 ☐ 2 ☐ 3 ☐

EXAMENS IMMUNOLOGIQUES

Pour évaluer les conséquences du traitement sur le plan immunologiques, seront notamment mesurés :

- le taux sérique d'IgG, IgA, IgM,
- les anticorps anti-nucléaires et leurs spécificités,
- les facteurs rhumatoïdes,
- les populations lymphocytaires**,**
- les marqueurs immunologiques d'activité B,
- Anticorps chimériques.

Les biopsies de glandes salivaires seront évaluées de façon centralisée (histologie, immunohistochimie, puces à ADN) avant et après traitement.

CRITERES EUROPEENS « REVISES » DU SYNDROME DE SJÖGREN (1)

1. Symptômes oculaires
Au moins un des 3 critères ci-dessous :
- sensation quotidienne, persistante et gênante d'yeux secs depuis plus de 3 mois
- sensation fréquente de « sable dans les yeux »
- utilisation de larmes artificielles plus de 3 fois par jour.

2. Symptômes buccaux
Au moins un des 3 critères ci-dessous :
- sensation quotidienne de bouche sèche depuis plus de 3 mois
- à l'âge adulte, glandes salivaires enflées de manière répétée ou persistante
- consommation fréquente de liquides pour avaler les aliments secs

3. Signes cliniques ophtalmologiques
Au moins un des 2 tests ci-dessous positifs :
- Test de Schirmer **<** 5 / 5minutes
- Score de Bijsterveld **>** 4

4. Histopathologie
Sialadénite de score ≥ I sur biopsie(s) de glandes salivaires accessoires
(foyer : **>** 50 cellules mononucléées agglomérées ; score = nombre de foyers sur 4 mm2 de tissu glandulaire).

5. Atteinte des glandes salivaires
Au moins un des 3 tests ci-dessous positifs :
- Scintigraphie salivaire
- Scintigraphie parotidienne
- Flux salivaire sans stimulation < 0,5 ml/5 minutes

6. Autoanticorps
- Anti-Ro (SS-A) ou
- Anti-La (SS-B)

Syndrome de Sjögren :
-4/6 critères avec au moins critère 4 ou 6 présent
-au moins 3 des 4 critères objectifs

Critères d'exclusion:
-Irradiation
-Hépatite C
-SIDA
-Lymphome préexistant
-Sarcoidose
-Réaction greffon versus hôte
-Traitement anticholinergique

1- Vitali C, Bombardieri S, Jonsson R, Moutsopoulos HM, Alexander EL, Carsons SE, et al. European Study Group on Classification Criteria for Sjogren's Syndrome. Classification criteria for Sjogren's syndrome: a revised version of the European criteria proposed by the American-European Consensus Group. Ann Rheum Dis 2002 61:554-58

QUESTIONNAIRE DE QUALITE DE VIE SF-36

COMMENT REPONDRE : Les questions qui suivent portent sur votre santé, telle que vous la ressentez. Ces informations nous permettront de mieux savoir comment vous vous sentez dans votre vie de tous les jours.

Veuillez répondre à toutes les questions en entourant le chiffre correspondant à la réponse choisie, comme il est indiqué. Si vous ne savez pas très bien comment répondre, choisissez la réponse la plus proche de votre situation.

1. Dans l'ensemble, pensez-vous que votre santé est :

entourez le chiffre qui correspond à votre choix

-Excellente **1**

-Très bonne **2**

-Bonne..**3**

-Médiocre **4**

-Mauvaise...**5**

2**. Par rapport à il y a 6 mois**, comment trouvez-vous votre état de santé **en ce moment** ?

entourez le chiffre qui correspond à votre choix

-Bien meilleur qu'il y a 6 mois **1**

-Plutôt meilleur **2**

-A peu près pareil...**3**

-Plutôt moins bon...**4**

-Beaucoup moins bon **5**

3. Voici une liste d'activités que vous pouvez avoir à faire dans votre vie de tous les jours. Pour chacune d'entre elles indiquez si **vous êtes limité(e) en raison de votre état de santé actuel**.

entourez le chiffre qui correspond à votre choix, un par ligne

Liste d'activités	**oui, beaucoup limité(e)**	**oui, un peu limité(e)**	**non, pas du tout limité(e)**
**Efforts physiques importants** tels que courir, soulever un objet lourd, faire du sport	**1**	**2**	**3**
**Efforts physiques modérés** tels que déplacer une table, passer l'aspirateur, jouer aux boules	**1**	**2**	**3**
Soulever et porter les courses	**1**	**2**	**3**
Monter **plusieurs étages** par l'escalier	**1**	**2**	**3**
Monter **un étage** par l'escalier	**1**	**2**	**3**
Se pencher en avant, se mettre à genoux, s'accroupir	**1**	**2**	**3**
Marcher **plus d'un km** à pied	**1**	**2**	**3**
Marcher **plusieurs centaines de mètres**	**1**	**2**	**3**
Marcher **une centaine de mètres**	**1**	**2**	**3**
Prendre un bain, une douche ou s'habiller	**1**	**2**	**3**

4. Au cours de ces **4 dernières semaines**, et en raison de votre **état physique**,

entourez le chiffre qui correspond à votre choix,
un par ligne

	OUI	NON
Avez-vous réduit **le temps passé** à votre travail ou à vos activités habituelles	**1**	**2**
Avez-vous **accompli moins** de choses que vous auriez souhaité	**1**	**2**
Avez-vous dû arrêter de faire **certaines choses**	**1**	**2**
Avez-vous eu des **difficultés** à faire votre travail ou toute autre activité (par exemple, cela vous a demandé un effort supplémentaire)	**1**	**2**

5. Au cours de ces **4 dernières semaines**, et en raison de votre **état émotionnel** (comme vous sentir triste, nerveux(se) ou déprimé(e)),

entourez le chiffre qui correspond
à votre choix, un par ligne

	OUI	NON
Avez-vous réduit **le temps passé** à votre travail ou à vos activités habituelles	**1**	**2**
Avez-vous **accompli moins** de choses que vous auriez souhaité	**1**	**2**
Avez-vous eu des **difficultés** à faire ce que vous aviez à faire **avec autant de soin et d'attention** que d'habitude	**1**	**2**

6. Au cours de ces **4 dernières semaines** dans quelle mesure votre état de santé, physique ou émotionnel, vous a t-il gêné(e) dans votre vie sociale et vos relations avec les autres, votre famille, vos amis, vos connaissances ?

entourez le chiffre qui correspond à votre choix

-Pas du tout **1**

-Un petit peu **2**

-Moyennement.. **3**

-Beaucoup... **4**

-Enormément **5**

7. Au cours de ces **4 dernières semaines**, quelle a été l'intensité de vos **douleurs physiques** ?

entourez le chiffre qui correspond à votre choix

-Nulle **1**

-Très faible... **2**

-Faible **3**

-Moyenne.. **4**

-Grande **5**

-Très grande **6**

8. Au cours de ces **4 dernières semaines**, dans quelle mesure vos douleurs physiques vous ont elles limité(e) dans votre travail ou vos activités domestiques ?

entourez le chiffre qui correspond à votre choix

-Pas du tout...**1**

-Un petit peu...**2**

-Moyennement..**3**

-Beaucoup...**4**

-Enormément **5**

9. Les questions qui suivent portent sur comment vous vous êtes senti(e) **au cours de ces 4 dernières semaines**. Pour chaque question, veuillez indiquer la réponse qui vous semble la plus appropriée. **Au cours de ces 4 dernières semaines**, y a-t-il eu des moments où :

entourez le chiffre qui correspond à votre choix, un par ligne

	**en permanence**	**très souvent**	**souvent**	**quelquefois**	**rarement**	**jamais**
Vous vous êtes senti(e) dynamique ?	**1**	**2**	**3**	**4**	**5**	**6**
Vous vous êtes senti(e) très nerveux (se) ?	**1**	**2**	**3**	**4**	**5**	**6**
Vous vous êtes senti(e) si découragé(e) que rien ne pouvait vous remonter le moral ?	**1**	**2**	**3**	**4**	**5**	**6**
Vous vous êtes senti(e) calme et détendu(e) ?	**1**	**2**	**3**	**4**	**5**	**6**
Vous vous êtes senti(e) débordant(e) d'énergie ?	**1**	**2**	**3**	**4**	**5**	**6**
Vous vous êtes senti(e) triste et abattu(e) ?	**1**	**2**	**3**	**4**	**5**	**6**
Vous vous êtes senti(e) épuisé(e) ?	**1**	**2**	**3**	**4**	**5**	**6**
Vous vous êtes senti(e) heureux (se) ?	**1**	**2**	**3**	**4**	**5**	**6**
Vous vous êtes senti(e) fatigué(e) ?	**1**	**2**	**3**	**4**	**5**	**6**

10. Au cours de ces **4 dernières semaines** y a t-il eu des moments où votre **état de santé**, **physique ou émotionnel**, vous a gêné(e) dans votre vie sociale et vos relations avec les autres, votre famille, vos amis, vos connaissances ?

entourez le chiffre qui correspond à votre choix

-En permanence **1**

-Une bonne partie du temps.................................. **2**

-De temps en temps **3**

-Rarement **4**

-Jamais.. **5**

11. Indiquez, pour **chacune** des phrases suivantes, dans quelle mesure elles sont vraies ou fausses dans votre cas :

entourez le chiffre qui correspond à votre choix, un par ligne

	**totalement vraie**	**plutôt vraie**	**je ne sais pas**	**plutôt fausse**	**totalement fausse**
Je tombe malade plus facilement que les autres	**1**	**2**	**3**	**4**	**5**
Je me porte aussi bien que n'importe qui	**1**	**2**	**3**	**4**	**5**
Je m'attends à ce que ma santé se dégrade	**1**	**2**	**3**	**4**	**5**
Je suis en excellente santé	**1**	**2**	**3**	**4**	**5**

ANNEXE B

INFORMATION DESTINEE AU PATIENT

TOLERANCE ET EFFICACITE DU RITUXIMAB DANS LE SYNDROME DE SJOGREN (TEARS STUDY)

Promoteur : CHU de Brest , 2 avenue Foch, 29609 Brest Cédex
Investigateur Principal : Pr Alain Saraux, Service de Rhumatologie, CHU de Brest, Avenue Tanguy Prigent - 29609 Brest Cedex

Vous êtes atteint d'un syndrome de Sjögren primitif. Les traitements susceptibles d'améliorer l'évolution de la maladie ne se sont pas montrés suffisamment efficaces dans votre cas.

Ce protocole thérapeutique est proposé aux patients qui ont est un syndrome de Sjogren soit **récent** (moins de 10 ans à compter de la première manifestation signalée à un médecin ou constatée par un médecin) **et actif**, soit **avec atteinte viscérale active**. **Sont** néanmoins **exclus :**

- Les patients ayant des signes d’autres maladies auto-immunes, une pathologie sévère progressive ou non contrôlée au niveau rénal, hépatique, hématologique, endocrinien, pulmonaire, cardiaque, neurologique ou cérébral, une allergie à un anticorps de souris, un alcoolisme ou une toxicomanie, un traitement non stable.
- Les patients ayant eu un traitement immunosuppresseur récent (au cours des 4 semaines précédant l’inclusion), une infection sérieuse (telle qu’une hépatite, une pneumonie ou une pyélonéphrite) au cours des 3 mois précédant l’inclusion ou une infection opportunistes antérieure non guérie.
- Les patients à risque infectieux ou ayant une infection documentée.
- Les patients devant subir intervention chirurgicale prévue lors de la durée de l’étude.
- Les patients ayant eu une pathologie tumorale au cours des 5 années passées.
- Les patients non affiliés à la Sécurité Sociale.

Des études expérimentales ont montré que le Rituximab (Mabthera°), qui est une molécule capable de diminuer sélectivement le nombre de lymphocytes B, est efficace dans les maladies auto-immunes telles que le lupus érythémateux et la polyarthrite rhumatoïde. Le syndrome de Sjögren primitif étant la maladie auto-immune au cours de laquelle les lymphocytes B sont le plus impliqués, il parait vraisemblable que ce traitement soit efficace dans cette affection. Trois études ouvertes portant chacune sur une quinzaine de patients suggèrent une bonne efficacité et une bonne tolérance du produit dans le syndrome de Sjögren primitif.

Nous vous proposons de participer à une étude dont l’objectif est de comparer l’efficacité du rituximab à celle d’un placebo, à 24 semaines et de vérifier si l'association de Rituximab à vos traitements habituels permet d'améliorer leur résultat. Le bénéfice escompté pour vous est une amélioration de toutes les manifestations dues au syndrome de Sjogren. **Le Rituximab (Mabthera°),** est un médicament qui a obtenu une autorisation européenne validée en France pour le traitement de maladies hématologique (lymphome) et dans la polyarthrite rhumatoïde. **Les études antérieures démontrent globalement une bonne tolérance dans les maladies auto-immunes, en l’absence d’immuno-suppression ou de lymphome.** Les effets secondaires qui ont été observés au cours des lymphomes sont en partie liée à la lyse cellulaire, ce qui ne devrait pas être observé dans les maladies autoimmunes avec une telle intensité.

Paraphes Patient : *Paraphes Investigateur :*

Les principaux effets secondaires qui pourraient être observées dans le cadre du traitement d'un syndrome de Sjogren sont :

-Des allergies (cutanée ou pulmonaires) : Réactions liées à la perfusion, habituellement au cours des deux premières heures. Ces événements comprenaient d'autres symptômes tels que rougeurs, angiooedème, nausées, rash/urticaire, fatigue, céphalées, irritation laryngée, rhinite, vomissements. Il a également été observé, moins fréquemment, une exacerbation de pathologies cardiaques préexistantes comme l'angor ou l'insuffisance cardiaque congestive. L'incidence des symptômes liés à la perfusion décroît considérablement au cours des perfusions ultérieures.

-Des troubles tensionnels : Les événements le plus fréquemment rapportés ont été une hypotension et une hypertension artérielle.

-**Des signes cardiaques** : Des événements cardiaques sévères, incluant insuffisance cardiaque et infarctus du myocarde ont été observés principalement chez les patients présentant des antécédents cardiaques.

- **Des vascularites**, principalement cutanées telles que la vascularite leucocytoclasique ont été très rarement rapportées.

- Des infections : Mabthera induit une déplétion en lymphocytes B chez 70 à 80% des patients, mais il n'a été associé à une diminution des immunoglobulines sériques que chez un faible nombre de patients.

- Des anomalies hématologiques, habituellement discrètes et réversibles sont survenues chez une minorité de patients.

- Des affections de peau et annexes : Des réactions cutanées bulleuses sévères, comprenant des cas fatals, ont été exceptionnellement rapportés.

- Des affections du système nerveux : De rares cas de neuropathies crâniennes avec ou sans neuropathie périphériques ont été rapportés. Des signes et des symptômes de neuropathie crânienne, tels que atteinte sévère de la vision, perte de l'audition, perte d'autres sens et paralysie nerveuse faciale, ont été observés dans des délais variables, jusqu'à plusieurs mois après la fin du traitement par Mabthera. Ils peuvent, rarement, être sévères, ce qui justifie une surveillance étroite lors des perfusions puis tout au long de l'étude. **Dans le cadre de lupus et de vascularite quelques cas de leucoencéphalite multifocale progressive** d'évolution fatale ont été décrits, et si ce diagnostic est supecté les patients doivent avoir une imagerie par résonnance magnétique (IRM) et une ponction lombaire.

Par ailleurs, une dose de cortisone et une de paracétamol seront injectées avant chaque perfusion, et vous devrez prendre un comprimé anti allergique pour améliorer la tolérance. La prise de cortisone peut avoir des effets secondaires par elle-même (dont énervement, poussée de tension, recrudescence d'acné).

En pratique :

-Le médecin qui vous prend en charge vérifiera avant le traitement que vous n'avez pas d'infection (dont hépatite B et C et infection VIH), que vos vaccins sont à jour, que vous avez une contraception.

-Si vous n'avez pas de contre indication le traitement sera administré sous forme de deux perfusion en hospitalisation d'une journée et vous serez surveillé durant les perfusions car des réactions sont possibles (tremblement, fièvre, baisse de tension, picotement des lèvre). En général, la baisse du débit de perfusion permet de continuer sans problème.

-Deux heures après les perfusions, vous rentrerez chez vous. Rarement, des réactions décalées surveinnent et en ce cas vous pourrez rappeler le service hospitalier (tel :……………………………)

-Dans les jours qui suivent et à distance, en cas de signes d'infection ou d'allergie (fièvre, frissons, maux de gorge, douleurs articulaires, toux, urines qui brulent), il faudra aussi prévenir le service hospitalier.

Compte tenu du faible risque du traitement, des risques connus de la maladie en terme de conséquences irréversibles de l'évolution sur les glandes salivaires au cours des premières années de la maladie, et des risques liés aux atteintes viscérales, la balance bénéfice risque devrait être positive, en particulier dans les formes avec atteinte viscérale. .

Si vous acceptez de participer à cet essai vous vous engagez à consulter le médecin chargé de vous suivre dans cette étude en cas d'apparition de signes évoquant une infection ou une allergie. En cas de grossesse survenant durant l'étude, il sera important d'en avertir le médecin responsable rapidement. En outre, une contraception efficace est nécessaire dans l'année qui suit la dernière perfusion.

Cent vingt patients seront inclus dans l'étude. Soixante recevront le traitement et soixante un placebo. Une semaine après la visite d'inclusion, il vous sera prescrit deux perfusions, au cours d'une hospitalisation d'une journée, à une semaine d'intervalle. Vous bénéficierez d'une surveillance médicale pendant les 2 à 4 heures de la perfusion et les 2 heures suivantes. Un matériel et des médicaments spécifiques destinés à traiter les éventuelles réactions seront disponibles sur le lieu de la perfusion qui sera réalisée sous la supervision d'un médecin spécialiste. Puis, vous aurez une visite à l'hôpital aux semaines 6, 16, 24, 36, et 48 (à compter de la première perfusion).

Avec le Rituximab, il n'y a pas d'interaction médicamenteuse particulière, mais vous ne devrez prendre que les médicaments que vous autorisera votre médecin pendant l'étude : la cortisone, le chlorydrate de pilocarpine (préparation magistrale, collyre buvable, Salagen®), la cévimeline (Exovac®), le méthotrexate, les inhibiteurs de la recapture de la sérotonine, les benzodiazépines et apparentés utilisés seront autorisés mais vous ne devrez pas modifier la posologie au cours de l'étude sans l'accord de votre médecin. Thalidomide, anti TNF alpha, ciclosporine en collyre, endoxan et chloraminophène seront interdit. Tous les autres médicaments (notamment les antiinflammatoires, les traitements de la douleur, les traitements locaux ophtalmologiques) seront autorisés.

Cette étude s'étalera sur 2 ans à partir de novembre 2007. Pour vous, elle durera donc **48 semaines** au cours desquelles votre médecin évaluera le résultat du traitement. U**n suivi post étude vous sera proposé** pour rechercher de rares effets secondaires tardifs et juger de l'efficacité à long terme, avec une consultation tous les 6 mois pendant 18 mois. **A partir de la semaine 36, en cas de réponse au traitement (amélioration de 30%) puis de rechute, deux nouvelles perfusions vous seront proposées lors des visites de suivi**: Ce retraitement pourra être fait une fois chez tous les patients qui auront été améliorés et qui le souhaiteront jusqu'à la levée de l'aveugle.

Dans tous les centres, une biopsie des glandes salivaires accessoires sera proposée avant de commencer le traitement pour vérifier le diagnostic et évaluer l'évolution de la maladie (importance de l'inflammation et de la fibrose des glandes salivaires). Une seconde biopsie sera proposée à 24 semaines pour évaluer l'efficacité du traitement (diminution de l'inflammation, des cellules inflammatoires, modification des marqueurs d'activités par puce à ADN notamment). Cette biopsie consistera en un simple prélèvement de quelques millimètres réalisé au niveau de la lèvre inférieure (face interne) après une anesthésie locale, comparable à celle que l'on réalise pour des soins dentaires. Il n'existe aucun risque grave mais vous pourrez ressentir un petit inconfort pendant quelques heures avec rarement une sensation d'anesthésie qui généralement régresse rapidement. L'examen permettra une analyse des cellules contenues dans la glandes (une partie sera gardée en vue d'une étude des cellules présentes dans la glande et d'une étude des expressions des gènes dans la glande salivaire avant et après traitement).

Paraphes Patient : *Paraphes Investigateur :*

Une consultation ophtalmologique (ou au moins un test simple de Shirmer) et une évaluation de la sécheresse buccale seront faits à la visite initiale puis à 6, 16, 24, 36 et 48 semaines.

Une **échographie** des glandes salivaires sera réalisée à la visite initiale et à 24 semaines dans les centres de **Brest, Lille, Rennes et Rouen**.

Par ailleurs, à chaque visite, il faudra vous soumettre à un examen clinique (comportant un interrogatoire, une évaluation de la sécheresse buccale, un examen des articulations et des tendons, un

examen général) **et à des prises de sang** (au maximum 5 à 10 tubes de sang de 5 à 10 ml à chaque fois) qui seront faites avant l'essai, lors des perfusions, puis lors des visites de suivi. Ces prises de sang ont pour objectif d'éliminer des contre-indications au produit, et de suivre les conséquences du traitement sur le comportement des cellules du sang et les facteurs qui modifient l'efficacité du traitement. Il vous sera aussi demandé de **remplir un questionnaire à chaque visite** pour que nous puissions évaluer l'impact du traitement sur la qualité de vie.

Durant cette étude, vous serez suivi(e) par le Dr...de l'hopital: ....Tel : ……………………que vous pourrez contacter pour toute information complémentaire.

Votre **participation à** cet essai est **volontaire** et vous n'êtes en aucun cas obligé à y prendre part. Si vous refusez d'y participer pour quelque motif que ce soit, la qualité de vos soins n'en sera nullement affectée. Si au cours de cet essai, de nouvelles informations étaient disponibles, pouvant remettre en cause votre participation, le médecin chargé de cette recherche vous les communiquera et s'assurera que vous souhaitez continuer à y participer.
Vous pourrez arrêter votre participation à ce protocole à tout moment. Si c'est le cas, le médecin chargé de cet essai vous proposera, si vous le souhaitez, un traitement qu'il jugera adéquat à votre cas et vous demandera de revenir en consultation pour un bilan faisant suite à votre participation à l'essai. Il peut décider votre sortie de l'essai s'il le juge nécessaire, notamment pour votre bien être.

A votre demande, les résultats globaux de l'étude à laquelle vous avez accepté de participer vous seront communiqués.
Ce protocole a reçu un avis favorable du Comité pour la Protection des Personnes Ouest VI le 24 Aout 2007 et a été autorisé par l'AFSSAPS. Le promoteur de l'étude, le CHU de Brest, a souscrit une assurance spécifique (contrat SHAM N°111650), conformément à la loi du 9 août 2004
Dans le cadre de la recherche biomédicale à laquelle le CHU de Brest vous propose de participer, un traitement de vos données personnelles va être mis en œuvre pour permettre d'analyser les résultats de la recherche. A cette fin, les données médicales vous concernant seront transmises au Promoteur de la recherche. Ces données seront identifiées par un numéro de code et vos initiales. Ces données pourront également, dans des conditions assurant leur confidentialité, être transmises aux Autorités de Santé Françaises. Conformément aux dispositions de la loi relative à l'informatique, aux fichiers et aux libertés, cette étude est déclarée à la CNIL et vous disposez d'un droit d'accès et de rectification. Vous disposez également d'un droit d'opposition à la transmission des données couvertes par le secret professionnel susceptibles d'être utilisées dans le cadre de cette recherche et d'être traitées.
Vous pouvez également accéder directement ou par l'intermédiaire d'un médecin de votre choix à l'ensemble de vos données médicales en application des dispositions de l'article L1111-7 du Code de la Santé Publique. Ces droits s'exercent auprès du médecin qui vous suit dans le cadre de la recherche et qui connaît votre identité.
Votre anonymat sera respecté et les résultats de l'étude ne seront utilisés qu'à des fins scientifiques.
Votre médecin ou son représentant seront disponibles pour répondre à toutes les questions qui pourraient survenir en rapport avec le traitement décrit ou le déroulement de l'étude.

Paraphes Patient : *Paraphes Investigateur :*

ANNEXE C

CONSENTEMENT DU PATIENT

Je soussigné(e).
Nom :
Prénom :
Accepte de participer à l'étude clinique intitulée : **TOLERANCE ET EFFICACITE DU RITUXIMAB DANS LE SYNDROME DE SJOGREN (Tears study)**

Le Dr .... m'a expliqué en détail la nature, les bénéfices, les risques et les contraintes de l'essai et m'en a précisé la durée et le déroulement. Il m'a laissé toute latitude pour poser des questions et j'ai eu des réponses satisfaisantes et un délai de réflexion suffisant.
J'ai bien compris la nécessité d'une contraception (sauf ménopause) pendant l'année suivant la dernière perfusion.
Ma participation est totalement volontaire. J'ai bien compris toutes les informations concernant cette recherche et donne mon accord pour y participer. En cas d'événement indésirable ou de problème, ou si j'ai d'autres questions au cours de ma participation, je pourrai contacter le médecin chargé de cette recherche au [n° de téléphone] ....
S'il y a lieu et avec mon accord, mon médecin traitant sera averti de ma participation à cette recherche.
J'ai la liberté d'arrêter à tout moment ma participation, quelle qu'en soit la raison, sans compromettre la qualité des soins qui continueront à m'être prodigués. J'en informerai alors le médecin chargé de cette recherche qui me proposera, si je le souhaite, un autre traitement.
Je suis affilié(e) à la sécurité sociale, cependant tous les frais liés à cette recherche seront intégralement pris en charge par le promoteur. Ma participation est libre de toute charge de ma part.
Les données personnelles restent confidentielles et ne sont consultées que par les personnes qui collaborent directement à la recherche ou par un représentant des autorités de santé.

J'accepte que les données enregistrées à l'occasion de cette recherche puissent faire l'objet d'un traitement informatisé par le promoteur ou pour son compte. J'ai bien noté que le droit d'accès prévu par la loi du 6 janvier 1978 modifiée relative à l'informatique, aux fichiers et aux libertés s'exerce à tout moment auprès du médecin qui me suit dans le cadre de la recherche et qui connaît mon identité. Je pourrai exercer mon droit de rectification et d'opposition auprès de ce même médecin qui contactera le promoteur de la recherche.

J'ACCEPTE DE PARTICIPER A CETTE RECHERCHE DANS LES CONDITIONS PRECISEES CI-DESSUS.
Mon consentement ne décharge pas les organisateurs de la recherche de leurs responsabilités. Je conserve tous mes droits garantis par la loi.
J'accepte qu'un exemplaire de ce document soit détenu à titre confidentiel par le médecin responsable et reconnais qu'un exemplaire m'a été remis.

Partie à remplir par le Médecin		***Partie à remplir par le patient***	
Nom et prénom du Médecin		Nom et prénom du patient	
Signature du Médecin	Date	Signature du patient	Date

Un exemplaire cosigné pour le patient, un exemplaire cosigné pour l'investigateur et une copie pour le promoteur

ANNEXE D

EEFFET SECONDAIRE GRAVE

FICHE DE DECLARATION

EVENEMENT INDESIRABLE GRAVE / GROSSESSE

LORS DE L'ESSAI CLINIQUE

NOTE A L'INVESTIGATEUR

- Dès que vous avez connaissance d'un événement indésirable grave / grossesse, merci de nous adresser cette fiche ***dans les 24 heures.***
Lors de cette ***déclaration initiale****,* ***toutes*** *les rubriques (en fonction des informations dont vous disposez ce jour) doivent être complétées.*

- Merci de nous adresser ***dès que possible*** *les informations complémentaires dont vous disposez.*
Lors de ces ***déclarations complémentaires****, noter uniquement les informations complémentaires / corrections / précisions.*

- Préciser ***à chaque fois*** *votre opinion sur la* ***causalité*** *de l'événement, y compris lors de la (des) déclaration(s) complémentaire(s), au regard des nouvelles informations qui sont à votre disposition.*

- Merci de ***signer*** *chaque déclaration et d'agrafer l'accusé de réception de ce fax à cette fiche.*

**EMETTEUR :**	**DESTINATAIRE :**
Nom de l'investigateur :	Délégation à la Recherche Clinique et à l'Innovation<br>Fax : 02-98-22-31-83

Date de l'envoi : …..../……/………

Nombre de pages *(cette page de couverture incluse) :* |_4_| + |____|

FICHE DE DECLARATION

EVENEMENT INDESIRABLE GRAVE / GROSSESSE

FICHE DE DECLARATION
D'EVENEMENT INDESIRABLE GRAVE OU DE GROSSESSE

N° Protocole : ……………………… N° Centre : |__|__|

☐ Déclaration Initiale ☐ Déclaration Complémentaire N° |__|__|

1. PATIENT

Nom : |__|__| Prénom : |__|__| Numéro patient : |__|__| - |__|__|

Sexe : F ☐ M ☐ Date de naissance : |__|__| - |__|__|- |__|__|

Date d'inclusion : |__|__| - |__|__| - |__|__| Date de Mise sous Traitement : |__|__| - |__|__| - |__|__|

Antécédents significatifs *(préciser les dates)* :
……
……
………

2. EVENEMENT INDESIRABLE GRAVE / GROSSESSE

Date de survenue de l'évènement : |__|__| - |__|__| - |__|__|

L'évènement est sérieux car il s'agit :

☐ Décès (date : |__|__| - |__|__| - |__|__|)
☐ Mise en jeu du pronostic vital
☐ Invalidité ou une incapacité
☐ Hospitalisation ou prolongation d'hospitalisation Du …../……./…… au …../……./…… ou en cours ☐

☐ Potentiellement grave (comme certaines anomalies biologiques)
☐ Anomalie, malformation congénitale
☐ Evènement médicalement important
☐ Grossesse

Description de l'évènement :

……
……
………
……
…………………………………………………………………

3. PRODUIT A L'ETUDE : RITUXIMAB (EN I.V.)

Dates	Dose reçue	Durée perfusion

4. MEDICAMENTS CONCOMITANTS

☐ Oui ☐ Non ☐ Pas d'information

(Ne pas mentionner les médicaments prescrits pour traiter l'EIG)

Nom du produit	Voie	Posologie	Date de Début	Date de fin	Indication
			…../……./……	…../……./…… ☐ en cours	
			…../……./……	…../……./…… ☐ en cours	
			…../……./……	…../……./…… ☐ en cours	

**Traitement(s) de l'évènement indésirable (Si oui,** précisez) : ☐ Oui ☐ Non ☐ Pas d'information					
**Nom du produit**	**Voie**	**Posologie**	**Date de Début**	**Date de fin**	**Indication**
			…../……./……	…../……./……<br>☐ en cours	
			…../……./……	…../……./……<br>☐ en cours	
			…../……./……	…../……./……<br>☐ en cours	

Autre(s) traitement(s) non médicamenteux :
………
………
………

5. EVOLUTION DE L'EVENEMENT

Evolution de l'évènement indésirable grave :
☐ Guérison sans séquelles
☐ Guérison avec séquelles
☐ Amélioration
☐ Inchangé
☐ Aggravation
☐ Inconnue
☐ Décès (cause, date :
…………………………………………….)

Disparition de l'évènement indésirable après arrêt du traitement ? ☐ Oui ☐ Non ☐ NA

Réapparition de l'évènement après réintroduction ? ☐ Oui ☐ Non ☐ NA

Date de résolution de l'évènement : …../……./……

6. CONCLUSIONS DE L'INVESTIGATEUR

Quelle est, selon vous, la relation entre l'évènement indésirable et la perfusion de RITUXIMAB :
☐ Probable ☐ Possible ☐ Non lié ☐ Ne sait pas

Envisagez-vous une ou plusieurs autres causes ? ☐ **Oui** ☐ **Non**
Si oui, précisez :
☐ **Les conditions de la recherche** *(toute contrainte consécutive au protocole de cet essai ou imposé par lui ;ceci inclut notamment, outre le médicament évalué, le produit associé, l'absence de traitement, les investigations pratiquées et leurs conditions réalisées)*
☐ **Un ou plusieurs autres médicaments**. Précisez :
…………………………………………………………………………………
☐ **La progression de la maladie**
☐ **Une autre cause**. Précisez :
………

L'évènement est-il :
☐ attendu ☐ inattendu

COMMENTAIRES :
………
………
…………………………………………………………………………………………

………
………………………………………………

Date : ……../………./………

Nom Investigateur : ……………………………… **Signature :**

……………………………………………………………………………………………

ANNEXE E

CARACTERISTIQUES DU RITUXIMAB (MABTHERA°)

* MABTHERA®

rituximab

FORMES et PRÉSENTATIONS (début page)

Solution à diluer (limpide, incolore) pour perfusion IV à 100 mg/10 ml : Flacons de 10 ml à usage unique, boîte de 2.*Solution à diluer (limpide, incolore) pour perfusion IV à 500 mg/50 ml :* Flacon de 50 ml à usage unique, boîte unitaire.

COMPOSITION (début page)

Solution à 100 mg/10 ml :	*p flacon*
Rituximab* (DCI)	100 mg
Solution à 500 mg/50 ml :	*p flacon*
Rituximab* (DCI)	500 mg

Excipients (communs) : citrate de sodium, polysorbate 80, chlorure de sodium, hydroxyde de sodium, acide chlorhydrique, eau ppi.
Chaque ml de solution contient 10 mg de rituximab.
* anticorps monoclonal chimérique murin/humain obtenu par génie génétique ; il s'agit d'une immunoglobuline glycosylée associant d'une part les régions constantes d'une IgG1 humaine et d'autre part les régions variables des chaînes légères et lourdes d'origine murine. Cet anticorps est produit par une culture de cellules de mammifères (ovaires de hamster chinois) et purifié par chromatographie d'affinité et échange d'ions, comportant des procédés d'inactivation et d'élimination virales spécifiques.

DC INDICATIONS (début page)

Lymphomes non hodgkiniens :

MabThera est indiqué pour le traitement de patients atteints de lymphomes folliculaires de stade III-IV en cas de chimiorésistance ou à partir de la deuxième rechute après chimiothérapie.
MabThera est indiqué en association à une chimiothérapie « CVP » pour le traitement des patients présentant un lymphome folliculaire de stade III-IV n'ayant jamais été précédemment traités.
MabThera en traitement d'entretien est indiqué chez les patients présentant un lymphome folliculaire en rechute ou réfractaire répondant à un traitement d'induction par chimiothérapie avec ou sans MabThera.
MabThera est indiqué en association à une chimiothérapie « CHOP » pour le traitement des patients présentant un lymphome non hodgkinien agressif diffus à grandes cellules B, CD20 positif.
(Cf Pharmacodynamie pour plus d'informations.)

Polyarthrite rhumatoïde :

MabThera en association au méthotrexate est indiqué pour le traitement de la polyarthrite rhumatoïde active, sévère, chez les patients adultes qui ont présenté une réponse inadéquate ou une intolérance aux traitements de fond, dont au moins un anti-TNF (inhibiteur du facteur de nécrose tumorale).

DC POSOLOGIE ET MODE D'ADMINISTRATION (début page)

Posologie :

Lymphomes non hodgkiniens :

Une prémédication par glucocorticoïde doit être envisagée si MabThera n'est pas associé à une chimiothérapie contenant un glucocorticoïde (« CHOP » ou « CVP ») pour le traitement d'un lymphome non hodgkinien.

Lymphome folliculaire non hodgkinien :

En monothérapie chez l'adulte, la posologie recommandée de MabThera est de 375 mg/m^2 de surface corporelle, administrés en perfusion intraveineuse une fois par semaine pendant 4 semaines.

En association à une chimiothérapie « CVP », la posologie recommandée de MabThera est de 375 mg/m^2 de surface corporelle, pendant 8 cures (1 cure tous les 21 jours), administrés le premier jour de chaque cure de chimiothérapie et ceci après administration IV du glucocorticoïde du protocole « CVP ».

Reprise du traitement après rechute dans le lymphome non hodgkinien :

Des patients qui avaient répondu à un premier traitement par MabThera ont été traités à nouveau par MabThera à la posologie de 375 mg/m^2 de surface corporelle, administrés en perfusion intraveineuse une fois par semaine pendant quatre semaines (cf Pharmacodynamie).

Traitement d'entretien :

Les patients qui ont répondu à un traitement d'induction peuvent recevoir un traitement d'entretien par MabThera à la posologie de 375 mg/m^2 de surface corporelle, administrés une fois tous les 3 mois jusqu'à progression de la maladie ou pendant une durée maximale de 2 ans.

Lymphome non hodgkinien agressif diffus à grandes cellules B :

MabThera doit être utilisé en association avec une chimiothérapie « CHOP ». La posologie recommandée est de 375 mg/m^2 de surface corporelle, administrés le premier jour de chaque cure de chimiothérapie, pendant 8 cures, après administration IV du glucocorticoïde du protocole « CHOP ». La tolérance et l'efficacité de MabThera n'ont pas été démontrées en association à d'autres chimiothérapies.

Première perfusion :

Il est recommandé de débuter la perfusion à une vitesse de 50 mg/h ; après les 30 premières minutes, la vitesse de la perfusion pourra être augmentée par paliers de 50 mg/h toutes les 30 minutes, jusqu'à un maximum de 400 mg/h.

Perfusions ultérieures :

Lors des perfusions ultérieures de MabThera, la vitesse initiale pourra être de 100 mg/h, puis augmentée de 100 mg/h toutes les 30 minutes jusqu'à un maximum de 400 mg/h.

Ajustement posologique pendant le traitement :

Aucune réduction de la dose de MabThera n'est recommandée. Lorsque MabThera est associé à une chimiothérapie « CHOP » ou « CVP », la réduction de dose pour les médicaments de chimiothérapie doit être appliquée selon le schéma habituel.

Polyarthrite rhumatoïde :

Un traitement par MabThera est constitué de 2 perfusions IV de 1000 mg. La posologie recommandée de MabThera est de 1000 mg par perfusion IV, suivie d'une deuxième perfusion IV. de 1000 mg à deux semaines d'intervalle.

L'activité de la maladie doit être régulièrement surveillée. Les données cliniques d'efficacité et de tolérance relatives au retraitement par MabThera sont limitées. Dans une petite cohorte, environ 600 patients présentant des signes d'activité de leur maladie ont été retraités 6 à 12 mois après le traitement précédent. Ces patients ont reçu 2 à 5 traitements par MabThera (cf Effets indésirables et Pharmacodynamie). Certains patients ont développé des anticorps humains antichimériques (HACA) après les deux premières perfusions de MabThera (cf Pharmacodynamie). La présence d'HACA peut être associée à une aggravation des réactions liées à la perfusion ou à des réactions allergiques suivant la seconde perfusion des traitements ultérieurs. Chez un patient présentant des HACA, un échec de déplétion lymphocytaire B a été observé après plusieurs traitements. Par conséquent, le rapport bénéfice/risque du traitement par MabThera doit être soigneusement évalué avant le retraitement. L'intervalle entre deux traitements ne doit pas être inférieur à 16 semaines.

Il est possible de poursuivre un traitement par glucocorticoïdes, salicylés, anti-inflammatoires non stéroïdiens ou analgésiques, pendant un traitement par MabThera.

Les patients atteints de polyarthrite rhumatoïde doivent recevoir 100 mg de méthylprednisolone par voie intraveineuse 30 minutes avant MabThera pour diminuer la fréquence et la sévérité des réactions aiguës liées à la perfusion (cf Mode d'administration).

Première perfusion de chaque traitement :

Ce document est la propriété des Centres d'Investigation Clinique. Toute reproduction, même partielle, ne peut être effectuée sans autorisation.

Il est recommandé de débuter la perfusion à une vitesse de 50 mg/h ; après les 30 premières minutes, la vitesse de la perfusion pourra être augmentée par paliers de 50 mg/h toutes les 30 minutes, jusqu'à un maximum de 400 mg/h.

Deuxième perfusion de chaque traitement :

La vitesse initiale pourra être de 100 mg/h, puis augmentée par paliers de 100 mg/h toutes les 30 minutes, jusqu'à un maximum de 400 mg/h.

Populations particulières :

Enfant :

En raison de l'insuffisance des données de tolérance et d'efficacité, l'utilisation de MabThera n'est pas recommandée chez l'enfant.

Patient âgé :

Aucune adaptation posologique n'est nécessaire chez les patients âgés (plus de 65 ans).

Mode d'administration :

La solution de MabThera préparée doit être administrée en perfusion intraveineuse par une voie spécifique. La solution diluée pour perfusion IV ne doit pas être injectée rapidement ni en bolus. Les perfusions de MabThera doivent être administrées dans un environnement où l'ensemble des moyens de réanimation sont immédiatement disponibles et sous étroit contrôle d'un médecin expérimenté.

Une prémédication avec un antipyrétique et un antihistaminique, par exemple paracétamol et diphénhydramine, doit être systématiquement instaurée avant chaque perfusion de MabThera. La prémédication par glucocorticoïde doit également être envisagée (cf Posologie). Les patients doivent être étroitement surveillés en cas de début d'un syndrome de relargage de cytokines (cf Mises en garde/Précautions d'emploi). Chez les patients qui développent des signes évidents de réaction grave, particulièrement dyspnée sévère, bronchospasme ou hypoxie, la perfusion doit être interrompue immédiatement. Chez les patients atteints d'un lymphome non hodgkinien, il faudra alors pratiquer des examens biologiques appropriés pour mettre en évidence un syndrome de lyse tumorale et une radiographie thoracique pour détecter un infiltrat pulmonaire. Chez tous les patients, la perfusion ne doit pas être reprise avant disparition complète de tous les symptômes et avant normalisation des résultats biologiques et des clichés pulmonaires. La perfusion peut alors être reprise à une vitesse réduite au moins de moitié par rapport à la vitesse initiale. Si des réactions indésirables graves surviennent de nouveau, l'arrêt du traitement doit être sérieusement envisagé au cas par cas.

Des réactions légères ou modérées liées à la perfusion (cf Effets indésirables) répondent habituellement à une réduction de la vitesse de la perfusion. Celle-ci peut être augmentée en fonction de l'amélioration des symptômes.

DC CONTRE-INDICATIONS (début page)

Contre-indications d'utilisation dans les lymphomes non hodgkiniens :

- Hypersensibilité connue à la substance active ou à l'un des excipients, ou aux protéines murines.

Contre-indications d'utilisation dans la polyarthrite rhumatoïde :

- Hypersensibilité connue à la substance active ou à l'un des excipients, ou aux protéines murines.
- Infections sévères évolutives (cf Mises en garde/Précautions d'emploi).
- Insuffisance cardiaque sévère (NYHA classe IV) ou maladie cardiaque sévère non contrôlée.

DC MISES EN GARDE et PRÉCAUTIONS D'EMPLOI (début page)

Lymphomes non hodgkiniens :

- Les patients ayant une masse tumorale importante ou un nombre élevé (>= 25 × 10^9/l) de cellules malignes circulantes pourraient être plus exposés à l'apparition d'un syndrome sévère de relargage de cytokines et ne devront donc être traités qu'en cas d'échec des autres alternatives thérapeutiques, et avec une très grande prudence. Ces patients doivent être très étroitement surveillés tout au long de la première perfusion, en envisageant de réduire la vitesse de cette perfusion.

Ce document est la propriété des Centres d'Investigation Clinique. Toute reproduction, même partielle, ne peut être effectuée sans autorisation.

- Le syndrome grave de relargage de cytokines est caractérisé par une dyspnée sévère, souvent accompagnée de bronchospasme et d'hypoxie, associée à de la fièvre, des frissons, des tremblements, de l'urticaire et des angio-oedèmes. Ce syndrome peut s'accompagner d'hyperuricémie, d'hyperkaliémie, d'hypocalcémie, d'hyperphosphorémie, d'insuffisance rénale aiguë, d'augmentation des LDH, évocatrices entre autres d'un syndrome de lyse tumorale, et peut être associé à une insuffisance respiratoire aiguë et au décès. Cette insuffisance respiratoire aiguë peut être accompagnée par des événements tels qu'un infiltrat pulmonaire interstitiel ou un oedème pulmonaire visible sur une radio thoracique. Le syndrome apparaît fréquemment pendant la première ou la deuxième heure qui suit le début de la première perfusion. Les patients ayant des antécédents d'insuffisance respiratoire ou ceux avec un infiltrat pulmonaire tumoral seraient peut-être plus susceptibles de présenter des résultats moins favorables et doivent être traités avec une plus grande prudence. Chez les patients développant un syndrome grave de relargage de cytokines, la perfusion devra être arrêtée immédiatement (cf Posologie/Mode d'administration) et un traitement symptomatique drastique devra être instauré. Une amélioration rapide des symptômes pouvant être suivie d'une aggravation, ces patients doivent être étroitement surveillés jusqu'à ce que le syndrome de lyse tumorale et l'infiltrat pulmonaire aient disparu ou aient été écartés. Les patients de nouveau traités après disparition des signes et des symptômes ont rarement présenté un nouveau syndrome grave de relargage de cytokines. Les réactions liées à la perfusion, incluant le syndrome de relargage de cytokines (cf Effets indésirables), accompagnées par une hypotension et un bronchospasme ont été observées chez 10 % des patients traités par MabThera. Ces symptômes sont habituellement réversibles à l'arrêt de la perfusion de MabThera, après administration d'un antipyrétique, d'un antihistaminique et éventuellement d'oxygénothérapie, d'une réhydratation veineuse ou injection IV de bronchodilatateurs et de glucocorticoïdes, si nécessaire. Se reporter au syndrome de relargage de cytokines décrit précédemment pour les réactions sévères.

- L'administration IV de protéines peut provoquer des réactions anaphylactoïdes ou d'autres réactions d'hypersensibilité. En opposition au syndrome de relargage de cytokines, une réelle hypersensibilité apparaît habituellement dans les minutes qui suivent le début de la perfusion. Les médicaments nécessaires au traitement de telles réactions, par exemple adrénaline, antihistaminiques et glucocorticoïdes, doivent être disponibles pour un usage immédiat dans l'éventualité d'une réaction allergique pendant la perfusion de MabThera. Les manifestations cliniques de l'anaphylaxie peuvent sembler similaires aux manifestations cliniques du syndrome de relargage de cytokines (décrit ci-dessus). Les réactions attribuées à l'hypersensibilité ont été rapportées moins fréquemment que celles attribuées au relargage de cytokines.

- L'éventualité d'une hypotension pendant la perfusion de MabThera doit faire envisager l'arrêt du traitement antihypertenseur 12 heures avant la perfusion.

- Des cas d'angine de poitrine ou d'arythmie tels que flutter et fibrillations auriculaires, d'insuffisance cardiaque ou d'infarctus du myocarde ont été observés chez des patients traités par MabThera. En conséquence, les patients présentant des antécédents de pathologies cardiaques et/ou ayant reçu une chimiothérapie cardiotoxique devront être étroitement surveillés.

- Bien que MabThera en monothérapie ne soit pas myélosuppressif, il est recommandé d'être prudent quant au traitement de patients ayant un nombre de neutrophiles < 1,5 x 10^9/l et/ou un nombre de plaquettes < 75 x 10^9/l, car l'expérience clinique dans cette population est limitée. MabThera a été utilisé sans induire de myélotoxicité chez 21 patients qui avaient eu une autogreffe de moelle osseuse et chez d'autres groupes à risque avec des réserves médullaires vraisemblablement réduites.

- Lors du traitement par MabThera en monothérapie, une surveillance régulière de la numération-formule sanguine, incluant une numération plaquettaire, doit être envisagée. Lorsque MabThera est associé à une chimiothérapie « CHOP » ou « CVP », une numération-formule sanguine doit être réalisée régulièrement, conformément à la pratique médicale habituelle.

Ce document est la propriété des Centres d'Investigation Clinique. Toute reproduction, même partielle, ne peut être effectuée sans autorisation.

- De très rares cas de réactivation d'hépatite B, y compris des observations d'hépatites fulminantes, ont été rapportés chez les sujets recevant du rituximab, bien que la majorité de ces sujets ait été également exposée à des chimiothérapies cytotoxiques. Les observations comportent des facteurs de confusion dus à la fois à la maladie sous-jacente et à la chimiothérapie cytotoxique. Les patients présentant un antécédent d'hépatite B doivent être étroitement surveillés à la recherche de signes d'hépatite B active lorsqu'ils sont traités par le rituximab associé à une chimiothérapie cytotoxique.

- La tolérance d'une immunisation par un vaccin, quel qu'il soit, en particulier les vaccins viraux vivants, faisant suite à un traitement par MabThera n'a pas été étudiée. La capacité à développer une réponse humorale à la suite d'une primovaccination ou d'un rappel, quel qu'il soit, n'a pas été étudiée non plus.

Polyarthrite rhumatoïde :

Réactions liées à la perfusion :

MabThera est associé à des réactions liées à la perfusion, probablement imputables au relargage de cytokines et/ou d'autres médiateurs chimiques. La prémédication par glucocorticoïde IV a permis de réduire significativement la fréquence et la sévérité de ces événements (cf Effets indésirables).

La plupart des réactions rapportées étaient d'intensité légère à modérée. La proportion de patients affectés diminue avec le nombre de perfusions. Les réactions décrites sont habituellement réversibles après la diminution de la vitesse ou l'arrêt de la perfusion de MabThera et l'administration d'un antipyrétique, d'un antihistaminique et éventuellement d'oxygénothérapie, d'une réhydratation veineuse ou injection IV de bronchodilatateurs et de glucocorticoïdes, si nécessaire. Dans la plupart des cas, la perfusion peut reprendre à une vitesse deux fois moindre (par exemple de 100 mg/h à 50 mg/h) après disparition complète des symptômes.

L'administration IV de protéines, y compris MabThera, peut provoquer des réactions anaphylactiques ou d'autres réactions d'hypersensibilité. Les médicaments nécessaires au traitement de telles réactions, par exemple adrénaline, antihistaminiques et glucocorticoïdes, doivent être disponibles pour un usage immédiat dans l'éventualité d'une réaction allergique pendant la perfusion de MabThera. La présence d'anticorps humains antichimériques (HACA) peut être associée à une aggravation des réactions liées à la perfusion ou à des réactions allergiques suivant la seconde perfusion des traitements ultérieurs (cf Pharmacodynamie).

Dans les études cliniques, 10 patients sur 990 (1 %) atteints de polyarthrite rhumatoïde, ayant reçu une première perfusion de MabThera, ont présenté une réaction grave pendant la perfusion, quelle que soit la dose (cf Effets indésirables).

On ne dispose d'aucune donnée concernant la tolérance de MabThera chez les patients atteints d'insuffisance cardiaque modérée (NYHA classe III) ou de maladie cardiaque sévère non contrôlée. Chez les patients traités par MabThera, certaines pathologies cardiaques ischémiques préexistantes sont devenues symptomatiques, comme l'angor ; une fibrillation et un flutter auriculaires ont également été observés. Par conséquent, avant tout traitement par MabThera chez les patients ayant des antécédents cardiaques connus, il convient de prendre en considération le risque de complications cardiovasculaires résultant de réactions liées à la perfusion et d'assurer une surveillance étroite pendant l'administration. L'éventualité d'une hypotension pendant la perfusion de MabThera doit faire envisager l'arrêt du traitement antihypertenseur 12 heures avant la perfusion.

Infections :

Des infections graves, dont certaines d'issue fatale, peuvent apparaître au cours du traitement par MabThera (cf Effets indésirables). MabThera ne doit pas être administré en cas d'infection sévère et/ou évolutive (par exemple tuberculose, sepsis et infections opportunistes, cf Contre-indications) ou d'immunodépression sévère (par exemple dans les hypogammaglobulinémies ou lorsque les taux de CD4 ou CD8 sont très faibles). La prudence s'impose en cas d'utilisation de MabThera chez des patients ayant un antécédent d'infection chronique ou récidivante, ou une pathologie sous-jacente prédisposant aux infections graves (cf Effets indésirables).

L'apparition de signes d'infection après un traitement par MabThera doit motiver un examen immédiat des patients et l'instauration d'un traitement approprié. Avant d'être retraités par MabThera, les patients devront être examinés afin de rechercher un éventuel risque d'infection.

Ce document est la propriété des Centres d'Investigation Clinique. Toute reproduction, même partielle, ne peut être effectuée sans autorisation.

De très rares cas de réactivation d'une hépatite B ont été rapportés chez des patients atteints d'un lymphome non hodgkinien traités par rituximab en association à une chimiothérapie cytotoxique (voir lymphomes non hodgkiniens).
Vaccination :
On ne dispose d'aucune donnée concernant l'utilisation de vaccins chez les patients présentant une déplétion en lymphocytes B consécutive au traitement par MabThera (cf Pharmacodynamie). Chez les patients susceptibles de recevoir un traitement par MabThera, il convient donc de vérifier le statut vaccinal et de suivre les recommandations nationales relatives au bilan de vaccinations chez l'adulte. Toutes les vaccinations devront être terminées au moins 4 semaines avant la première administration de MabThera. Les vaccins vivants sont déconseillés en cas de déplétion en lymphocytes B.
Utilisation concomitante ou séquentielle d'autres traitements de fond :
L'utilisation concomitante de MabThera avec d'autres traitements de la polyarthrite rhumatoïde que ceux mentionnés dans les rubriques Indications et Posologie/Mode d'administration n'est pas recommandée.
Les données de tolérance, issues des essais cliniques, relatives à l'utilisation d'autres traitements de fond (y compris des agents biologiques) après un traitement par MabThera sont insuffisantes (cf Interactions). Les patients doivent être étroitement surveillés à la recherche de signes d'infection s'ils reçoivent des agents biologiques et/ou d'autres traitements de fond après leur traitement par MabThera.
Tumeur maligne :
Les agents immunomodulateurs peuvent augmenter le risque de tumeur maligne. Compte tenu de l'expérience limitée de MabThera chez les patients atteints de polyarthrite rhumatoïde (cf Effets indésirables), le risque potentiel de développer une tumeur solide ne peut être exclu actuellement, bien que les données disponibles ne semblent suggérer aucun risque accru.

DC INTERACTIONS (début page)

On ne dispose que de données limitées sur les interactions éventuelles de MabThera avec d'autres médicaments.
L'administration concomitante avec le méthotrexate n'a pas eu d'effet sur les paramètres pharmacocinétiques de MabThera chez les patients atteints de polyarthrite rhumatoïde. Chez les patients ayant des taux décelables d'anticorps antimurins ou d'anticorps humains antichimériques (HAMA/HACA), l'utilisation d'autres anticorps monoclonaux à des fins diagnostiques ou thérapeutiques peut provoquer des réactions d'allergie ou d'hypersensibilité. La tolérance de l'association simultanée ou séquentielle de MabThera avec des chimiothérapies, autres que « CHOP » ou « CVP », ou des agents responsables d'une déplétion en cellules B normales n'a pas été clairement établie.
Dans une petite cohorte de patients atteints de polyarthrite rhumatoïde, 110 patients ont reçu d'autres traitements de fond (y compris des agents biologiques), 4 à 6 mois après le traitement par MabThera et généralement lors de la phase de déplétion lymphocytaire B. Le taux d'infections cliniquement pertinentes a été de 7,8 pour 100 patient-années.

DC GROSSESSE et ALLAITEMENT (début page)

Grossesse :
Il n'existe pas de données suffisamment pertinentes concernant l'utilisation de MabThera chez la femme enceinte. Comme les IgG passent la barrière placentaire, le rituximab peut provoquer une déplétion des lymphocytes B chez le foetus. Pour ces raisons, l'utilisation de MabThera ne devra être envisagée au cours de la grossesse que si le bénéfice attendu paraît supérieur au risque potentiel. En raison de la présence durable du rituximab chez les patients présentant une déplétion en lymphocytes B, les femmes en âge de procréer devront utiliser des mesures contraceptives efficaces tout au long du traitement par MabThera et pendant 12 mois après son arrêt. Les études de toxicité effectuées chez le singe cynomolgus n'ont pas révélé d'embryotoxicité in utero. La descendance de femelles exposées à MabThera présentait une déplétion en lymphocytes B pendant la phase post-natale. Dans l'espèce humaine, aucune étude clinique n'a mesuré les taux de lymphocytes B chez le nouveau-né après exposition maternelle à MabThera.
Allaitement :
Le passage du rituximab dans le lait maternel n'est pas connu. Cependant, les IgG maternelles passent dans le lait, et le rituximab a été détecté dans le lait maternel chez le singe. En conséquence, les femmes ne doivent pas allaiter pendant le traitement par MabThera et pendant 12 mois suivant son arrêt.

Ce document est la propriété des Centres d'Investigation Clinique. Toute reproduction, même partielle, ne peut être effectuée sans autorisation.

DC CONDUITE et UTILISATION DE MACHINES (début page)

Aucune étude des effets de MabThera sur l'aptitude à conduire ou à utiliser des machines n'a été réalisée, mais les données actuelles sur l'activité pharmacologique et sur les événements indésirables ne paraissent pas indiquer d'effets de cette nature.

DC EFFETS INDÉSIRABLES (début page)

Lymphomes non hodgkiniens :

Lymphome folliculaire non hodgkinien :

- Monothérapie :
  Les données suivantes sont issues de 356 patients traités par MabThera en monothérapie dans des études à un seul bras (cf Pharmacodynamie). La plupart des patients ont reçu 375 mg/m^2 de MabThera par semaine pendant 4 semaines. Ce chiffre inclut 39 patients ayant une masse tumorale importante (lésions >= 10 cm) et 58 patients ayant reçu plus d'une cure de MabThera (60 reprises du traitement). 37 patients ont reçu 375 mg/m^2 pendant 8 semaines et 25 patients ont reçu des doses différentes de 375 mg/m^2 pendant 4 semaines et jusqu'à 500 mg/m^2 en dose unique dans l'étude de phase 1.
  Le tableau suivant décrit les événements indésirables qui étaient considérés comme au moins possiblement liés à MabThera pendant les 12 mois qui suivaient le traitement. Les événements indésirables ont été cotés selon les quatre grades des critères de toxicité courants de l'Institut national de cancérologie (NCI).

Tableau 1 : Résumé des événements indésirables rapportés chez >= 1 % des 356 patients atteints de lymphome non hodgkinien ayant reçu MabThera en monothérapie dans les essais cliniques :

Appareil<br>Effet indésirable	Tous les grades<br>%	Grades 3 et 4<br>%
Tout événement indésirable	91,0	17,7
Tolérance générale :		
Fièvre	48,3	0,6
Frisson	31,7	2,2
Asthénie	18,0	0,3
Céphalées	12,6	0,6
Irritation laryngée	7,6	-
Douleurs abdominales	7,0	0,6
Douleurs dorsales	4,5	0,3
Douleurs	4,2	-
Bouffées vasomotrices	4,2	-
Douleurs thoraciques	2,2	-
Malaise	2,0	-
Douleurs d'origine tumorale	1,7	-
Rhume	1,4	-
Douleurs cervicales	1,1	-
Appareil cardiovasculaire :		
Hypotension artérielle	9,8	0,8
Hypertension artérielle	4,5	0,3
Tachycardie	1,4	-
Arythmie	1,4	0,6
Hypotension orthostatique	1,1	-

Ce document est la propriété des Centres d'Investigation Clinique. Toute reproduction, même partielle, ne peut être effectuée sans autorisation.

Appareil digestif :		
Nausées	17,1	0,3
Vomissements	6,7	0,3
Diarrhée	4,2	-
Dyspepsie	2,8	-
Anorexie	2,8	-
Dysphagie	1,4	0,3
Stomatite	1,4	-
Constipation	1,1	-
Organes hématopoïétiques :		
Leucopénie	12,4	2,8
Neutropénie	11,2	4,2
Thrombopénie	9,6	1,7
Anémie	3,7	1,1
Troubles du métabolisme et de la nutrition :		
Angio-œdème	10,7	0,3
Hyperglycémie	5,3	0,3
OEdèmes périphériques	4,8	-
Augmentation des LDH	2,2	-
Hypocalcémie	2,2	-
Oedème de la face	1,1	-
Amaigrissement	1,1	-
Appareil musculosquelettique :		
Myalgies	8,1	0,3
Arthralgies	5,9	0,6
Hypertonie	1,4	-
Douleurs	1,1	0,3
Système nerveux :		
Vertiges	7,3	-
Paresthésies	2,5	-
Anxiété	2,2	-
Insomnie	2,2	-
Vasodilatation	1,7	-
Hypoesthésie	1,4	-
Agitation	1,4	-
Appareil respiratoire :		
Bronchospasme	7,9	1,4
Rhinite	7,3	0,3
Toux accrue	5,1	0,3
Dyspnée	2,2	0,8
Douleur thoracique	1,1	-
Maladie respiratoire	1,1	-
Peau et annexes :		

Ce document est la propriété des Centres d'Investigation Clinique. Toute reproduction, même partielle, ne peut être effectuée sans autorisation.

Prurit	12,4	0,3
Rash	11,2	0,3
Urticaire	7,3	0,8
Sueurs nocturnes	2,8	-
Transpiration	2,8	-
Organes des sens :		
Larmoiement	3,1	-
Conjonctivite	1,4	-
Otalgie	1,1	-
Acouphènes	1,1	-

- Les événements indésirables suivants ont également été rapportés (< 1 %) : troubles de la coagulation, asthme, trouble pulmonaire, bronchiolite obstructive, hypoxie, dilatation abdominale, douleur au point d'injection, bradycardie, lymphadénopathie, nervosité, dépression, dysgueusie.

    - Réactions liées à la perfusion : les réactions liées à la perfusion sont apparues chez plus de 50 % des patients et principalement lors de la première perfusion, habituellement au cours des deux premières heures. Ces événements comprenaient principalement fièvre, frissons et tremblements. D'autres symptômes tels que rougeurs, angio-oedème, nausées, rash/urticaire, fatigue, céphalées, irritation laryngée, rhinite, vomissements et douleurs au niveau de la tumeur sont apparus. Dans environ 10 % des cas, ces symptômes étaient accompagnés d'hypotension et de bronchospasme. Il a également été observé, moins fréquemment, une exacerbation de pathologies cardiaques préexistantes comme l'angor ou l'insuffisance cardiaque congestive. L'incidence des symptômes liés à la perfusion décroît considérablement au cours des perfusions ultérieures (cf Mises en garde/Précautions d'emploi).

    - Infections : MabThera a induit une déplétion en lymphocytes B chez 70 à 80 % des patients, mais il n'a été associé à une diminution des immunoglobulines sériques que chez un faible nombre de patients. Des événements infectieux, indépendamment de toute évaluation du lien de causalité, sont survenus chez 30,3 % des 356 patients : 18,8 % des patients ont présenté une infection bactérienne, 10,4 % une infection virale, 1,4 % une infection fongique et 5,9 % une infection d'étiologie inconnue. Des événements infectieux sévères (grade 3 ou 4), incluant des septicémies, sont survenus chez 3,9 % des patients : chez 1,4 % pendant la période de traitement et chez 2,5 % pendant la période de suivi. S'agissant d'études ne comportant qu'un seul bras, la responsabilité de MabThera ou du LNH sous-jacent et de son traitement antérieur dans le développement de ces infections n'a pas pu être déterminée.

    - Effets indésirables hématologiques : des anomalies hématologiques, habituellement discrètes et réversibles sont survenues chez une minorité de patients. Une thrombopénie et une neutropénie sévères (grades 3 et 4) ont été rapportées respectivement chez 1,7 % et 4,2 % des patients, et une anémie sévère a été rapportée chez 1,1 %. Un seul cas d'aplasie médullaire transitoire (érythroblastopénie) et quelques rares cas d'anémie hémolytique ont été rapportés après traitement par MabThera.

    - Événements cardiovasculaires : des événements cardiovasculaires ont été rapportés chez 18,8 % des patients au cours du traitement. Les événements le plus fréquemment rapportés ont été une hypotension et une hypertension artérielle. Deux patients (0,6 %) ont présenté un trouble du rythme de grade 3 ou 4 (tachycardies ventriculaire et supraventriculaire) pendant une perfusion de MabThera et un patient avec des antécédents d'infarctus du myocarde a

Ce document est la propriété des Centres d'Investigation Clinique. Toute reproduction, même partielle, ne peut être effectuée sans autorisation.

présenté un angor qui a évolué vers un infarctus du myocarde quatre jours plus tard.

- Populations particulières :

  - Patient âgé (>= 65 ans) : l'incidence de tous les événements indésirables ainsi que des événements indésirables de grades 3 et 4 a été comparable chez les patients âgés (N = 94) et chez les patients plus jeunes (N = 237) : 88,3 % versus 92,0 % pour tous les événements indésirables et 16,0 % versus 18,1 % pour les événements indésirables de grades 3 et 4.

  - Masse tumorale importante : l'incidence des événements indésirables de grades 3 et 4 a été plus élevée chez les patients présentant une masse tumorale importante (N = 39) que chez les patients n'ayant pas de masse tumorale importante (N = 195) : 25,6 % versus 15,4 %. L'incidence de tous les événements indésirables a été comparable dans les deux groupes (92,3 % dans le groupe masse tumorale importante versus 89,2 % dans le groupe n'ayant pas de masse tumorale importante).

  - Retraitement : dans la population retraitée par MabThera (N = 60), le pourcentage de patients ayant rapporté au moins un événement indésirable, y compris les événements indésirables de grades 3 et 4, a été comparable à celui observé dans la population de patients (N = 203) traitée une première fois (95,0 % versus 89,7 % pour tous les événements indésirables et 13,3 % versus 14,8 % pour les événements indésirables de grades 3 et 4).

- Événements indésirables rapportés au cours d'autres études cliniques en monothérapie : un cas de maladie sérique a été rapporté au cours d'une étude clinique utilisant MabThera en monothérapie pour le traitement d'un lymphome diffus à grandes cellules B.

- En association à une chimiothérapie « CVP » : Les données suivantes sont issues d'une étude clinique randomisée de phase III, menée sur 321 patients, comparant l'association MabThera plus « CVP » (R-CVP) à « CVP » seul (162 R-CVP, 159 « CVP »).
  Les différences entre les groupes traités en terme de type et d'incidence des événements indésirables s'expliquent principalement par les évènements indésirables caractéristiques de MabThera en monothérapie. Les événements indésirables de grades 3 et 4 suivants ont été rapportés avec une incidence >= 2 % dans le bras R-CVP par rapport au bras « CVP » et pourraient de ce fait être imputables au protocole R-CVP.
  Les événements indésirables ont été classés selon l'échelle à quatre grades des critères de toxicité courants de l'Institut national de cancérologie (NCI) : Fatigue : 3,7 % (R-CVP) ; 1,3 % (CVP). Neutropénie : 3,1 % (R-CVP) ; 0,6 % (CVP).

  - Réactions liées à la perfusion : les signes et les symptômes des réactions sévères ou menaçant le pronostic vital (grades 3 et 4 du NCI-CTC) liées à la perfusion (définies comme apparaissant pendant la perfusion de MabThera ou dans un délai d'un jour après celle-ci) sont survenues chez 9 % de tous les patients qui ont reçu R-CVP. Ces résultats sont comparables à ceux observés en monothérapie (cf Mises en garde/Précautions d'emploi, Effets indésirables : Monothérapie) et incluaient frissons, fatigue, dyspnée, dyspepsie, nausée, rash (sans autre précision), bouffée vasomotrice.

  - Infections :
    La proportion de patients présentant des infections ou des infestations pendant le traitement ou une période de 28 jours après la fin du traitement de l'étude, a été globalement comparable entre les deux bras (33 % R-CVP, 32 % « CVP »). Les infections les plus fréquentes ont été les infections respiratoires hautes rapportées chez 12,3 % des patients du bras R-CVP et

Ce document est la propriété des Centres d'Investigation Clinique. Toute reproduction, même partielle, ne peut être effectuée sans autorisation.

chez 16,4 % des patients du bras « CVP » ; la plupart de ces infections étaient des rhinopharyngites. Des infections sévères ont été rapportées chez 4,3 % des patients recevant R-CVP et chez 4,4 % des patients recevant « CVP ». Aucune infection menaçant le pronostic vital n'a été rapportée pendant cette étude.

- Anomalies biologiques hématologiques : 24 % des patients du bras R-CVP et 14 % des patients du bras « CVP » ont présenté une neutropénie de grade 3 ou 4 pendant le traitement. La proportion de patients présentant une neutropénie de grade 4 était comparable dans les deux groupes. Ces anomalies biologiques ont été rapportées en tant qu'événements indésirables et ont nécessité une intervention médicale chez 3,1 % des patients du bras R-CVP et chez 0,6 % des patients du bras « CVP ». Les autres anomalies biologiques n'ont pas été traitées et se sont spontanément résolues. De plus, l'incidence plus élevée des neutropénies du bras R-CVP n'a pas été associée à une augmentation de l'incidence des infections et infestations. Aucune différence significative entre les deux bras de traitement n'a été observée en ce qui concerne l'anémie (0,6 % avec R-CVP et 1,9 % avec le « CVP ») et la thrombopénie (1,2 % avec R-CVP et aucun cas rapporté avec « CVP ») de grades 3 et 4.

- Événements cardiaques : l'incidence globale des troubles cardiaques dans la population analysée pour la tolérance a été faible (4 % R-CVP, 5 % « CVP »), sans différence significative entre les deux groupes traités.

- Traitement d'entretien : Les données suivantes sont issues d'une étude clinique de phase III où des patients présentant un lymphome folliculaire non hodgkinien en rechute ou réfractaire ont été randomisés dans une première phase, entre un traitement d'induction par « CHOP » (cyclophosphamide, doxorubicine, vincristine, prednisone) et l'association MabThera plus « CHOP » (R-CHOP). Dans une seconde phase, les patients ayant répondu au traitement d'induction par « CHOP » ou R-« CHOP » ont été randomisés entre ne plus recevoir de traitement supplémentaire (observation) et un traitement d'entretien par MabThera. Le traitement d'entretien par MabThera consistait en une perfusion unique de MabThera à la posologie de 375 mg/m^2 de surface corporelle tous les trois mois pendant une durée maximale de 2 ans ou jusqu'à progression de la maladie. Dans la phase d'induction de l'étude, la tolérance des deux protocoles d'induction a été évaluée au total chez 462 patients (228 « CHOP », 234 R-CHOP).

Tableau 2 : Phase d'induction : résumé des événements indésirables de grade 3 et 4 rapportés chez >= 2 % des 462 patients atteints de lymphome non hodgkinien dans l'un ou l'autre groupe (« CHOP » ou R-CHOP) :

MedDRA Classe de systèmes d'organes<br>Événement indésirable	CHOP<br>N = 228	R-CHOP<br>N = 234
	Incidence N (%)	
Tout événement indésirable	152 (67)	185 (79)
Affections gastro-intestinales :		
Nausées*	9 (4)	13 (6)
Vomissements	8 (4)	7 (3)
Douleurs abdominales	6 (3)	4 (2)
Diarrhée	5 (2)	6 (3)
Constipation*	1 (< 1)	7 (3)
Stomatite	1 (< 1)	4 (2)
Affections hématologiques et du système lymphatique :		
Neutropénie*	108 (47)	129 (55)

Ce document est la propriété des Centres d'Investigation Clinique. Toute reproduction, même partielle, ne peut être effectuée sans autorisation.

Leucopénie	106 (46)	111 (47)
Thrombopénie	18 (8)	17 (7)
Neutropénie fébrile*	8 (4)	14 (6)
Hématotoxicité	12 (5)	9 (4)
Anémie	5 (2)	6 (3)
Troubles généraux et anomalies au site d'administration :		
Asthénie	10 (4)	5 (2)
Pyrexie	6 (3)	7 (3)
Troubles du système nerveux :		
Troubles sensoriels	4 (2)	7 (3)
Affections de la peau et du tissu sous-cutané :		
Alopécie*	15 (7)	30 (13)
Affection cutanée	2 (< 1)	4 (2)
Infections et infestations :		
Infection neutropénique	18 (8)	15 (6)
Sepsis	5 (2)	3 (1)
Infection urinaire	4 (2)	3 (1)
Affections respiratoires, thoraciques et médiastinales :		
Dyspnée	6 (3)	3 (1)
Affections musculosquelettiques et systémiques :		
Douleurs dorsales	1 (<1)	4 (2)
Troubles du métabolisme et de la nutrition :		
Hyperglycémie	5 (2)	4 (2)
Affections du système immunitaire :		
Hypersensibilité*	-	10 (4)
Affections cardiaques :		
Trouble cardiaque	6 (3)	2 (< 1)

- * Événements indésirables rapportés avec une incidence plus élevée (différence >= 2 %) dans le groupe R-CHOP que dans le groupe « CHOP » et pouvant donc être imputables à MabThera. 332 patients (166 observation, 166 rituximab) ont été inclus pour l'évaluation de la tolérance de la phase d'entretien de l'étude. MabThera a été administré à la dose de 375 mg/m^2 de surface corporelle une fois tous les trois mois jusqu'à progression de la maladie ou pendant une durée maximale de deux ans.

Tableau 3 : Phase d'entretien : résumé des événements indésirables de grade 3 et 4 rapportés chez >= 2 % des 332 patients atteints de lymphome non hodgkinien dans l'un ou l'autre groupe (observation ou MabThera en entretien) :

MedDRA Classe de systèmes d'organes Événement indésirable	Observation N = 166	MabThera N = 166
	Incidence N (%)	
Tout événement indésirable	38 (23)	61 (37)
Troubles généraux et anomalies au site d'administration :		
Asthénie	4 (2)	1 (< 1)

Ce document est la propriété des Centres d'Investigation Clinique. Toute reproduction, même partielle, ne peut être effectuée sans autorisation.

Infections et infestations :		
Pneumonie	1 (< 1)	3 (2)
Infection de l'appareil respiratoire*	-	3 (2)
Affections hématologiques et du système lymphatique :		
Neutropénie*	7 (4)	17 (10)
Leucopénie*	4 (2)	8 (5)
Hématotoxicité	3 (2)	2 (1)
Affections du système nerveux :		
Troubles sensoriels	2 (1)	3 (2)
Affections de la peau et du tissu sous-cutané :		
Alopécie*	-	3 (2)
Affections vasculaires :		
Hypertension	2 (1)	3 (2)
Affections cardiaques :		
Trouble cardiaque*	4 (2)	6 (4)

- * Événements indésirables rapportés avec une incidence plus élevée (différence >= 2%) dans le groupe MabThera que dans le groupe observation et pouvant donc être imputables à MabThera.

  - Réactions liées à la perfusion : au cours du traitement d'entretien, des signes et symptômes non graves évoquant une réaction liée à la perfusion ont été rapportés chez 41 % des patients dans la classe des troubles généraux (essentiellement asthénie, pyrexie, syndrome pseudogrippal, douleur) et chez 7 % des patients dans la classe des troubles du système immunitaire (hypersensibilité). Des réactions graves liées à la perfusion (définies comme étant des événements indésirables graves survenus le jour de la perfusion de rituximab ou dans les 24 heures suivant celle-ci) sont survenues chez moins de 1 % des patients recevant le traitement d'entretien par MabThera.

  - Infections : la proportion des patients ayant présenté des infections de grade 1 à 4 était de 25 % dans le groupe observation et de 45 % dans le groupe MabThera, celle des patients ayant présenté des infections de grade 3-4 ayant été respectivement de 3 % et 11 %. Les infections de grade 3 à 4 rapportées chez >= 1 % des patients du groupe MabThera étaient : pneumonie (2 %), infection de l'appareil respiratoire (2 %), infection fébrile (1 %) et zona (1 %). Dans la majorité des infections (tous grades), l'agent infectieux n'a pas été spécifié ou isolé. Toutefois, quand un agent infectieux a été spécifié, il s'agissait le plus souvent d'une bactérie (observation 2 %, MabThera 10 %), d'un virus (observation 7 %, MabThera 11 %) ou d'un champignon (observation 2 %, MabThera 4 %). Aucune toxicité cumulative en termes d'infections n'a été rapportée sur les 2 ans de la période d'entretien.

  - Événements hématologiques : des leucopénies (tous grades) sont survenues chez 21 % des patients du groupe observation contre 29 % des patients du groupe MabThera, et une neutropénie a été rapportée chez respectivement 12 % et 23 % des patients. L'incidence des neutropénies (observation 4 %, MabThera 10 %) et des leucopénies (observation 2 %, MabThera 5 %) de grade 3-4 a été plus élevée dans le groupe MabThera que dans le groupe observation. L'incidence des thrombopénies de grade 3 à 4 était faible (observation 1 %, MabThera < 1 %).

  - Affections cardiaques : l'incidence des affections cardiaques de grade 3 à 4 était comparable entre les deux groupes de traitement (observation 4 %,

Ce document est la propriété des Centres d'Investigation Clinique. Toute reproduction, même partielle, ne peut être effectuée sans autorisation.

MabThera 5 %). Des événements cardiaques ont été rapportés à titre d'événements indésirables graves chez moins de 1 % des patients du groupe observation et 3 % de ceux du groupe MabThera : fibrillation auriculaire (1 %), infarctus du myocarde (1 %), insuffisance ventriculaire gauche (< 1 %), ischémie myocardique (< 1 %).

- Taux sériques des IgG : après le traitement d'induction, les taux sériques médians des IgG étaient en deçà de la limite inférieure de la normale (< 7 g/l) dans les groupes observation et MabThera. Dans le groupe observation, le taux sérique médian des IgG a ensuite augmenté au-dessus de la limite inférieure de la normale, mais est demeuré constant dans le groupe MabThera. Pendant les 2 ans de traitement, la proportion des patients ayant présenté un taux sérique d'IgG inférieur à la limite inférieure de la normale était d'environ 60 % dans le groupe MabThera tandis qu'elle a diminué dans le groupe observation (36 % après 2 ans).

Lymphome non hodgkinien agressif diffus à grandes cellules B :
En association à une chimiothérapie « CHOP » :
Le tableau suivant présente les événements indésirables cliniques de grades 3 à 4 et les infections de grade 2, survenus au cours d'une étude clinique randomisée de phase III, comparant l'association MabThera plus « CHOP » (R-CHOP) à « CHOP » seul, sur une population analysée pour la tolérance de 398 patients. Les événements décrits ont été rapportés avec une incidence supérieure à 2 % dans le bras R-CHOP par rapport au bras « CHOP » seul et pourrait, de ce fait, être imputable au protocole R-CHOP (seuil d'incidence supérieur à 2 %). Les événements indésirables ont été classés selon l'échelle à quatre grades des critères de toxicité courants du National Cancer Institute of Canada (NCIC).

Tableau 4 : Incidence supérieure (>= 2 %) des événements indésirables de grades 3 et 4 (incluant les infections de grade 2) du bras R-CHOP comparé au bras « CHOP » (seuil supérieur à 2 %) chez les patients atteints d'un lymphome non hodgkinien :

	R-CHOP N = 202	CHOP N = 196
	%	%
Infections et infestations :		
Bronchite	11,9	8,2
Zona	4,0	1,5
Bronchite aiguë	2,5	0,5
Sinusite	2,5	-
Troubles respiratoires :		
Dyspnée	8,9	3,6
Troubles généraux et accidents liés au site d'administration :		
Tremblements	3,5	1,0
Troubles vasculaires :		
Hypertension	2,5	0,5
Troubles cardiaques :		
Fibrillation auriculaire	2,5	0,5

- Réactions liées à la perfusion : des réactions de grade 3 ou 4 liées à la perfusion (définies comme apparaissant pendant la perfusion de MabThera ou dans un délai d'un jour après celle-ci) sont survenues chez environ 9 % des patients lors de la première cure de R-CHOP. L'incidence des réactions de grades 3 et 4 liées à la perfusion a diminué pour atteindre moins de 1 % lors de la huitième cure de R-CHOP. Les signes et symptômes étaient analogues à ceux observés sous traitement en monothérapie (cf Mises en garde/Précautions d'emploi, Effets indésirables : Monothérapie) et incluaient : fièvre, frissons, hypotension, hypertension, tachycardie, dyspnée, bronchospasme, nausées, vomissements, douleurs et éléments caractéristiques du syndrome de lyse tumorale. D'autres réactions ont été rapportées

Ce document est la propriété des Centres d'Investigation Clinique. Toute reproduction, même partielle, ne peut être effectuée sans autorisation.

de façon isolée au moment du traitement par R-CHOP : infarctus du myocarde, fibrillation auriculaire et oedème pulmonaire.

- Infections : le pourcentage de patients ayant présenté une infection de grade 2 à 4 et/ou une neutropénie fébrile a été de 55,4 % dans le groupe R-CHOP et de 51,5 % dans le groupe « CHOP ». Les neutropénies fébriles (c'est-à-dire sans rapport d'infection documentée concomitante) ont été rapportées uniquement pendant le traitement avec une incidence de 20,8 % dans le groupe R-CHOP et de 15,3 % dans le groupe « CHOP ». L'incidence globale des infections de grade 2 à 4 a été de 45,5 % dans le groupe R-CHOP et de 42,3 % dans le groupe « CHOP », sans différence en ce qui concerne l'incidence des infections bactériennes et fongiques systémiques. Les infections fongiques de grade 2 à 4 étaient plus fréquentes dans le groupe R-CHOP (4,5 % contre 2,6 % dans le groupe « CHOP ») ; cette différence était due à une plus grande incidence d'infections localisées à candida pendant la période de traitement. L'incidence du zona de grade 2 à 4, incluant le zona ophtalmique, était plus élevée dans le groupe R-CHOP (4,5 %) que dans le groupe « CHOP » (1,5 %), avec 7 cas sur un total de 9 dans le groupe R-CHOP, survenant pendant le traitement.

- Événements hématologiques : après chaque cure, une leucopénie (88 % contre 79 %) et une neutropénie (97 % contre 88 %) de grade 3 à 4 sont survenues plus fréquemment dans le groupe R-CHOP que dans le groupe « CHOP ». Il n'y avait pas de preuve que les neutropénies étaient plus prolongées dans le groupe R-CHOP. Aucune différence entre les deux groupes traités n'a été observée en ce qui concerne l'anémie (19 % avec le « CHOP » contre 14 % avec le R-CHOP) et la thrombopénie (16 % avec le « CHOP » contre 15 % avec le R-CHOP) de grade 3 à 4. Les anomalies hématologiques se sont normalisées dans des délais comparables pour les deux groupes traités.

- Événements cardiaques : l'incidence des arythmies de grade 3 à 4, principalement des arythmies supraventriculaires telles que tachycardie et flutter/fibrillation auriculaire, a été plus élevée dans le groupe R-CHOP (14 patients, 6,9 %) que dans le groupe « CHOP » (3 patients, 1,5 %). Toutes ces arythmies sont survenues pendant la perfusion de MabThera ou étaient associées à des facteurs de prédispositions tels que fièvre, infection, infarctus aigu du myocarde, ou à des pathologies respiratoires et cardiovasculaires préexistantes. Aucune différence entre les groupes R-CHOP et « CHOP » n'a été observée en ce qui concerne l'incidence des autres événements cardiaques de grade 3 à 4 incluant : insuffisance cardiaque, myocardiopathie et manifestations de coronaropathie.

- Événements neurologiques : pendant la période de traitement, 4 patients (2 %) du groupe R-CHOP ayant tous des facteurs de risque cardiovasculaire ont présenté des accidents vasculaires cérébraux thromboemboliques pendant le premier cycle de traitement. Aucune différence n'a été observée entre les deux groupes en ce qui concerne l'incidence des autres événements thromboemboliques. Au contraire, 3 patients (1,5 %) du groupe « CHOP » ont présentés des événements vasculaires cérébraux qui se sont tous produits pendant la période de suivi.

Polyarthrite rhumatoïde :

L'efficacité clinique de MabThera, administré en association au méthotrexate (MTX), a été étudiée dans trois études cliniques contrôlées en double insu (une étude de phase III et deux de phase II) chez des patients atteints de polyarthrite rhumatoïde. Plus de 1000 patients ont reçu au moins un traitement et ont été suivis pendant des périodes allant de 6 mois à plus de 3 ans ; près de 600 patients ont reçu au moins deux traitements pendant la période de suivi.

Les patients ont reçu 2 x 1000 mg de MabThera à deux semaines d'intervalle, ainsi que du méthotrexate (10-25 mg/semaine). Les perfusions de MabThera ont été administrées après une perfusion IV de 100 mg de méthylprednisolone ; la majorité des patients a également reçu un traitement par prednisone orale pendant 15 jours. Le tableau 5 présente les événements indésirables rapportés par au moins 1 % des patients et plus fréquemment chez les patients ayant reçu au moins une perfusion de MabThera que chez les patients sous placebo, dans l'essai de phase III et dans l'ensemble des patients des études de phase II.

Les événements indésirables les plus fréquents considérés comme liés à l'administration de 2 x 1000 mg de MabThera dans les études de phase II et III ont été les réactions aiguës liées à

Ce document est la propriété des Centres d'Investigation Clinique. Toute reproduction, même partielle, ne peut être effectuée sans autorisation.

la perfusion. Ce type de réactions est apparu chez 15 % des patients après la première perfusion de rituximab et 5 % des patients sous placebo. Après la deuxième perfusion, la fréquence des réactions a diminué à 2 % dans les groupes rituximab et placebo.

Tableau 5 : Événements indésirables liés au traitement survenant chez au moins 1 % des patients atteints de polyarthrite rhumatoïde et plus fréquemment chez les patients sous MabThera pendant les études cliniques en insu de phase II et III :

	Ensemble de la population des études de phase II		Population de l'étude de phase III	
	MTX + placebo N = 189 n (%)	Rituximab + MTX N = 232 n (%)	MTX + placebo N = 209 n (%)	Rituximab + MTX N = 308 n (%)
Réactions aiguës liées à la perfusion :*				
Hypertension	10 (5 %)	22 (9 %)	11 (5 %)	21 (7 %)
Nausées	14 (7 %)	19 (8 %)	5 (2 %)	22 (7 %)
Rash	6 (3 %)	18 (8 %)	9 (4 %)	17 (6 %)
Pyrexie	1 (< 1 %)	12 (5 %)	7 (3 %)	15 (5 %)
Prurit	1 (< 1 %)	14 (6 %)	4 (2 %)	12 (4 %)
Urticaire	0	2 (< 1 %)	3 (< 1 %)	10 (3 %)
Rhinite	2 (1 %)	6 (3 %)	4 (2 %)	8 (3 %)
Irritation laryngée	0	5 (2 %)	0	6 (2 %)
Bouffées vasomotrices	4 (2 %)	2 (< 1 %)	0	6 (2 %)
Hypotension artérielle	11 (6 %)	10 (4 %)	1 (< 1 %)	5 (2 %)
Frisson	3 (2 %)	13 (6 %)	6 (3 %)	3 (< 1 %)
Infections et infestations :				
Tout type d'infection	56 (30 %)	85 (37 %)	78 (37 %)	127 (41 %)
Infections urinaires	8 (4 %)	14 (6 %)	17 (8 %)	15 (5 %)
Infections respiratoires hautes	28 (15 %)	31 (13 %)	26 (12 %)	48 (16 %)
Infections respiratoires basses/pneumonie	10 (5 %)	9 (4 %)	5 (2 %)	8 (3 %)
Troubles généraux :				
Asthénie	0	3 (1 %)	1 (< 1 %)	6 (2 %)
Affections gastro- intestinales :				
Dyspepsie	3 (2 %)	9 (4 %)	0	7 (2 %)
Douleur abdominale haute	3 (2 %)	7 (3 %)	1 (< 1 %)	4 (1 %)
Troubles du métabolisme et de la nutrition :				
Hypercholestérolémie	1 (< 1 %)	3 (1 %)	0	6 (2 %)
Affections musculo-squelettiques :				
Arthralgie/douleurs ostéomusculaires	8 (4 %)	18 (7 %)	6 (3 %)	17 (7 %)
Spasmes musculaires	0	1 (< 1 %)	2 (1 %)	7 (2 %)
Arthrose	1 (< 1 %)	4 (2 %)	0	6 (2 %)
Système nerveux :				
Paresthésies	2 (1 %)	4 (2 %)	1 (< 1 %)	8 (3 %)

Tearsnonsurligné30-10-07
Ce document est la propriété des Centres d'Investigation Clinique. Toute reproduction, même partielle, ne peut être effectuée sans autorisation.

Migraine	0	4 (2 %)	2 (1 %)	5 (2 %)

* Réactions survenant dans les 24 heures suivant la perfusion.

Outre les événements présentés dans le tableau ci-dessus, des événements médicalement significatifs, considérés comme potentiellement liés au traitement, ont été rapportés peu fréquemment chez les patients sous rituximab :

- Troubles généraux : oedème généralisé.
- Troubles respiratoires : bronchospasme, sifflements respiratoires, oedème laryngé.
- Affections de la peau et du tissu sous-cutané : oedème de Quincke, prurit généralisé.
- Affections du système immunitaire : anaphylaxie, réactions anaphylactoïdes.

Retraitement :

Les données cliniques limitées sur le retraitement des patients atteints de polyarthrite rhumatoïde sont en faveur d'un même profil de tolérance que celui observé après le traitement initial. Cependant, au vu des données disponibles chez les patients présentant des HACA après des traitements répétés par rituximab, on ne peut exclure la possibilité d'aggravation des réactions liées à la perfusion ou de réactions allergiques, ou d'un échec de déplétion lymphocytaire B. Les réactions aiguës liées à la perfusion étaient généralement plus fréquentes après la première perfusion de MabThera que lors des traitements ultérieurs.

Réactions aiguës liées à la perfusion :

Des symptômes évocateurs d'une réaction aiguë liée à la perfusion (prurit, fièvre, urticaire/rash, frissons, pyrexie, tremblements, éternuements, oedème de Quincke, irritation laryngée, toux et bronchospasme, avec ou sans hypotension ou hypertension associée) ont été observés chez 79/540 (15 %) patients après la première exposition à MabThera. Dans une étude comparant l'effet des glucocorticoïdes, ces événements ont été observés chez 5/149 (3 %) des patients après la première perfusion de placebo et 42/192 (22 %) des patients recevant la première perfusion de 1000 mg de rituximab. La prémédication par glucocorticoïde IV a permis de réduire significativement la fréquence et la sévérité de ces événements. Parmi les patients ayant reçu 1000 mg de rituximab sans prémédication par glucocorticoïdes, 18/65 (28 %) ont présenté une réaction aiguë liée à la perfusion, contre 24/127 (19 %) des patients ayant reçu une prémédication par glucocorticoïde IV.

Infections :

Le taux d'infection a été d'environ 0,9 patient-année sous MabThera. Les infections étaient principalement des infections respiratoires hautes et des infections urinaires. L'incidence des infections cliniquement significatives, dont certaines ont été fatales, a été de 0,05 patient-année dans le groupe MabThera.

Tumeurs malignes :

Les données cliniques, en particulier le nombre de retraitements par patient, sont trop limitées pour évaluer l'incidence de tumeurs malignes après exposition au rituximab, bien que les données disponibles ne semblent suggérer aucun risque accru. L'évaluation de la tolérance à long terme est en cours.

Événements cardiovasculaires :

Des événements cardiaques ont été observés chez 11 % des patients traités par MabThera dans les études cliniques. Dans les études contrôlées versus placebo, les événements cardiaques graves ont été rapportés à fréquence égale (2 %) chez les patients des groupes MabThera et placebo.

Expérience depuis la commercialisation :

Dans le cadre de la surveillance de la tolérance de MabThera, après sa mise sur le marché, les réactions indésirables graves suivantes ont été observées :

Tableau 6 : Événements indésirables graves observés depuis la commercialisation :	
Organes hématopoiétiques :	
- Rare (>= 1/10 000, < 1/1000)	Neutropénies retardées[(1)]
- Très rare (< 1/10 000)	Pancytopénie<br>Aplasie médullaire<br>Augmentation transitoire des taux sériques d'IgM[(2)]
Appareil cardiovasculaire :	

Ce document est la propriété des Centres d'Investigation Clinique. Toute reproduction, même partielle, ne peut être effectuée sans autorisation.

- Rare (>= 1/10 000, < 1/1000)	Événements cardiaques sévères(3)(4)
- Très rare (< 1/10 000)	Insuffisance cardiaque(3)(4)<br>Infarctus du myocarde(3)(4)
Affections de l'oreille et du labyrinthe :	
- Très rare (< 1/10 000)	Perte d'audition(5)
Affections oculaires :	
- Très rare (< 1/10 000)	Perte importante de la vision(5)
Troubles généraux et anomalie au site d'administration :	
- Très rare (< 1/10 000)	Défaillance multiviscérale(3)
Affections du système immunitaire :	
- Peu fréquent (>= 1/1000, < 1/100)	Réactions liées à la perfusion
- Rare (>= 1/10 000, < 1/1000)	Anaphylaxie
- Très rare (< 1/10 000)	Syndrome de lyse tumorale(3)<br>Syndrome de relargage des cytokines(3)<br>Maladie sérique<br>Réactivation d'hépatite B(6)
Affections du système nerveux :	
- Très rare (< 1/10 000)	Neuropathie crânienne<br>Neuropathie périphérique<br>Paralysie faciale(5)<br>Perte d'autres sens(5)
Affections du rein et des voies urinaires :	
- Très rare (< 1/10 000)	Insuffisance rénale(3)
Affections respiratoires, thoraciques et médiastinales :	
- Rare (>= 1/10 000, < 1/1000)	Bronchospasme(3)
- Très rare (< 1/10 000)	Insuffisance respiratoire(3)<br>Infiltrats pulmonaires<br>Pneumonies interstitielles
Affections de la peau et du tissu sous-cutané :	
- Très rare (< 1/10 000)	Réactions cutanées bulleuses sévères<br>Nécrolyses épidermiques toxiques(7)
Affections vasculaires :	
- Très rare (< 1/10 000)	Vascularites (principalement cutanées)<br>Vascularite leucocytoclasique

(1) Neutropénies qui sont apparues plus de 4 semaines après la dernière perfusion de MabThera.

(2) Dans les études conduites avec le rituximab depuis sa commercialisation chez les patients ayant une macroglobulinémie de Waldenstrom, des augmentations transitoires des taux sériques d'IgM, qui peuvent s'accompagner d'un syndrome d'hyperviscosité, ont été observées après l'initiation du traitement. L'augmentation des IgM est transitoire et revient habituellement à sa valeur initiale dans les 4 mois.

(3) Associés aux réactions liées à la perfusion. Des cas fatals ont rarement été rapportés.

(4) Observés principalement chez les patients présentant des antécédents cardiaques et/ou ayant reçu une chimiothérapie cardiotoxique et qui étaient le plus souvent associés aux réactions liées à la perfusion.

(5) Signes et symptômes de la neuropathie crânienne. Apparus dans des délais variables, jusqu'à plusieurs mois après la fin du traitement par MabThera.

(6) De très rares cas de réactivation d'hépatite B ont été rapportés, dont la majorité est survenue chez les sujets traités par rituximab en association à une chimiothérapie cytotoxique.

Ce document est la propriété des Centres d'Investigation Clinique. Toute reproduction, même partielle, ne peut être effectuée sans autorisation.

(7) Incluant des cas fatals.

DC SURDOSAGE (début page)

Au cours des essais cliniques chez l'homme, aucun cas de surdosage n'a été observé. Le produit n'a pas été testé à des doses uniques supérieures à 1000 mg au cours d'études cliniques contrôlées.

PP PHARMACODYNAMIE (début page)

Groupe pharmacothérapeutique : agent antinéoplasique (code ATC : L01XC02). Le rituximab se lie spécifiquement à l'antigène transmembranaire CD20, une phosphoprotéine non glycosylée située sur les lymphocytes pré-B et B matures. Cet antigène s'exprime dans plus de 95 % des cellules B des lymphomes non hodgkiniens (LNH). Il est présent sur les cellules B normales et malignes, mais ne l'est pas sur les cellules souches hématopoïétiques, les cellules pro-B, les plasmocytes normaux et les autres tissus normaux. Cet antigène ne s'internalise pas lors de la liaison à l'anticorps et il n'est pas libéré de la surface cellulaire. Le CD20 ne circule pas sous forme libre dans le plasma et n'entre donc pas en compétition pour la liaison à l'anticorps. Le fragment Fab du rituximab se lie à l'antigène CD20 des lymphocytes B et le fragment Fc peut générer des fonctions d'effecteurs immunitaires qui entraînent la lyse de ces lymphocytes. Les mécanismes possibles de la lyse cellulaire induite par les effecteurs sont une cytotoxicité dépendante du complément (CDC), faisant intervenir la liaison du fragment C1q, et une cytotoxicité cellulaire dépendante des anticorps (ADCC), passant par un ou plusieurs des récepteurs Fcgamma de la surface des granulocytes, des macrophages et des cellules NK. Il a aussi été démontré que le rituximab, en se liant à l'antigène CD20 des lymphocytes B, induit une mort cellulaire par apoptose. Le nombre des lymphocytes B périphériques a chuté au-dessous de la normale après l'administration de la première dose de MabThera. Chez les patients traités pour une hémopathie maligne, la reconstitution lymphocytaire B a débuté dans les 6 mois suivant l'instauration du traitement et s'est normalisée entre 9 et 12 mois après la fin de celui-ci. Chez les patients atteints de polyarthrite rhumatoïde, une déplétion immédiate en lymphocytes B périphériques a été observée après deux perfusions de 1000 mg de MabThera effectuées à 14 jours d'intervalle. Les numérations de lymphocytes B périphériques commencent à augmenter dès la 24e semaine et la majorité des patients présente des signes de repopulation à la 40e semaine, que MabThera soit administré en monothérapie ou en association au méthotrexate.

Expérience clinique dans les lymphomes non hodgkiniens :

Lymphome folliculaire non hodgkinien :

- Monothérapie :

  - Traitement initial, administration une fois par semaine pendant 4 semaines : Dans l'étude pivotale, 166 patients présentant un lymphome folliculaire non hodgkinien à cellules B ou un lymphome de bas grade, en rechute ou chimiorésistant, ont reçu 375 mg/m^2 de MabThera par perfusion intraveineuse une fois par semaine pendant quatre semaines. Le taux de réponse globale dans la population en intention de traiter (ITT) a été de 48 % ($IC_{95\ \%}$ 41 % - 56 %) avec 6 % de réponse complète (RC) et 42 % de réponse partielle (RP). Le temps médian jusqu'à progression de la maladie (TTP) pour les patients répondeurs a été de 13,0 mois. Dans une analyse en sous-groupe, le taux de réponse globale a été plus élevé chez les patients appartenant aux sous-types histologiques B, C et D (International Working Formulation) par comparaison au sous-type A (58 % versus 12 %). Le taux de réponse globale a été plus élevé chez les patients dont la lésion la plus importante mesurait moins de 5 cm dans son plus grand diamètre, comparé aux patients présentant des lésions supérieures à 7 cm (53 % versus 38 %). Enfin, ce taux a été plus élevé chez les patients présentant une rechute chimiosensible comparé à celui obtenu chez les patients présentant une rechute chimiorésistante, définie par une durée de réponse < 3 mois (50 % versus 22 %). Chez les patients traités auparavant par greffe autologue de moelle osseuse (AGMO), le taux de réponse globale a été de 78 % versus 43 % chez les patients n'ayant pas bénéficié d'une AGMO. Parmi les variables suivantes : l'âge, le sexe, le grade du lymphome, le diagnostic initial, la présence ou non de masse tumorale importante, l'élévation ou non des LDH, la présence d'une atteinte extraganglionnaire, aucune n'a eu d'effet statistiquement significatif (test exact de Fisher) sur la réponse au traitement par MabThera. Une corrélation statistiquement significative a été établie entre

Ce document est la propriété des Centres d'Investigation Clinique. Toute reproduction, même partielle, ne peut être effectuée sans autorisation.

les taux de réponse et l'atteinte médullaire. 40 % des patients présentant une atteinte médullaire ont répondu, contre 59 % pour les patients n'en présentant pas (p = 0,0186). Cette relation n'a pas été confirmée par une analyse de régression logistique progressive dans laquelle les facteurs suivants ont été identifiés comme étant des facteurs pronostiques : type histologique, bcl-2 positif à l'inclusion, résistance à la dernière chimiothérapie et masse tumorale importante.

- Traitement initial, administration une fois par semaine pendant 8 semaines : Dans une étude multicentrique à un seul bras, 37 patients présentant un lymphome folliculaire non hodgkinien à cellules B ou un lymphome de bas grade, en rechute ou chimiorésistant, ont reçu 375 mg/m^2 de MabThera en perfusion intraveineuse une fois par semaine pendant 8 semaines. Le taux de réponse global a été de 57 % ($IC_{95\,\%}$ 41 % - 73 % ; RC 14 %, RP 43%), avec un TTP médian pour les patients répondeurs de 19,4 mois (valeurs extrêmes : 5,3 à 38,9 mois).

- Traitement initial, masse tumorale importante, administration une fois par semaine pendant 4 semaines : Dans les données poolées de trois études, 39 patients ayant une masse tumorale importante (lésion >= 10 cm de diamètre), un lymphome folliculaire non hodgkinien à cellules B ou un lymphome de bas grade, en rechute ou chimiorésistant, ont reçu une dose de MabThera de 375 mg/m^2 en perfusion IV, une fois par semaine pendant 4 semaines. Le taux de réponse global a été de 36 % ($IC_{95\,\%}$ 21 % - 51 % ; RC 3 %, RP 33 %), avec un TTP médian pour les patients répondeurs de 9,6 mois (valeurs extrêmes : 4,5 à 26,8 mois).

- Retraitement, administration une fois par semaine pendant 4 semaines : Dans une étude multicentrique à un seul bras, 58 patients ayant une masse tumorale importante, un lymphome folliculaire non hodgkinien à cellules B ou un lymphome de bas grade, en rechute ou chimiorésistant, qui avaient présenté une réponse clinique objective à une cure précédente de MabThera, ont été retraités avec une dose de 375 mg/m^2 de MabThera en perfusion IV, une fois par semaine pendant 4 semaines. 3 de ces patients avaient déjà reçu deux cures de MabThera avant inclusion et ont donc reçu une troisième cure au cours de l'étude. 2 des patients ont été re-traités à deux reprises pendant l'étude. En ce qui concerne les 60 reprises de traitement effectuées au cours de l'étude, le taux de réponse global a été de 38 % ($IC_{95\,\%}$ 26 % - 51 % ; RC 10 %, RP 28 %), avec un TTP médian pour les patients répondeurs de 17,8 mois (valeurs extrêmes : 5,4 à 26,6 mois). Ce résultat se compare favorablement au TTP obtenu après la première cure de MabThera (12,4 mois).

- Traitement initial, en association à « CVP » : Dans une étude ouverte randomisée, un total de 322 patients, non traités précédemment, présentant un lymphome non hodgkinien folliculaire à cellules B ou de bas grade, a été randomisé pour recevoir soit une chimiothérapie « CVP » (cyclophosphamide 750 mg/m^2 le jour 1, vincristine 1,4 mg/m^2 jusqu'à un maximum de 2 mg le jour 1, et prednisolone 40 mg/m^2/jour les jours 1-5) toutes les 3 semaines pendant 8 cures, soit 375 mg/m^2 de MabThera associé à « CVP » (R-CVP). MabThera a été administré le premier jour de chaque cure. Un total de 321 patients (162 R-CVP, 159 « CVP ») ont reçu le traitement et ont été analysés en terme d'efficacité. Au moment de l'analyse finale, le temps médian d'observation était de 18 mois. R-CVP a conduit a un bénéfice significatif par rapport à « CVP » pour le critère principal, le temps jusqu'à échec du traitement (25,9 mois contre 6,7 mois, p < 0,0001, log-rank test). Le risque de survenue d'un événement conduisant à l'échec du traitement a été réduit de 67 % ($IC_{95\,\%}$: 56 %-76 %) dans le bras R-CVP comparé au bras « CVP » seul (analyse de régression selon le modèle de Cox). Le taux estimé de survie sans événement (Kaplan-Meier) à 12 mois était de 69 % dans le bras R-CVP versus 32 % dans le bras « CVP ». La proportion de patients présentant une réponse tumorale (RC, RC non confirmée, RP) était significativement supérieure (p < 0,0001, test du chi 2) dans le bras R-CVP (80,9 %) par rapport au bras « CVP » (57,2 %). A 18 mois, la durée médiane de réponse au

Ce document est la propriété des Centres d'Investigation Clinique. Toute reproduction, même partielle, ne peut être effectuée sans autorisation.

traitement n'avait pas encore été atteinte dans le bras R-CVP et était de 9,8 mois dans le bras « CVP » ($p < 0{,}0001$, test log-rank). Chez les patients répondeurs, le risque de rechute a été réduit de 70 % ($IC_{95\,\%}$: 55 %-81 %) dans le bras R-CVP par rapport au bras « CVP » (analyse de régression selon le modèle de Cox). Le temps au bout duquel un nouveau traitement du lymphome a été nécessaire, ou le temps au bout duquel le patient est décédé était significativement plus long dans le bras R-CVP (non évaluable) par rapport au bras « CVP » (12,3 mois ; $p < 0{,}0001$, test log-rank). Le traitement par R-CVP a prolongé de façon significative le temps jusqu'à progression de la maladie ou le décès par rapport au traitement par « CVP », 27 mois et 14,5 mois respectivement. A 12 mois, 81 % des patients du bras R-CVP n'avaient pas rechuté comparé à 58 % des patients du bras « CVP ». Une analyse postérieure, effectuée avec une durée médiane d'observation d'environ 42 mois du critère principal et de tous les critères secondaires, a confirmé la supériorité du bénéfice de R-CVP par rapport à « CVP » (tableau 7).

Tableau 7 : Résumé des résultats actualisés de l'efficacité de « CVP » vs R-CVP (durée médiane d'observation : 42 mois) :

	Estimation du délai médian sans événement (mois) (Kaplan-Meier) (1)			Effets du traitement(2)
	CVP	R-CVP	Valeur de p (log-rank)	
Durée médiane d'observation (mois)	41,3	42,1		
Critère principal d'efficacité				
- Temps jusqu'à échec du traitement (TTF)	6,6	27,0	< 0,0001	66 %
Critères secondaires d'efficacité				
- Temps jusqu'à progression de la maladie ou décès(3)	14,5	33,6	< 0,0001	58 %
- Survie globale	NA	NA	0,0700	38 %
- Réponse tumorale globale (RC, RCn, RP)(4)	57 %	81 %	< 0,0001(5)	3,2(6)
- Durée de réponse	13,5	37,7	< 0,0001	65 %
- Survie sans maladie	20,5	44,8	0,0005	71 %
- Temps jusqu'à nouveau traitement du lymphome ou décès	12,3	46,3	< 0,0001	63 %

- (1) Selon l'évaluation de l'investigateur, toutes données stratifiées par centre. (2) Effet du traitement : pour les paramètres sans événement, les estimations étaient calculées en termes de réduction du risque ; l'odds ratio a été utilisé pour la réponse tumorale. NA : non atteint, car les estimations du taux d'absence d'événement (Kaplan-Meier) étaient supérieures à 50 % pendant la totalité de la période d'observation de l'étude. (3) Défini comme critère secondaire d'efficacité dans l'amendement G du protocole. (4) Le taux de réponse globale est calculé sur la base de la réponse tumorale évaluée à la fin du traitement à l'étude. (5) Test du Chi^2. (6) Odds ratio. Abréviations : NA : non atteint ; TTF (Time to Treatment Failure) : temps jusqu'à échec du traitement ; RC : réponse complète ; RCn : réponse complète non confirmée ; RP : réponse partielle. Le taux de décès de cause spécifique (décès dus au lymphome) était significativement plus bas dans le bras R-CVP que dans le bras « CVP » ($p = 0{,}02$ avec stratification par centre, test log-rank ; taux sans événement à 3 ans : 93 % pour le R-CVP vs 8 % pour le « CVP »). Le bénéfice de l'association du rituximab à « CVP » a été systématiquement observé dans la population de l'essai M 39021 (randomisation selon le critère du BNLI [non versus oui], l'âge [<= 60 ans, > 60 ans], le nombre de localisations extraganglionnaires [0-1 versus > 1], l'envahissement de la moelle osseuse [non versus oui], le taux de LDH [élevé, non

Ce document est la propriété des Centres d'Investigation Clinique. Toute reproduction, même partielle, ne peut être effectuée sans autorisation.

élevé], le taux de ß2-microglobuline [élevée, non élevée], les symptômes B [absents, présents], la masse tumorale importante [absente, présente], le nombre de localisations ganglionnaires [< 5 versus >= 5], l'hémoglobine [<= 12 g/dl versus > 12 g/dl], l'IPI [International Prognostic Index ; <= 1 versus > 1] et l'index FLIPI (Follicular Lymphoma International Prognostic Index ; 0-2 versus 3-5]).

- Traitement d'entretien : Dans une étude internationale multicentrique prospective ouverte de phase III, 465 patients présentant un lymphome folliculaire non hodgkinien en rechute ou réfractaire ont été randomisés dans une première étape entre un traitement d'induction par « CHOP » (cyclophosphamide, doxorubicine, vincristine, prednisone ; n = 231) et l'association MabThera plus « CHOP » (R-CHOP ; n = 234). Les deux groupes de traitement étaient bien équilibrés en terme de caractéristiques initiales des patients et de statut de la maladie. Dans une seconde étape, 334 patients ayant présenté une rémission complète ou partielle à la suite du traitement d'induction ont été randomisés entre un traitement d'entretien par MabThera (n = 167) et l'observation (n = 167). Le traitement d'entretien par MabThera consistait en une perfusion de 375 mg/m^2 de surface corporelle tous les trois mois jusqu'à progression de la maladie ou pendant une durée maximale de deux ans. L'analyse finale de l'efficacité a inclus tous les patients randomisés dans chacune des deux parties de l'étude. Après une durée médiane d'observation de 31 mois des patients randomisés dans la phase d'induction, le R-CHOP a significativement amélioré l'évolution des LNH folliculaires en rechute ou réfractaires comparativement au « CHOP » (voir tableau 8).

Tableau 8 : Phase d'induction : résumé des résultats sur l'efficacité de « CHOP » versus R-CHOP (durée médiane d'observation : 31 mois) :

	CHOP	R-CHOP	Valeur de p	Réduction du risque[(1)]
Critère principal d'efficacité				
- Taux de réponse globale[(2)]	74 %	87 %	0,0003	ND
- Réponse complète (RC)[(2)]	16 %	29 %	0,0005	ND
- Réponse partielle (RP)[(2)]	58 %	58 %	0,9449	ND

- [(1)] Les estimations ont été calculées par la méthode des hazard ratios. [(2)] Dernière réponse tumorale évaluée par l'investigateur. Le test statistique « principal » pour la « réponse » a été le trend test RC versus RP versus absence de réponse (p < 0,0001). Abréviations : ND : non disponible ; NA : non atteint ; RC : réponse complète ; RP : réponse partielle ; SG : survie globale ; SSP : survie sans progression.
  Chez les patients randomisés lors de la phase d'entretien de l'étude, la durée médiane d'observation a été de 28 mois à partir de la randomisation. Le traitement d'entretien par MabThera a amélioré de façon cliniquement et statistiquement significative le critère principal, la SSP (délai entre la randomisation pour le traitement d'entretien et la rechute, la progression de la maladie ou le décès) comparativement à l'observation seule (p < 0,0001, test log-rank). La SSP médiane a été de 42,2 mois dans le bras entretien par MabThera comparé à 14,3 mois dans le bras observation. Le risque de progression de la maladie ou de décès (régression selon le modèle de Cox) était réduit de 61 % avec le traitement d'entretien par MabThera comparativement à l'observation (IC95 % : 45 %-72 %). Le taux de survie sans progression (Kaplan-Meier) à 12 mois était de 78 % dans le groupe traitement d'entretien par MabThera contre 57 % dans le groupe observation. Une analyse de la survie globale a confirmé le bénéfice significatif du traitement d'entretien par MabThera par rapport à l'observation (p = 0,0039, test log-rank). Le traitement d'entretien par MabThera a réduit le risque de décès de 56 % (IC95 % : 22 %-75 %).

Tableau 9 : Phase d'entretien : résumé des résultats d'efficacité de MabThera vs observation (durée médiane d'observation : 28 mois) :

Ce document est la propriété des Centres d'Investigation Clinique. Toute reproduction, même partielle, ne peut être effectuée sans autorisation.

Paramètres d'efficacité	Délai médian jusqu'à l'événement (mois) (Kaplan-Meier)			Réduction du risque
	Observation (N = 167)	MabThera (N = 167)	Valeur de p (log-rank)	
Survie sans progression (SSP)	14,3	42,2	< 0,0001	61 %
Survie globale	NA	NA	0,0039	56 %
Temps jusqu'à nouveau traitement du lymphome	20,1	38,8	< 0,0001	50 %
Survie sans maladie[1]	16,5	53,7	0,0003	67 %
Analyse en sous-groupes				
SSP				
- CHOP	11,6	37,5	< 0,0001	71 %
- R-CHOP	22,1	51,9	0,0071	46 %
- RC	14,3	52,8	0,0008	64 %
- RP	14,3	37,8	< 0,0001	54 %
SG				
- CHOP	NA	NA	0,0348	55 %
- R-CHOP	NA	NA	0,0482	56 %

- [1] Uniquement applicable aux patients obtenant une RC.
  NA : non atteinte.
  Le bénéfice du traitement d'entretien par MabThera a été confirmé dans tous les sous-groupes analysés quel que soit le protocole d'induction (« CHOP » ou R-CHOP) ou la qualité de la réponse au traitement d'induction (RC ou RP) ; tableau 9. Le traitement d'entretien par MabThera a significativement prolongé la SSP des patients ayant répondu au traitement d'induction par « CHOP » (SSP médiane : 37,5 mois vs 11,6 mois, p < 0,0001) ou par R-CHOP (SSP médiane : 51,9 mois vs 22,1 mois, p = 0,0071). Malgré des effectifs réduits dans les sous-groupes, le traitement d'entretien par MabThera a conféré un bénéfice significatif de survie globale, tant chez les patients répondeurs à « CHOP » qu'à R-CHOP, mais un suivi plus long est nécessaire afin de confirmer cette observation.

Lymphome non hodgkinien agressif diffus à grandes cellules B :
Dans une étude randomisée, ouverte, 399 patients âgés (de 60 à 80 ans), naïfs, présentant un lymphome agressif diffus à grandes cellules B, ont reçu soit une chimiothérapie « CHOP » standard (cyclophosphamide 750 mg/m^2, doxorubicine 50 mg/m^2, vincristine 1,4 mg/m^2 jusqu'à un maximum de 2 mg le jour 1, et prednisolone 40 mg/m^2/jour les jours 1 à 5) toutes les 3 semaines pour 8 cycles, soit une chimiothérapie « CHOP » associée à MabThera 375 mg/m^2 (R-CHOP). MabThera a été administré le premier jour de chaque cycle.
L'analyse finale d'efficacité a inclus tous les patients randomisés (197 « CHOP », 202 R-CHOP), avec une durée médiane de suivi d'environ 31 mois. A l'inclusion, les deux groupes de patients traités étaient bien équilibrés quant aux caractéristiques et l'état de la maladie. L'analyse finale a confirmé que le protocole R-CHOP était associé à une amélioration de la survie sans événement (critère principal d'efficacité : les événements étaient le décès, la rechute ou la progression du lymphome, ou l'instauration d'un nouveau traitement antilymphome) cliniquement et statistiquement significatifs (p = 0,0001). Les estimations de la durée médiane de la survie sans événement étaient de 35 mois (Kaplan-Meier) dans le bras R-CHOP, contre 13 mois dans le bras « CHOP », représentant une diminution du risque de 41 %. A 24 mois, l'estimation de la survie globale était de 68,2 % dans le bras R-CHOP contre 57,4 % dans le bras « CHOP ». Une analyse ultérieure de la durée globale de survie, avec une durée médiane de suivi de 60 mois, a confirmé le bénéfice du protocole R-CHOP par rapport au protocole « CHOP » (p = 0,0071), représentant une réduction du risque de 32 %.
L'analyse de tous les objectifs secondaires (taux de réponse, survie sans progression, survie sans maladie, durée de la réponse) a confirmé les effets du protocole R-CHOP par rapport au

Ce document est la propriété des Centres d'Investigation Clinique. Toute reproduction, même partielle, ne peut être effectuée sans autorisation.

protocole « CHOP ». Le taux de réponse complète après 8 cycles de R-CHOP était de 76,2 % contre 62,4 % dans le groupe « CHOP » (p = 0,0028). Le risque de progression de la maladie était réduit de 46 % et le risque de rechute de 51 %. Dans tous les sous-groupes de patients (sexe, âge, IPI ajusté à l'âge, stade de Ann Arbor, ECOG, bêta-2 microglobuline, LDH, albumine, symptômes B, masse tumorale importante, sites extranodaux, envahissement de la moelle osseuse), les risques ratios de la survie sans événement et de la survie globale (du protocole R-CHOP comparé au protocole « CHOP ») étaient respectivement inférieurs à 0,83 et 0,95. Le protocole R-CHOP était associé à une amélioration de l'évolution à la fois chez les patients à haut et à faible risque selon l'IPI ajusté à l'âge.

Résultats biologiques :

Sur 67 patients chez lesquels ont été recherchés des anticorps humains antimurins (HAMA), aucun cas n'a été trouvé positif. Sur 356 recherches d'anticorps HACA, moins de 1,1 % (4 patients) étaient positives.

Expérience clinique dans la polyarthrite rhumatoïde :

L'efficacité et la tolérance de MabThera dans la réduction des signes et symptômes de la polyarthrite rhumatoïde ont été démontrées dans trois études multicentriques, randomisées, contrôlées et en double insu.

L'étude 1 était une étude comparative en double insu incluant 517 patients ayant présenté une réponse inadéquate ou une intolérance à un ou plusieurs traitements par anti-TNF. Les patients éligibles étaient atteints de polyarthrite rhumatoïde active depuis au moins 6 mois, diagnostiquée selon les critères de l'ACR (American Collège of Rheumatology), avec au moins 8 articulations gonflées sur 66 et au moins 8 articulations douloureuses sur 68, et une élévation de la protéine C réactive ou de la vitesse de sédimentation. Le critère principal de jugement était le pourcentage de patients ayant obtenu une réponse ACR20 à la 24e semaine. Les patients ont reçu deux perfusions IV de 1000 mg de MabThera, à 15 jours d'intervalle. Ces perfusions étaient précédées chacune d'une perfusion IV de 100 mg de méthylprednisolone. Tous les patients ont reçu un traitement concomitant par méthotrexate oral (10-25 mg/semaine) et 60 mg de prednisone orale les jours 2 à 7, ainsi que 30 mg les jours 8 à 14 après la première perfusion.

L'étude 2 était une étude randomisée et contrôlée, multifactorielle 3 x 3, menée en double insu et double placebo, qui comparait deux doses de rituximab administrées avec ou sans une ou deux perfusions de corticoïdes en association à du méthotrexate hebdomadaire chez des patients atteints de polyarthrite rhumatoïde active n'ayant pas répondu à au moins 5 autres DMARDs.

L'étude 3 était une étude contrôlée en double insu et double placebo, évaluant le rituximab en monothérapie ou associé au cyclophosphamide ou au méthotrexate chez des patients atteints de polyarthrite rhumatoïde active n'ayant pas répondu à un ou plusieurs traitements antérieurs par DMARDs.

Le groupe de comparaison dans ces trois études était le groupe sous méthotrexate hebdomadaire (10- 25 mg par semaine).

Résultats d'efficacité clinique :

Dans les trois études, le rituximab (2 x 1000 mg) a significativement augmenté la proportion de patients présentant une amélioration d'au moins 20 % du score ACR comparativement aux patients traités par le méthotrexate seul (tableau 10). L'effet du traitement était similaire chez tous les patients, indépendamment du taux de facteur rhumatoïde, de l'âge, du sexe, de la surface corporelle, de la race, du nombre de traitements antérieurs ou de l'évolution de la maladie. Une amélioration cliniquement et statistiquement significative de tous les paramètres de la réponse ACR (nombres d'articulations gonflées et douloureuses, évaluations globales par le patient et par le médecin, indice d'incapacité [HAQ], évaluation de la douleur et CRP [mg/dl]) a également été observée.

Tableau 10 : Comparaison des réponses ACR obtenues à la 24e semaine dans les différentes études (population ITT) :

	Réponse ACR	Placebo + MTX	Rituximab + MTX
Étude 1		N = 201	N = 298
	ACR20	36 (18 %)	153 (51 %)$^{(1)}$
	ACR50	11 (5 %)	80 (27 %)$^{(1)}$
	ACR70	3 (1 %)	37 (12 %)$^{(1)}$
Étude 2		N = 143	N = 185
	ACR20	45 (31 %)	96 (52 %)$^{(2)}$

Ce document est la propriété des Centres d'Investigation Clinique. Toute reproduction, même partielle, ne peut être effectuée sans autorisation.

	ACR50	19 (13 %)	61 (33 %)[(2)]
	ACR70	6 (4 %)	28 (15 %)[(2)]
Étude 3		N = 40	N = 40
	ACR20	15 (38 %)	28 (70 %)[(3)]
	ACR50	5 (13 %)	17 (43 %)[(3)]
	ACR70	2 (5 %)	9 (23 %)[(3)]

[(1)] p <= 0,0001
[(2)] p <= 0,001
[(3)] p <= 0,05

Dans l'étude 3, la réponse ACR20 des patients traités par le rituximab seul était de 65 %, contre 38 % sous méthotrexate seul (p = 0,025).

La diminution du score d'activité de la maladie (DAS28) a été significativement plus importante sous rituximab que sous méthotrexate seul (variation moyenne du DAS28 par rapport à la valeur initiale respectivement de -1,9 vs -0,4, p < 0,0001). La proportion de patients obtenant une réponse EULAR bonne ou modérée a été significativement plus élevée sous rituximab que sous méthotrexate seul (tableau 11).

Résultats sur la qualité de vie :

Sous rituximab ont été observées des diminutions significatives de l'indice d'incapacité (HAQ-DI), de la fatigue (FACIT-F) ; tableau 11, ainsi qu'une amélioration des scores physique et mental du SF-36, comparativement au méthotrexate seul (SF-36 physique : 5,8 vs 0,9, SF-36 mental : 4,7 vs 1,3, respectivement, étude 1).

Tableau 11 : Résultats d'efficacité clinique et de qualité de vie à la 24e semaine de l'étude 1 :

Réponse à la semaine 24 : Variations par rapport aux valeurs initiales	Placebo + MTX1[(1)] N = 201 moyenne (écart type)	Rituximab + MTX1[(1)] N = 298 moyenne (écart type)	valeur p
EULAR, bonne/modérée	22 %	65 %	
HAQ[(2)]	-0,1 (0,5)	-0,4 (0,6)	< 0,0001
FACIT-F[(3)]	-0,5 (9,8)	-9,1 (11,3)	< 0,0001

[(1)] Méthotrexate.
[(2)] HAQ : Health assessment questionnaire (questionnaire d'évaluation de l'état de santé).
[(3)] FACIT-F : Functional assessment of chronic illness therapy (évaluation fonctionnelle du traitement de maladies chroniques).

A la 24e semaine, dans les trois études, la proportion de patients présentant une amélioration cliniquement significative du score HAQ-DI (définie comme une diminution > 0,25 du score total individuel) a été plus élevée sous rituximab que sous méthotrexate seul.

Analyses biologiques :

Au total, 96 patients sur 1039 (9,2 %) atteints de polyarthrite rhumatoïde ont eu des taux décelables d'anticorps humains antichimériques dans les études cliniques, après un traitement par MabThera. L'émergence des anticorps humains antichimériques (HACA) n'a pas été associée à une détérioration clinique ou à un risque accru de réactions aux perfusions ultérieures chez ces patients. La présence d'HACA peut être associée à une aggravation des réactions liées à la perfusion ou à des réactions allergiques suivant la seconde perfusion des traitements ultérieurs. Chez un patient présentant des HACA, un échec de déplétion lymphocytaire B a été observé après plusieurs traitements.

Chez 675 patients inclus dans les études cliniques, les variations suivantes de taux d'anticorps antinucléaires (ANA) avant et après rituximab ont été observées : 26 % de patients séronégatifs ANA sont devenus positifs et 32 % de patients séropositifs ANA sont devenus négatifs. Il n'y a eu aucun signe d'apparition de nouvelle maladie auto-immune.

Lorsque la recherche de facteur rhumatoïde était initialement positive (patients FR-positifs), l'administration de rituximab s'est accompagnée d'une diminution importante des concentrations de facteur rhumatoïde dans les trois études (45-64 %).

Une hyperuricémie (grade 3/4) est apparue chez 143/950 (15 %) patients, essentiellement à J1 et/ou J15 après la perfusion. Elle n'a pas été associée à des symptômes cliniques et aucun de ces patients n'a présenté des signes de pathologie rénale.

Les concentrations plasmatiques d'immunoglobulines totales, les numérations lymphocytaires totales et le nombre de globules blancs sont restés généralement dans les limites de la normale après un traitement par MabThera, à l'exception d'une diminution transitoire du

Ce document est la propriété des Centres d'Investigation Clinique. Toute reproduction, même partielle, ne peut être effectuée sans autorisation.

nombre de globules blancs pendant les quatre premières semaines suivant le traitement. Les titres des anticorps IgG spécifiques aux antigènes des oreillons, rubéole, varicelle, anatoxine tétanique, grippe et pneumocoques sont restés stables pendant les 24 premières semaines suivant l'exposition à MabThera chez les patients atteints de polyarthrite rhumatoïde.

Retraitement :

A la fin de l'étude comparative de 24 semaines avec traitement en double insu, les patients ont été autorisés à entrer dans une étude de suivi à long terme ouverte. Au cours de cette étude, les patients ont été, si nécessaire, retraités par MabThera sur décision de l'investigateur en fonction de l'activité de la maladie, et sans tenir compte du taux de lymphocytes B périphériques. L'intervalle entre les traitements était variable, et la majorité des patients ont été retraités 6 à 12 mois après le traitement précédent. Cet intervalle a été plus long chez certains patients. La réponse au retraitement a été au moins équivalente à celle du traitement initial, comme le montre la variation du DAS28 par rapport à la valeur initiale (variation moyenne du DAS28 par rapport à la valeur initiale : traitement initial -2,18, deuxième traitement -2,75).

PP PHARMACOCINÉTIQUE (début page)

Lymphomes non hodgkiniens :

Des études de pharmacocinétique, réalisées au cours d'une étude de phase I dans laquelle des patients (n = 15) porteurs d'un lymphome à lymphocytes B récidivant ont reçu des doses uniques de rituximab de 10, 50, 100 ou 500 mg/m^2, ont indiqué que la concentration sérique et la demi-vie du rituximab étaient proportionnelles à la dose. Dans une cohorte de 14 patients sur les 166 porteurs d'un lymphome non hodgkinien folliculaire ou de faible grade récidivant ou chimiorésistant inclus dans l'essai pivotal de phase III et ayant reçu le rituximab à la dose de 375 mg/m^2 en perfusion IV, une fois par semaine pendant quatre semaines, la demi-vie sérique moyenne a été de 76,3 heures (valeurs extrêmes : 31,5 à 152,6 heures) après la première perfusion et de 205,8 heures (valeurs extrêmes : 83,9 à 407,0 heures) après la quatrième perfusion. La Cmax moyenne après la première et la quatrième perfusion a été respectivement de 205,6 µg/ml ± 59,9 et de 464,7 µg/ml ± 119,0. La clairance sérique moyenne après la première et la quatrième perfusion a été respectivement de 0,0382 l/h ± 0,0182 et de 0,0092 l/h ± 0,0033.

Néanmoins, la variabilité des taux sériques était importante.

Les concentrations sériques du rituximab sont plus élevées de façon statistiquement significative chez les patients répondeurs comparés aux patients non répondeurs au traitement, juste avant et après la quatrième perfusion et après le traitement. Les concentrations sériques sont inversement proportionnelles à la masse tumorale et à la valeur initiale du nombre de lymphocytes B circulants. En général, le rituximab reste décelable pendant 3 à 6 mois.

L'élimination et la distribution n'ont pas été étudiées de façon approfondie chez des patients atteints de lymphome non hodgkinien agressif diffus à grandes cellules B, mais les données disponibles indiquent que les concentrations sériques du rituximab chez ces patients sont comparables à celles obtenues chez des patients atteints de lymphome folliculaire non hodgkinien après traitement à des doses similaires.

Polyarthrite rhumatoïde :

Après deux perfusions IV de 1000 mg de MabThera effectuées à 14 jours d'intervalle, la demi-vie terminale moyenne était de 20,8 jours (8,58 à 35,9 jours), la clairance systémique moyenne était de 0,23 l/jour (0,091 à 0,67 l/jour), et le volume de distribution moyen à l'état d'équilibre était de 4,6 l (1,7 à 7,51 l). L'analyse de pharmacocinétique de population effectuée sur les mêmes données a conduit à des valeurs moyennes similaires pour la clairance systémique et la demi-vie, c'est-à-dire respectivement 0,26 l/jour et 20,4 jours. Cette analyse a également révélé que la surface corporelle et le sexe constituaient les covariables les plus importantes pour expliquer la variabilité interindividuelle des paramètres pharmacocinétiques. Après ajustement par rapport à la surface corporelle, les sujets de sexe masculin avaient un volume de distribution plus important et une clairance plus rapide que les sujets de sexe féminin. Ces différences pharmacocinétiques liées au sexe ne sont pas considérées comme cliniquement significatives et ne nécessitent donc aucune adaptation posologique. Après l'administration intraveineuse de doses de 500 et 1000 mg de rituximab à deux reprises, à deux semaines d'intervalle, les valeurs moyennes de Cmax étaient respectivement de 183 µg/ml (81,8 à 279 µg/ml) et 370 µg/ml (212 à 637 µg/ml), et les demi-vies moyennes de 17,9 jours (12,3 à 31,3 jours) et 19,7 jours (12,3 à 34,6 jours). On ne dispose d'aucune donnée pharmacocinétique concernant les patients atteints d'insuffisance hépatique ou rénale

Ce document est la propriété des Centres d'Investigation Clinique. Toute reproduction, même partielle, ne peut être effectuée sans autorisation.

ou les patients recevant plusieurs cures de traitement. Après le même schéma posologique (2 × 1000 mg, IV, à 2 semaines d'intervalle), les paramètres pharmacocinétiques des patients ayant une réponse inadéquate aux anti-TNF étaient comparables, avec une concentration sérique maximale moyenne de 369 µg/ml et une demi-vie terminale moyenne de 19,2 jours.

PP SÉCURITE PRÉCLINIQUE (début page)

Le rituximab s'est révélé hautement spécifique de l'antigène CD20 des lymphocytes B. Les études de toxicité chez le singe cynomolgus n'ont révélé aucun autre effet que la diminution pharmacologique prévue du nombre des lymphocytes B dans le sang circulant et les tissus lymphoïdes. Des études de toxicité sur le développement ont été effectuées chez le singe cynomolgus à des doses atteignant 100 mg/kg (traitement les 20 à 50e jour de la gestation) et n'ont révélé aucune foetotoxicité imputable au rituximab. On a cependant observé, dans les organes lymphoïdes des foetus, une déplétion pharmacologique en lymphocytes B dépendant de la dose ; cette déplétion persistait après la naissance et s'accompagnait d'une diminution des taux d'IgG chez le nouveau-né animal. Chez ces animaux, les numérations des lymphocytes B se sont normalisées dans les 6 mois suivant la naissance et n'ont pas interféré sur la réaction à la vaccination. Il n'a pas été effectué d'étude chez l'animal pour déterminer le pouvoir carcinogène du rituximab ou pour préciser ses effets sur la fertilité chez le mâle ou la femelle. Les tests standards de recherche du pouvoir mutagène n'ont pas été effectués, car ils ne peuvent pas s'appliquer à cette molécule. Cependant, du fait même de sa nature, il est peu probable que le rituximab puisse avoir des effets mutagènes.

DP INCOMPATIBILITÉS (début page)

Il n'a pas été observé d'incompatibilité entre MabThera et les poches et tubulures de perfusion en chlorure de polyvinyle ou en polyéthylène.

DP CONDITIONS DE CONSERVATION (début page)

A conserver au réfrigérateur (entre 2 °C et 8 °C). Conserver le conditionnement primaire dans l'emballage extérieur à l'abri de la lumière. La solution diluée de MabThera pour perfusion est physiquement et chimiquement stable pendant 24 heures entre + 2 °C et + 8 °C et pendant 12 heures supplémentaires à température ambiante. Sur le plan microbiologique, la solution diluée pour perfusion doit être utilisée immédiatement. Si elle n'est pas utilisée immédiatement, les durées et les conditions de conservation jusqu'à l'emploi sont sous la responsabilité de l'utilisateur et ne doivent normalement pas dépasser 24 heures entre + 2 °C et + 8 °C, à moins que la dilution n'ait été réalisée dans des conditions d'asepsie contrôlées et validées.

DP MODALITÉS MANIPULATION/ÉLIMINATION (début page)

MabThera est conditionné en flacons stériles à usage unique, apyrogènes et sans conservateur. Prélever de façon aseptique la quantité nécessaire de MabThera et diluer le rituximab dans une poche à perfusion contenant une solution aqueuse apyrogène de chlorure de sodium à 0,9 % ou de glucose à 5 % afin d'obtenir une concentration comprise entre 1 et 4 mg/ml. Pour mélanger la solution, retourner doucement la poche de manière à éviter la formation de mousse. Prendre les précautions nécessaires pour assurer la stérilité de la solution préparée. Le produit ne comportant ni conservateur antimicrobien ni agent bactériostatique, les manipulations doivent se faire de manière aseptique. Avant toute administration, les médicaments destinés à la voie parentérale doivent faire l'objet d'un examen visuel à la recherche de particules ou d'un changement de couleur. Tout produit non utilisé ou déchet doit être éliminé conformément à la réglementation en vigueur.

RENSEIGNEMENTS ADMINISTRATIFS (début page)

LISTE I

Médicament soumis à prescription hospitalière. Prescription réservée aux spécialistes en oncologie, en hématologie, aux médecins compétents en cancérologie, aux spécialistes en rhumatologie ou en médecine interne.
Médicament nécessitant une surveillance particulière pendant le traitement.
La première administration doit être effectuée en milieu hospitalier.

AMM EU/1/98/067/001 ; CIP 560 600.3 (rév 18. 07. 2006) 100 mg.
EU/1/98/067/002 ; CIP 560 602.6 (rév 18. 07. 2006) 500 mg.

Ce document est la propriété des Centres d'Investigation Clinique. Toute reproduction, même partielle, ne peut être effectuée sans autorisation.

Inscrit sur la liste de rétrocession avec prise en charge à 100 %. Collect.
Inscrit sur la liste des spécialités prises en charge en sus de la T2A.
Titulaire de l'AMM : Roche Registration Ltd, 6, Falcon Way, Shire Park, Welwyn Garden City, AL7-1 TW, Royaume-Uni.

ROCHE
52, bd du Parc. 92521 Neuilly-sur-Seine cdx
Info médic et pharma : Tél : 01 46 40 51 91
Pharmacovigilance : Tél : 01 46 40 53 08

Ce document est la propriété des Centres d'Investigation Clinique. Toute reproduction, même partielle, ne peut être effectuée sans autorisation.

ANNEXE F

CONDUITE A TENIR EN CAS D'ALLERGIE AU PRODUIT

Face à l'apparition d'une réaction immédiate durant la perfusion, la première attitude est de ralentir le débit. Si cette manœuvre ne suffit pas à juguler les signes, la perfusion doit être suspendue et si les effets persistent, ils peuvent nécessiter un traitement symptomatique et une réévaluation de la poursuite de la perfusion doit être faite.

Les traitements symptomatiques sont en priorité

-dans les formes bénignes : anti-histaminiques (et éventuel remplissage en cas d'hypotension)

-dans les formes sévères : adrénaline (et éventuel remplissage en cas d'hypotension)

En cas de nécessité, mais en seconde intention, des corticoïdes peuvent être prescrits.

Un équipement et des médicaments d'urgence pour le traitement de ces effets sont gardés à proximité durant le traitement et pendant les deux heures suivantes.

Ce document est la propriété des Centres d'Investigation Clinique. Toute reproduction, même partielle, ne peut être effectuée sans autorisation.

ANNEXE G

Procédure

MISE EN PLACE ET GESTION D'UNE BASE DE DONNEES

**Objet**	Décrire les étapes de la mise en place et de la gestion d'une base de données

**Documents associés**	Contrat type<br>Document listant les modifications de la base<br>Attestation de gel de base<br>Autorisation de diffusion d'une copie de la base

**Référencement**	Procédure de gestion d'une base de données.doc

Date de création	25/01/07	Date de validation	14/02/07	Date de diffusion	14/02/07

**Documentation et références**	

**Rédacteur**	Emmanuel OGER
**Vérificateur**	Nadine BOULAIRE<br>Ghislaine KERMAGORET<br>Anne-Sophie NEDELEC<br>Emmanuel NOWAK<br>Elise POULHAZAN<br>Delphine RIOU<br>Carole ROUDAUT
**Validateur**	Emmanuel OGER

**HISTORIQUE DES MODIFICATIONS**			
**Référencement**	**Date de révision**	**Motif de révision**	**Parties concernées**

☒ **Version contrôlée**	☐ **Version périmée**

Ce document est la propriété des Centres d'Investigation Clinique. Toute reproduction, même partielle, ne peut être effectuée sans autorisation.

SOMMAIRE

I. Abréviations et Définitions **776**

II. Objectifs et Champs d'Application **776**

III. Description **787**

III.1 Logigramme 787

III.2 Les prérequis 798

III.3 Conception et validation du masque de saisie des données 809

III.4 Conception et validation du support informatique de la base de données 809

III.5 Modalités de saisie des données 809

III.6 Modalités de contrôle de la validité des données 809

III.7 Procédures de sécurisation de l'accès à la base de données 81

III.8 Traçabilité des modifications de la base de données 80

III.9 Procédure de gel de la base de données 80

III.10 Modalités de diffusion de copie d'une base de données gelée 80

III.11 Modalités de conservation d'une base de données gelée 81

Ce document est la propriété des Centres d'Investigation Clinique. Toute reproduction, même partielle, ne peut être effectuée sans autorisation.

I. ABREVIATIONS ET DEFINITIONS

**CIC**	Centre(s) d'Investigation Clinique
**CRF**	Cahier de recueil de données (*case report form)*
**TEC**	Technicien(ne) d'étude clinique
**DSMB**	Data and Safety Monitoring Board

II. OBJECTIFS ET CHAMPS D'APPLICATION

Objectifs : Décrire les étapes de la mise en place et la gestion d'une base de données

Champs d'application : Base de données physiquement situé au CIC, constituée à partir de fiches de recueil de données sur support papier.

**Responsable**	
**Destinataires**	

III. DESCRIPTION

III.1 Logigramme

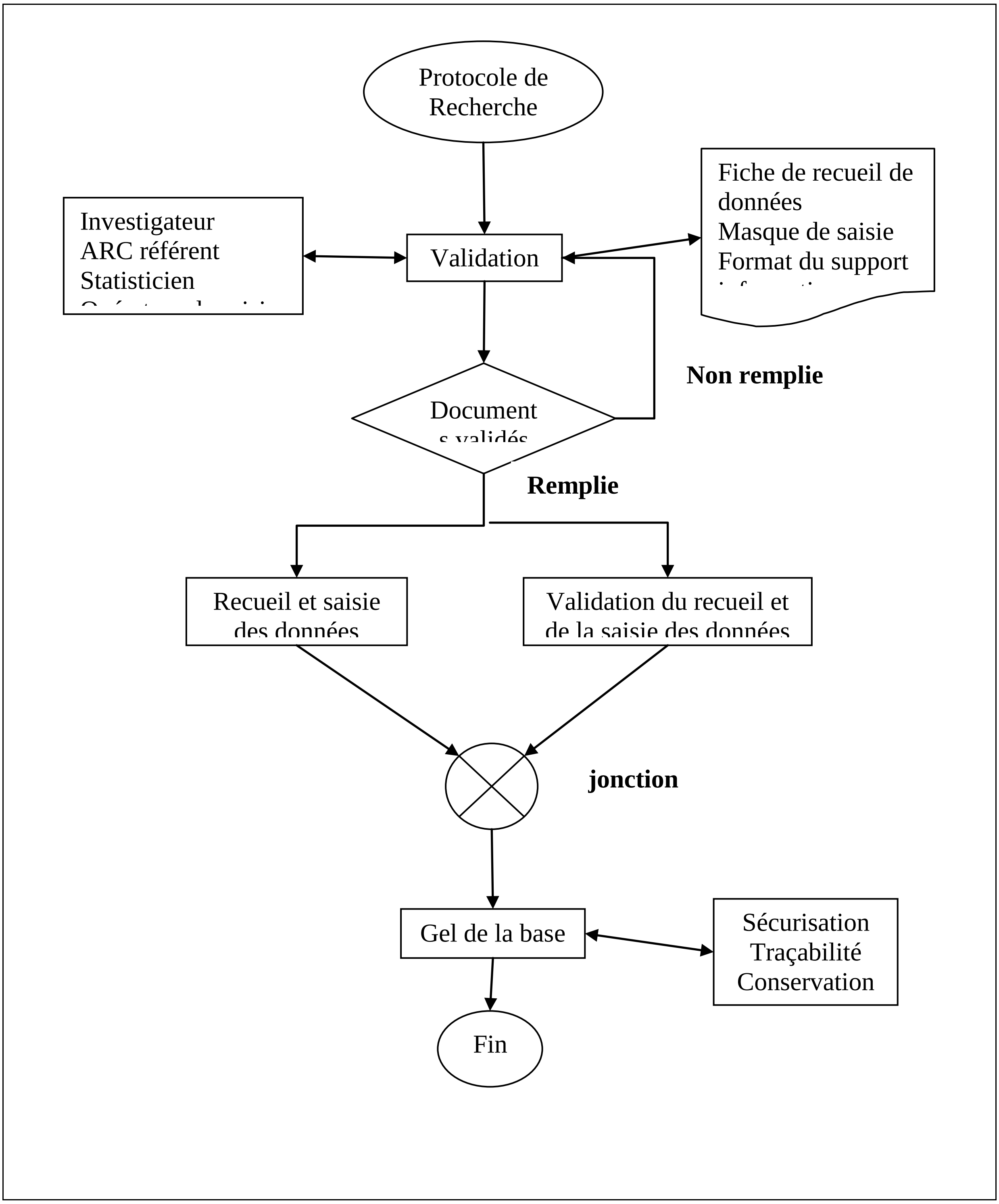

Ce document est la propriété des Centres d'Investigation Clinique. Toute reproduction, même partielle, ne peut être effectuée sans autorisation.

III.2 Les prérequis

Un protocole écrit, finalisé, validé au plan méthodologique avec la désignation d'un statisticien responsable de l'analyse des données et garant de la méthodologie utilisée.

L'identification nominative du/de
- l'investigateur coordonnateur de l'étude
- la/e technicien(ne) d'étude clinique référent(e),

(1) statisticien responsable de l'analyse des données,
(2) responsable de data mangement,
(3) concepteur, réalisateur du masque de saisie.

Un cahier de recueil de données écrit, validé par
(4) le statisticien responsable de l'analyse des données,
- la/e technicien(ne) d'étude clinique référent(e),
- l'investigateur coordonnateur de l'étude

Le cahier de recueil de données, qu'il soit un questionnaire (interview, auto-questionnaire) ou une fiche d'abstraction de données déjà recueillies (et contenu dans le dossier médical par exemple), doit avoir été validé sur un échantillon défini et pour la totalité ou un nombre restreint de variables clés. Les objectifs de cette étude préliminaire sont :

- vérifier l'acceptabilité du questionnaire (par l'enquêteur et la personne interrogée)
- estimer les qualités intrinsèques (sensibilité et spécificité) d'une question/item ou d'un groupe de questions/items portant sur une ou des variables clés : la réponse à la question posée est-elle ce que l'on souhaite mesurer (il est alors indispensable de disposer d'une autre source d'information, idéalement la mesure vraie « étalon-or »)
- estimer la reproductibilité du questionnaire par un deuxième interrogatoire, réalisé idéalement dans les mêmes conditions (le phénomène d'apprentissage est difficile à exclure !)

Le choix d'un logiciel de saisie des données validé par
(1) le statisticien responsable de l'analyse des données,
- l'opérateur(trice) de saisie des données
- et disponible au CIC ou financé dans le cadre de l'étude
- répondant au cahier des charges du CIC (si différent d'EPI-INFO, logiciel de saisie « validé » par le CIC) :
  - interface conviviale,
  - possibilité de mise en place d'un système de contrôle de la saisie
  - possibilité de réalisation d'une comparaison après double saisie

Le choix d'un logiciel support de base des données (si différent du précédent) validé par
(2) le statisticien responsable de l'analyse des données,
- et disponible au CIC ou financé dans le cadre de l'étude

Un financement permettant d'assurer la mission demandée ou l'accord du Comité Technique du CIC pour engager les moyens propres du CIC sur cette mission.

Un engagement écrit de l'investigateur coordonnateur de l'étude à mentionner le soutien logistique du CIC dans les publications, abstract ou poster issus de l'analyse de cette base de données.

Ce document est la propriété des Centres d'Investigation Clinique. Toute reproduction, même partielle, ne peut être effectuée sans autorisation.

Ces informations sont réunies dans un document contractuel signé par l'investigateur coordonnateur de l'étude, le statisticien responsable de l'analyse des données et le coordonnateur du CIC ou le médecin délégué du CIC.

III.3 Conception et validation du masque de saisie des données

A partir du CRF validé, une liste de variables à saisir et leur codage est défini par l'investigateur coordonnateur et le statisticien responsable de l'analyse des données.

Un projet de masque est réalisé par une personne désignée au sein du CIC en utilisant le logiciel défini par dans le contrat susnommé.

Un système de contrôle de la saisie est mis en place.

Le projet est validé par l'investigateur coordonnateur et le statisticien responsable de l'analyse des données avant mise en œuvre de la saisie. Cette validation se traduit par une copie du masque de saisie datée, paraphée à chaque page numérotée et signée par les trois intervenants susnommés.

III.4 Conception et validation du support informatique de la base de données

Dans le cas où le masque de saisie et la base de données ne sont pas issus du même logiciel, les procédures de transfert, définition de nom de variable, codage des variables doivent être définis par le statisticien responsable de l'analyse des données.

III.5 Modalités de saisie des données

S'agissant d'une saisie informatique à partir d'un CRF papier, une double saisie totale ou restreinte à un sous-groupe de variables clés est la règle.

Le CRF doit être validé (daté, signé) par le TEC référent avant saisie informatique et par l'investigateur principal (du centre en cas d'étude multi-centrique).

Le monitoring par le promoteur n'est pas attendu pour cette première saisie.

La deuxième saisie de tous les dossiers a lieu après monitoring et prise en compte des remarques de celui-ci. En cas de monitoring incomplet (c'est-à-dire effectué sur un échantillon aléatoire de CRF et non sur la totalité des CRF), la deuxième saisie est faite sur tous les dossiers qu'ils aient ou non été vérifiés dans le cadre du monitoring du promoteur.

III.6 Modalités de contrôle de la validité des données

Le masque de saisie est choisi dans une liste de logiciels qui permettent d'établir des contrôles lors de la saisie : champ obligatoire, entrée de code contrôlée.

La double saisie est suivie d'une procédure de comparaison des 2 fichiers à la recherche de non concordance.

Une analyse descriptive des données est faite sous la responsabilité du statisticien responsable de l'analyse des données à la recherche de :

Données manquantes,
Données aberrantes,
D'incohérence interne.

Cette mission peut être confiée par le statisticien responsable de l'analyse des données à une personne désignée et appelée alors « responsable de data management ».

III.7 Procédures de sécurisation de l'accès à la base de données

Saisie sur un PC dédié à cette tâche, non connecté au réseau intranet ou internet.
Travail de saisie sur session nominative. La liste des personnes ayant accès aux données est fixée :

- l'opérateur(trice) de saisie des données : écriture autorisée
- la/e technicien(ne) d'étude clinique référent(e : lecture seule
- l'investigateur coordonnateur de l'étude : lecture seule
- le statisticien responsable de l'analyse des données : lecture seule

Mots de passe sur les dossiers renfermant les fichiers de données, différent pour lecture et écriture.

Les accès sont sécurisés de la même façon au niveau des sauvegardes. Les sauvegardes sont faites sur trois supports différents :

(1) Disque dur du PC de saisie : écrasement de la version précédente
Disque dur externe : copie journalière du fichier de données enregistrée en incluant la date, donc un fichier par jour. Rangement du disque dans un lieu d'accès contrôlé différent de la pièce où se situe le PC.
CD : gravure hebdomadaire ou bimensuelle en fonction du nombre de saisie effectuée par semaine. Rangement du CD dans un lieu d'accès contrôlé différent de la pièce où se situent le PC et le disque externe.

Une fois le fichier gravé sur CD, les fichiers enregistrés sur le disque externe correspondant à la période antérieure à la précédente gravure peuvent être effacés. Restent donc sur le disque externe les copies journalières entre deux dates de gravure sur CD.

III.8 Traçabilité des modifications de la base de données

Après monitoring ou contrôle de validité, des demandes de complément (données manquantes) ou de modifications (erreur) sont faites. Ces demandes sont listées sur un document daté, validé par :

- la/e technicien(ne) d'étude clinique référent(e)
- l'investigateur coordonnateur de l'étude
- le statisticien responsable de l'analyse des données

Ces modifications sont ensuite faite sur la base données par l'opérateur(rice) de saisie. Le fichier avant modification et le(s) fichier(s) après modification sont sauvegardés comme précédemment décrits et ne peuvent en aucun cas être effacés.

III.9 Procédure de gel de la base de données

Une fois les modifications prises en compte, une dernière étape de validation des données (voir III.6 à l'exception de la double saisie) est faite.

A l'issue, la décision de gel de base est prise, authentifiée par un document signé par le statisticien responsable de l'analyse des données.

III.10 Modalités de diffusion de copie d'une base de données gelée

Une copie de la base de donnée gelée ne peut être diffusée qu'après accord de l'investigateur coordonnateur et du statisticien responsable de l'analyse des données.

Le transfert d'une copie de la base avant gel, dans le cadre d'une analyse intermédiaire planifiée dans le protocole, ou au statisticien responsable et agissant au nom d'un Comité de monitoring des données (DSMB), est possible sous couvert d'un document d'accord préalable et selon les règles stipulées par le protocole.

III.11 Modalités de conservation d'une base de données gelée

Disque dur externe, spécialement dédiée au stockage sans manipulation.
CD gravé.

Ces deux supports sont protégés en écriture par un mot de passe que seuls les responsables du CIC connaissent.

Stockage dans deux endroits différents avec accès contrôlé : bureau du coordonnateur (CD gravés) et pièce des archives (disque dur externe).

ANNEXE H

BILANS EN LOCAL

Visites V0 (inclusion) et V5 (S24)

- créatinine et – réserve alcaline – TGO – TGP – phosphatases alcalines – gamma GT – CPK – CRP– électrophorèse des protéines , β2 microglobuline

- Sur bandelette urinaire : protéinurie – leucocyturie - hématurie – pH urinaire. En cas d'anomalie, réaliser un dosage de protéines sur les urines de 24 h ainsi qu'une numération des éléments figurés en cytologie.

- NFS – VS

- Taux sérique des immunoglobulines (gammaglobulines, IgA, IgG, IgM) – Dosage du C4

- cryoglobulinémie (tube à 37°C)

- Anticorps anti-nucléaires – DNA natif crithidia – DNA natif Elisa – Anticorps anti SSA et SSB – facteurs rhumatoïdes (Latex, Elisa)

A la visite d'inclusion V0 : Sous population lymphocytaires (CD19/CD3), sérologies hépatites B et C – VIH – CMV à faire en plus et Haemophilus – Pneumocoque conseillé si le patient est vacciné
Biopsie récente des glandes salivaires pour diagnostic fortement conseillée

A la visite V5 : CMV obligatoire et en vue du retraitement Haemophilus et Pneumocoque conseillé.

Visites V1 (S0) et V2 (S2)

- créatinine – TGO – TGP – phosphatases alcalines – gamma GT – CRP

- NFS – VS

Visites V3 (S6) et V4 (S16)

- Créatinine – TGO – TGP – phosphatases alcalines – gamma GT – CRP — β2 microglobuline

- Si anomalie à l'inclusion : réserve alcaline – CPK

- Si anomalie à l'inclusion, sur bandelette urinaire : protéinurie – leucocyturie - hématurie – pH urinaire.

- NFS – VS

- Taux sérique des immunoglobulines (gammaglobulines, IgA, IgG, IgM) – Dosage du C4

- cryoglobulinémie (tube à 37°C)

- Anticorps anti-nucléaires – DNA natif crithidia – DNA natif Elisa – Anticorps anti SSA et SSB – facteurs rhumatoïdes (Latex, Elisa)

Visites V6 (S36) et V7 (S48)

- Créatinine – TGO – TGP – phosphatases alcalines – gamma GT – CRP – β2 microglobuline

- Si anomalie à l'inclusion : réserve alcaline – CPK

- Si anomalie à l'inclusion, sur bandelette urinaire : protéinurie – leucocyturie - hématurie – pH urinaire.

- NFS – VS

- Taux sérique des immunoglobulines (gammaglobulines, IgA, IgG, IgM) – Dosage du C4

- cryoglobulinémie (tube à 37°C)

- Anticorps anti-nucléaires – DNA natif crithidia – DNA natif Elisa – Anticorps anti SSA et SSB – facteurs rhumatoïdes (Latex, Elisa)

A la visite V7 : sous population lymphocytaires (CD19/CD3) centralisé à brest

Post-étude

- Créatinine – TGO – TGP – phosphatases alcalines – gamma GT – CRP – β2 microglobuline

- Si anomalie à l'inclusion : réserve alcaline – CPK

- Si anomalie à l'inclusion, sur bandelette urinaire : protéinurie – leucocyturie - hématurie – pH urinaire.

- NFS – VS

- Taux sérique des immunoglobulines (gammaglobulines, IgA, IgG, IgM) – Dosage du C4

- cryoglobulinémie (tube à 37°C)

- Anticorps anti-nucléaires – DNA natif crithidia – DNA natif Elisa – Anticorps anti SSA et SSB – facteurs rhumatoïdes (Latex, Elisa)

PRELEVEMENTS SANGUINS ENVOYES A BREST

Pour les centres extérieurs : sérothèque à V0 (S0) et V5 (S24)

Prélèvement de **4 tubes secs de 8 ml**. Le sérum est récupéré après centrifugation et aliquoté sur place, sur des tubes MATRIX (500µl/tube) fournis par le CIC de Brest, puis conservé à -80°C (ou exceptionnellement à -20°C, mais à envoyer dans le mois au CIC de Brest) pour envoi groupé à Brest.

Les aliquots seront séparés en 2 boîtes pour chaque patient et stockés sur place, à - 80°C. L'ensemble sera adressé congelé au CIC de Brest sur carboglace, en DEUX envois séparés (afin de minimiser les risques liés au transport), au minimum une fois par an.

Pour les centres extérieurs : dosage des sous-populations lymphocytaires CD19/CD3 à V7 (S48)

Un tube EDTA de 5 ml sera prélevé, conservé à T° ambiante, et envoyé dans les 24 h au CIC de Brest par La Poste (enveloppes spéciales pour le transport de prélèvements sanguins fournies).

Pour les patients inclus à Brest

Des tubes héparinés remplaceront les tubes secs et seront adressés directement au laboratoire d'immunologie de Brest. Ce dernier fera les analyses immunologiques et réadressera les sérums à congeler au CIC.

PRELEVEMENTS DES GLANDES SALIVAIRES ENVOYES A BREST (FORTEMENT CONSEILLE) - VISITES V1 ET V5

Au minimum 4 glandes salivaires seront prélevées et séparées en plusieurs fragments :

- au moins 2 de ces glandes d'une taille minimale de 4 mm^2 seront fixées immédiatement dans une solution tamponnée de formol et envoyées dans les laboratoires d'anatomopathologie des différents centres pour être incluses dans deux blocs de paraffine. Ces blocs de paraffine, conservés à T° ambiante, seront adressés au CIC de Brest au moins une fois par an.
- au moins 2 glandes d'une taille minimale de 4 mm^2 seront placées immédiatement dans 2 tubes de RNA later (tubes fournis) puis conservés à – 80 °C et envoyés au CIC de Brest au minimum une fois par an.

La procédure de stockage du CIC de Brest est jointe dans cette annexe

Procédure

STOCKAGE
DES ECHANTILLONS BIOLOGIQUES

**Objet**	Décrire les étapes du stockage d'un échantillon biologique

**Documents associés**	

**Référencement**	Procédure de stockage des échantillons biologiques

Date de création	09/01/07	Date de validation		Date de diffusion	

**Documentation et références**	

**Rédacteur**	Emmanuel OGER
**Vérificateur**	Lénaick GOURHANT<br>Audrey LEGRAND<br>Elise POULHAZAN<br>Delphine RIOU
**Validateur**	

HISTORIQUE DES MODIFICATIONS			
**Référencement**	**Date de révision**	**Motif de révision**	**Parties concernées**

☒ **Version contrôlée**	☐ **Version périmée**

SOMMAIRE

I. Abréviations et Définitions **88**
II. Objectifs et Champs d'Application **888**
III. Description **87**
III.1 Logigramme 88
III.2 Pré requis 89
III.3 Vérification des échantillons secondaires (aliquotes) 89
III.4 Mise en place des aliquotes sur des boites MATRIX et enregistrement scanner 89
III.5 Saisie des informations de base sur la base de données FREEZER WORKS 89
III.6 Localisation des boites dans les congélateurs 89
III.7 Installation dans les congélateurs 89
III.8 Vérification des stocks et des emplacements 89
III.9 Recherche d'échantillon en vue d'expédition ou de vérification 90

IV. ABREVIATIONS ET DEFINITIONS

**CIC**	Centre d'Investigation Clinique.
**Echantillon biologique**	Tout prélèvement de liquide biologique

V. OBJECTIFS ET CHAMPS D'APPLICATION

Objectifs : Décrire les étapes du stockage d'un échantillon biologique

Champs d'application : échantillon biologique tel que défini, de type prélèvement de sang

**Responsable**	
**Destinataires**	

VI. DESCRIPTION

VI.1Logigramme

Ce document est la propriété des Centres d'Investigation Clinique. Toute reproduction, même partielle, ne peut être effectuée sans autorisation.

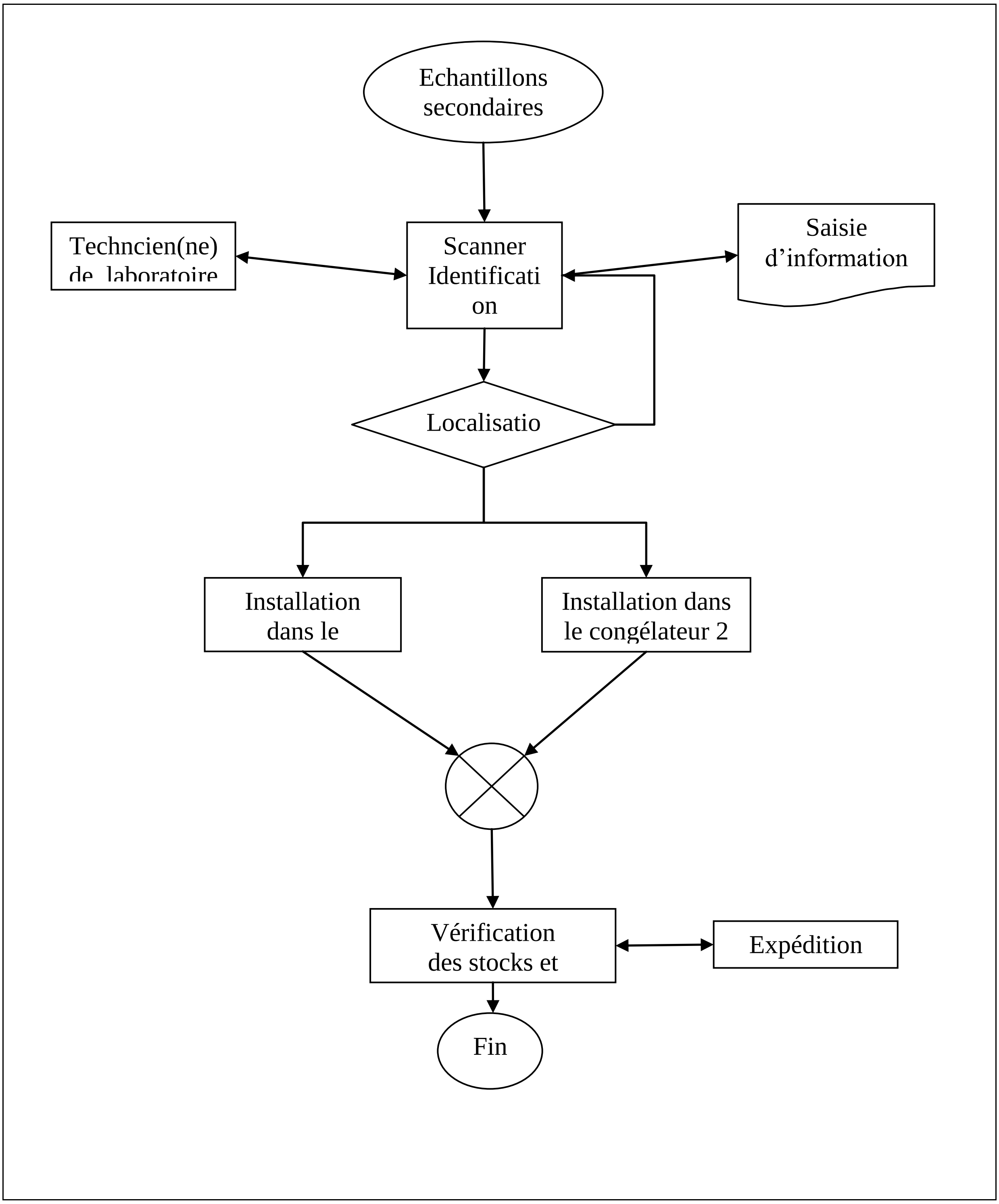
Echantillons secondaires
Techncien(ne) de laboratoire
Scanner Identificati on
Saisie d'information
Localisatio
Installation dans le
Installation dans le congélateur 2
Vérification des stocks et
Expédition
Fin

VI.2 Pré requis

Un protocole écrit, comportant une annexe précisant les modalités de stockage des échantillons biologiques.

Les informations suivantes sont indispensables :

(1) Finalité du conditionnement : stockage de longue durée,…
(2) Nature et quantité des échantillons secondaires à stocker
(3) Conditions de stockage : - 80°C,…
(4) La nature des informations autres que les informations de base à saisir

La mise à disposition d'aliquotes de type MATRIX.

Une fiche technique est préparée pour chaque protocole à partir des informations fournies par l'investigateur ou le promoteur de l'étude (annexe 1).

Tous ces éléments sont écrits dans un contrat signé par l'investigateur principal et le responsable de l'unité de stockage du CIC.

VI.3 Vérification des échantillons secondaires (aliquotes)

Vérification de l'adéquation du nombre et de la qualité des tubes secondaires. Le non respect des contraintes décrites dans le protocole fait l'objet d'une fiche de déclaration d'inadéquation (nombre d'aliquotes insuffisant,…).

VI.4 Mise en place des aliquotes sur des boites MATRIX et enregistrement scanner

Les aliquotes doivent être réparties de façon équitable dans 2 boites différentes.
Les boites sont identifiées et posées sur le scanner qui enregistre leur positionnement et l'identifiant unique gravé au fond de l'aliquote.

VI.5 Saisie des informations de base sur la base de données FREEZER WORKS

Les informations de base suivantes sont renseignées :

1. nom du protocole
2. identification du sujet (selon les modalités propres à chaque protocole)
3. identification du prélèvement (nature : plasma, sérum…, date, numéro de la visite dans la chronologie du protocole)
4. date et heure de mise en place dans le congélateur

VI.6 Localisation des boites dans les congélateurs

Affection d'une position précise (congélateur A à Z, étage A1 à Z4, position dans l'étage…). Chaque boite a donc un identifiant unique, inscrit de manière indélébile.

VI.7 Installation dans les congélateurs

VI.8 Vérification des stocks et des emplacements

Une procédure d'interrogation est faite à la fin de la période d'inclusion pour chaque protocole pour lesquels un contrat de stockage longue durée est passé.

La procédure est calquée sur celle utilisée pour les demandes d'extraction d'échantillon en vue d'expédition.

VI.9 Recherche d'échantillon en vue d'expédition ou de vérification

Une action de recherche d'échantillon est menée sur demande écrite, datée et signée, de l'investigateur principal du protocole (en vue d'expédition) ou du responsable de l'unité de stockage du CIC (en vue de vérification avant édition du rapport annuel).

Cette demande doit comporter les éléments suivants :

1. Liste des identifiants de sujet dont les aliquotes sont à rechercher
2. Nature et nombre d'aliquotes à rechercher par patients

En cas d'expédition, les informations suivantes sont indispensables :

3. Lieu(x) où les aliquotes doivent être expédiés
4. Nom(s) du/des responsable(s) de(s) la structure(s) qui doi(ven)t réceptionner les aliquotes
5. Accord de ce(s) responsable(s) en précisant la date (et l'heure) à laquelle il(s) sont prêts à réceptionner les aliquotes.
6. Modalités de transport (nécessité de carboglace…)
7. Accord du transporteur en précisant la date (et l'heure) à laquelle il est prêt à prendre en charge les aliquotes.

Interrogation de la base de données FREEZER WORKS pour localiser les aliquotes demandés.
Liste ordonnée par congélateur, étagère,… boite.

Préparation d'un boite de transfert.

Mise en place des aliquotes dans la boite de transfert.

Scanner de cette boite et remise immédiate dans le congélateur, dans une zone « de transit ».

Vérification que la liste des aliquotes scannés correspond bien à la liste de demande.

Mise en place de la procédure d'expédition (voir procédure spécifique).

En cas de recherche pour vérification, repositionnement des aliquotes dans les boites d'origine. Un compte rendu de l'opération est fait.
